# Gene expression profiling for human iPS-derived motor neurons from sporadic ALS patients reveals a strong association between mitochondrial functions and neurodegeneration

**DOI:** 10.3389/fncel.2015.00289

**Published:** 2015-08-04

**Authors:** Chrystian J. Alves, Rafael Dariolli, Frederico M. Jorge, Matheus R. Monteiro, Jessica R. Maximino, Roberto S. Martins, Bryan E. Strauss, José E. Krieger, Dagoberto Callegaro, Gerson Chadi

**Affiliations:** ^1^Department of Neurology, Neuroregeneration Center, University of São Paulo School of Medicine, University of São PauloSão Paulo, Brazil; ^2^Laboratory of Genetics and Molecular Cardiology/LIM13, Heart Institute, University of São Paulo School of MedicineSão Paulo, Brazil; ^3^Department of Neurosurgery, Surgical Center of Functional Neurosurgery, Clinics Hospital of University of São PauloSão Paulo, Brazil; ^4^Viral Vector Laboratory, Center for Translational Investigation in Oncology/LIM24, Cancer Institute of São Paulo, University of São Paulo School of MedicineSão Paulo, Brazil

**Keywords:** amyotrophic lateral sclerosis, sporadic ALS, induced pluripotent stem cells, motor neuron differentiation, microarray

## Abstract

Amyotrophic Lateral Sclerosis (ALS) is a fatal neurodegenerative disease that leads to widespread motor neuron death, general palsy and respiratory failure. The most prevalent sporadic ALS form is not genetically inherited. Attempts to translate therapeutic strategies have failed because the described mechanisms of disease are based on animal models carrying specific gene mutations and thus do not address sporadic ALS. In order to achieve a better approach to study the human disease, human induced pluripotent stem cell (hiPSC)-differentiated motor neurons were obtained from motor nerve fibroblasts of sporadic ALS and non-ALS subjects using the STEMCCA Cre-Excisable Constitutive Polycistronic Lentivirus system and submitted to microarray analyses using a whole human genome platform. DAVID analyses of differentially expressed genes identified molecular function and biological process-related genes through Gene Ontology. REVIGO highlighted the related functions mRNA and DNA binding, GTP binding, transcription (co)-repressor activity, lipoprotein receptor binding, synapse organization, intracellular transport, mitotic cell cycle and cell death. KEGG showed pathways associated with Parkinson's disease and oxidative phosphorylation, highlighting iron homeostasis, neurotrophic functions, endosomal trafficking and ERK signaling. The analysis of most dysregulated genes and those representative of the majority of categorized genes indicates a strong association between mitochondrial function and cellular processes possibly related to motor neuron degeneration. In conclusion, iPSC-derived motor neurons from motor nerve fibroblasts of sporadic ALS patients may recapitulate key mechanisms of neurodegeneration and may offer an opportunity for translational investigation of sporadic ALS. Large gene profiling of differentiated motor neurons from sporadic ALS patients highlights mitochondrial participation in the establishment of autonomous mechanisms associated with sporadic ALS.

## Introduction

Amyotrophic lateral sclerosis (ALS) is a neurodegenerative disease that is characterized by a selective death of motor neurons in the brain and spinal cord of adults, leading to rapid respiratory muscle failure and patient death (Cleveland and Rothstein, 2001). Sporadic ALS is not genetically inherited and represents approximately 95% of all ALS cases. A minority of cases are familial forms and are associated with gene mutations (Gros-Louis et al., 2006; Turner and Talbot, 2008).

The generation of transgenic animals that carry mutated human genes associated with familial ALS, for instance superoxide dismutase (SOD1), tar DNA protein 43 (TDP-43), fused in sarcoma, and valosin-containing protein genes, has allowed the identification of basic mechanisms underlying neurodegeneration in the disease (McGoldrick et al., 2013). In fact, distinct processes of motor neuron death that are related to toxic glial paracrine (Nagai et al., 2007; Gowing et al., 2009) and autocrine (Ringer et al., 2012) signaling mechanisms have been described using animal models. Furthermore, the early peripheral pathological events of neuromuscular junction loss and motor axon retraction, which may start long before symptom onset, have also been described in these animal models (Rocha et al., 2013; Venkova et al., 2014). Indeed, recent evidence has pointed out the importance of close morphological and physiological relationships between peripheral non-neuronal Schwann cells (Keller et al., 2009; Chen et al., 2010; Maximino et al., 2014) and fibroblasts (Raman et al., 2015) with motor axons, as key participants in the early degeneration of motor neurons in ALS.

Despite significant progress, it is clear that shortcomings remain with regard to recapitulating the processes underlying the clinical form of sporadic ALS (Benatar, 2007b), based on mechanisms described in transgenic models carrying specific human gene mutations. Actually, these limitations inherent in animal models have been considered the major obstacle preventing the successful translation of results to clinical practice. No therapy has been effective in counteracting human disease progression or extending survival of ALS patients (Benatar, 2007a). Moreover, there is a general consensus that the development of therapeutic targets promoting positive clinical results have been hampered by lack of a relevant human disease model specific to sporadic ALS (Gordon and Meininger, 2011; Veyrat-Durebex et al., 2014).

One of the most promising approaches to provide a better understanding of the mechanisms underlying motor neuron degeneration in sporadic ALS is the generation of specific human-induced pluripotent stem cells (hiPSC) from fibroblasts of motor nerves from sporadic ALS patients. These hiPSCs can be differentiated into multiple cell types, including mature motor neurons which are normally inaccessible for *in vitro* studies (Veyrat-Durebex et al., 2014).

In fact, human motor neurons have been previously generated from human embryonic stem cells and hiPSCs carrying specific gene mutations, all related to the less prevalent, familial form of ALS (Di Giorgio et al., 2008; Marchetto et al., 2008; Mitne-Neto et al., 2011). Despite the substantial effort involved in generating iPSC-derived, differentiated neurons from familial ALS patients, such motor neurons developed from adult cells (i.e., skin fibroblasts and erythroblasts) harbor specific gene mutations, and thus may not represent an appropriate model from which to develop translational therapies specific to the more dominant, sporadic forms of ALS. Thus, this approach is presently restricted to the less prevalent form of ALS (Leblond et al., 2014).

In this study, hiPSC-derived motor neurons were obtained from sporadic ALS patients by reprogramming motor nerve-derived fibroblasts using a STEMCCA Cre-Excisable Constitutive Polycistronic Lentivirus System containing the four transcription factors *OCT4, SOX2, KLF4*, and *CMYC* (Somers et al., 2010). A large gene profiling analyses of these differentiated motor neurons was performed using a high-density oligonucleotide microarray linked to specific tools capable of identifying biological processes, molecular functions and pathways deregulated in the sporadic ALS form.

Our results demonstrate that the generation of hiPSCs by reprogramming motor nerve-derived fibroblasts from sporadic ALS patients, followed by their differentiation into adult motor neurons, is not only feasible, but more importantly, can be used to model key molecular mechanisms which may recapitulate those processes related to neurodegeneration in this disease state. This approach may thus provide one of the most promising platforms for the development of therapeutic targets specific to sporadic ALS.

## Methods

### Human tissue samples

Sporadic ALS patients accompanied at the ALS Ambulatory Unit of Department of Neurology of Clinics Hospital of University of São Paulo School of Medicine signed informed consent and were included in the present study after neurological evaluation. Inclusion criteria were less than 6 months of disease evolution after diagnosis according to EL ESCORIAL (Brooks et al., 2000) and motor impairments not affecting substantially one of the lower limbs. Sporadic ALS patients showed preserved movements of the ankle and big toe in the foot corresponding to the site of biopsy. Exclusion criteria were the presence of associated pathologies, breathing disorders, swallowing and cognitive disorders. Patients were submitted to surgical procedures in one of their feet by means of local anesthesia to collect a fragment of their extensor hallucis brevis nerve which is a motor nerve that innervates the extensor hallucis brevis muscle in the dorsal region of the foot. As control, biopsies were taken from the distal accessory nerve performed during reconstructive surgery of traumatic brachial plexus injuries of non-ALS patients at the Clinics Hospital of University of São Paulo School of Medicine. Non-ALS patients reported no history of familial ALS and clinical evaluation failed to show any signs of ALS. Both procedures were performed in the Surgical Center of Functional Neurosurgery of Clinics Hospital of University of São Paulo in accordance with protocol number 0187/11 approved by the Ethics Committee for Analysis of Research Projects of Clinics Hospital of University of São Paulo School of Medicine.

### DNA sequencing to exclude most frequent gene mutations associated with familial ALS

#### *SOD1* and *TARDBP* analyses

The *SOD1* and *TARDBP* genes were sequenced in ALS patients to verify the absence of DNA mutations in genes normally associated with familial ALS, thus providing further confirmation of the sporadic form of ALS in patients included in the study. Genomic DNA was extracted from peripheral blood using standard methods. For sequencing analysis, primers were designed so that they flanked all five exons of *SOD1* and the first six exons of *TARDBP*, both within intronic regions (Hallewell et al., 1986; Xiong et al., 2010). Direct sequencing of amplified exons was performed using Big-dye® Terminator v3.1 sequencing (Applied Biosystems, USA). Reactions were performed by means of a ABI 3130 genetic analyzer (Applied Biosystems), and sequences were analyzed by using a Mutation Surveyor v5.0 (SoftGenetics, USA).

#### *C9orf72* repeat expansions analysis

Genomic DNA was isolated from peripheral blood according to standard protocols. The repeat number of the GGGGCC hexanucleotides was determined using genotyping primers. Repeat-primed polymerase chain reaction (PCR) was performed in order to provide a qualitative assessment of the presence of *C9orf72* repeat expansions, as previously described (Dejesus-Hernandez et al., 2011). Briefly, 200 ng of genomic DNA were used as template in a final volume of 28 μl containing 12.5 μl of FastStart PCR Master Mix (Roche Applied Science, USA), and a final concentration of 0.25 mM 7-deaza-dGTP (Roche Applied Science), 5% dimetil sulfoxide (Sigma-Aldrich, USA), 1M betaine (Sigma-Aldrich) and 1 μM of each primer. Sample analyses were performed on an ABI 3500 genetic analyzer (Applied Biosystems), and data evaluated using the GeneMapper software. Repeat expansions are known to produce a characteristic sawtooth pattern with a 6-bp periodicity, as previously described (Dejesus-Hernandez et al., 2011).

### hiPS cell generation

Fibroblasts were obtained from fragments of extensor hallucis brevis peripheral motor nerve biopsies from sporadic ALS patients and from distal accessory nerves collected from control non-ALS patients. Fibroblasts of sporadic ALS and non-ALS subjects were submitted to Sendai and STEMCCA methods of reprogramming as described below.

#### Sendai reprogramming

After purification, fibroblasts were expanded in culture (Seluanov et al., 2010) and reprogrammed based on the protocol described by Macarthur et al. (2012). Briefly, approximately 10^5^ human fibroblasts were seeded per well in a 6-well plate and incubated at 37°C and 5% CO_2_. The next day, cells were transduced with the CytoTune iPS Reprogramming Kit containing Sendai virus vectors (Life Technologies, USA; Cat. # A1378001) in a fibroblast growth medium at a multiplicity of infection of 3, as described in the manufacturer's protocols. The medium was replaced with fresh fibroblast growth medium 1 day after transduction and the cells were cultured for 7 days. Cells (2 × 10^5^ cells per dish) were cultured on an inactivated mouse fibroblast feeder layer in 100 mm tissue culture dishes at day 8 of transduction. After an overnight period, the hiPSC medium was replaced daily. The hiPSC medium is composed of DMEM-F12 containing 20% knockout serum replacement (KSR), 1% MEM non-essential amino acids (NEAA) solution, 0.1% 2-mercaptoethanol. Medium was equilibrated at 37°C and added with 4 ng/mL FGF-2 just prior the use. Reagents were purchased from Life Technologies. The hiPSC colonies were picked for expansion and characterization from days 27 to 30 of reprogramming.

#### STEMCCA reprogramming

Fibroblasts of sporadic ALS and non-ALS subject groups were expanded in culture as described above. The reprogramming was performed based on the protocol described by Somers et al. (2010). Briefly, 10^5^ fibroblasts were plated onto a GelTrex (Life technologies) pretreated P35 mm dish in 10% fetal bovine serum and DMEM. The fibroblasts were subjected to reprogramming 24 h after plating by STEMCCA Cre-Excisable Constitutive Polycistronic Lentivirus (Millipore, USA) expressing the pluripotency related genes *OCT4, SOX2, KLF4*, and *CMYC*. DMEM media was replaced by E6 medium (Life Technologies) supplemented with 10 ng/ml human FGF-2 (Life Technologies) (E6F) and 0.5 mM NaB (Sigma-Aldrich) 24 h after transduction. The medium was changed by fresh E6F on days 3, 5, 7, and 9. Subsequently, the medium was changed daily from day 11 to 25 by the E8 medium (Life Technologies) supplemented with 0.25 mM of NaB (Sigma-Aldrich). The colonies were visualized with the aid of a phase contrast microscope at the end of that period and were collected manually. The colonies were then seeded in GelTrex pretreated dishes for the expansion in E8 medium. The hiPSCs-derived from sporadic ALS and non-ALS subjects were characterized by means of immunocytochemistry (Table 1), reverse transcriptase polymerase chain reaction (RT-PCR) and quantitative polymerase chain reaction (qPCR) (Table 2) and alkaline phosphatase live stain described below.

**Table 1:** **Antibodies for characterization of hiPSC, embryoid body, differentiated motor neurons and cardiomyocytes**.

**Antibody**	**Isotype**	**Source**	**Cat. No**.	**Dilution**
**CHARACTERIZATION OF hiPSC**				
SSEA-4	Mouse IgG	Life Technologies	414000	1:100
TRA1-60	Mouse IgG	Life Technologies	411000	1:100
TRA1-81	Mouse IgG	Life Technologies	411100	1:100
Oct4	Rabbit IgG	Life Technologies	A13998	1:100
**EMBRYOID BODY DIFFERENTIATION *IN VITRO***				
Pck-26	Mouse IgG	Abcam	ab6401-100	1:100
Myosin LC 2	Rabbit IgG	Abcam	ab79935-100	1:100
EphA2	Rabbit IgG	Santa Cruz Biotechnology	sc-924	1:100
**DIFFERENTIATED MOTOR NEURONS**				
MAP2	Rabbit IgG	Sigma-Aldrich	M3696	1:100
ChAT	Goat IgG	Abcam	Ab101755	1:100
**DIFFERENTIATED CARDIOMYOCYTES**				
cTnT	Goat IgG	HyTest	4T19	1:200
CD-31	Mouse IgG1	BD Pharmingen	550389	1:100
TRA-1	Mouse IgM	Merck Millipore	MAB4360	1:300
Myo	Mouse IgG1	Abcam	Ab15	1:100

*SSEA-4 (stage-specific embryonic antigen-4), TRA1-60 and TRA1-81 (podocalyxin-like), Oct-4 (octamer-binding transcription factor 4), Pck-26 (pan cytokeratin 26), Myosin LC 2 (myosin light chain 2), EphA2 (ephrin type-A receptor 2), MAP2 (microtubule-associated protein 2), ChAT (choline acetyltransferase), cTnT (troponin T), CD-31 (cluster of differentiation 31), TRA-1 (histone acetyltransferase TRA1), Myo (Myosin). hiPSC were obtained from motor nerve fibroblasts of sporadic ALS and non-ALS subjects*.

**Table 2:** **RT-PCR and qPCR primers for hiPSC and differentiated motor neuron gene markers**.

**Gene**	**Forward 5′–3′**	**Reverse 5′–3′**	**pb**
**hiPSC CHARACTERIZATION**			
*OCT4*	GACAGGGGGAGGGGAGGAGCTAGG	CTTCCCTCCAACCAGTTGCCCCAAAC	144
*SOX2*	GGGAAATGGGAGGGGTGCAAAAGAGG	TTGCGTGAGTGTGGATGGGATTGGTG	151
*NANOG*	CAGCCCTGATTCTTCCACCAGTCCC	TGGAAGGTTCCCAGTCGGGTTCACC	343
*GDF3*	CTTATGCTACGTAAAGGAGCTGGG	GTGCCAACCCAGGTCCCGGAAGTT	631
*ESG/DPPA5*	ATATCCCGCCGTGGGTGAAAGTTC	ACTCAGCCATGGACTGGAGCATCC	243
*DPPA4*	GGAGCCGCCTGCCCTGGAAAATTC	TTTTTCCTGATATTCTATTCCCAT	408
*DPPA2*	CCGTCCCCGCAATCTCCTTCCATC	ATGATGCCAACATGGCTCCCGGTG	606
*REX1/ ZFP42*	AAAGCATCTCCTCATTCATGGT	TGGGCTTTCAGGTTATTTGACT	267
**DIFFERENTIATED MOTOR NEURONS**			
*PAX6*	GCCCTCACAAACACCTACAG	TCATAACTCCGCCCATTCAC	149
*OLIG2*	AGCTCCTCAAATCGCATCC	AAAAGGTCATCGGGCTCTG	380
*CHAT*	TGAGTACTGGCTGAATGACATG	AGTACACCAGAGATGAGGCT	144
*HB9*	GCACCAGTTCAAGCTCAAC	GCTGCGTTTCCATTTCATCC	127
*GAPDH*	ACATCGCTCAGACACCATG	TGTAGTTGAGGTCAATGAAGGG	143

*POUF51 (pou domain class 5, transcription factor), SOX2 (sex determining region Y-box 2), NANOG (homeobox transcription factor nanog), GDF3 (growth differentiation factor-3), ESG/DPPA5 (embryonic stem cell specific gene/developmental pluripotency-associated), DPPA4 (developmental pluripotency-associated protein 4), DPPA2 (developmental pluripotency-associated protein 2), REX1/ZFP42 (reduced expression protein 1/zinc finger protein 42), PAX6 (paired box 6), OLIG2 (oligodendrocyte lineage transcription factor 2), CHAT (choline acetyltransferase), HB9 (homeobox 9) and GAPDH (glyceraldehyde-3-phosphate dehydrogenase). hiPSC were obtained from motor nerve fibroblasts of sporadic ALS and non-ALS subjects*.

#### Alkaline phosphatase live staining

The culture media of hiPSCs from ALS and non-ALS subjects achieved with Sendai and STEMCCA methods of reprogramming were aspirated from colonies and were washed twice with DMEM/F-12 medium (Gibco). The colonies were incubated for 30 min at 37°C with alkaline phosphatase live stain (Life Technologies) diluted in DMEM/F-12 medium (1:500) and then washed 3x with the same medium. Positive stained colonies were visualized by means a FITC filter under fluorescent microscopy (EVOS XL Core Imaging System, Life Technologies).

### Karyotyping

Eighty percent confluent hiPSC colonies were incubated for 2 h in the E8 medium with 0.5 μg/ml colchicine for karyotyping procedures. Cells were then harvested with TrypLE (Life Technologies) and incubated for 20 min at 37°C in 0.075 M KCl solution, fixed in methanol/acetic acid (3:1), placed on pre-wet chilled microscope slides, air-dried, and incubated for 2 days at 37°C. The microscope slides were stained for 90 s with Wright's stain in 2x buffer. Standard G-banding chromosome analysis was performed by Clinics Hospital Molecular Diagnostic Services (São Paulo, Brazil).

### hiPSC differentiation *in vitro* and characterization

Differentiation of induced pluripotent cells was accomplished as described elsewhere (Evans and Kaufman, 1981; Martin, 1981).

#### Embryoid body differentiation

Briefly, hiPSCs were lifted using StemPro EZPassage (Life Technologies) and maintained for 4 days in suspension for embryoid body formation. The cells were cultured in embryoid body medium, composed of knockout DMEM/F12 (Life Technologies), supplemented with 20% KSR (Life Technologies), 2 mM GlutaMAX (Life Technologies), 100 μM NEAA (Life Technologies), 1% antibiotic-antimycotic (Sigma-Aldrich) and 100 μM 2-mercaptoethanol (Life Technologies). Addition of ROCK-inhibitor Y-27632 (Ascent Scientific, UK) for the first 24 h was used to improve the survival of hiPSC single cells (Lai et al., 2010) and to allow an adequate generation of embryoid bodies as well. The embryoid bodies were transferred to Geltrex-coated tissue-treated dishes to allow cell attachment and were maintained there for 1 week until their *in vitro* analysis of differentiation by means of immunocytochemistry with specific markers of three germ layers (Table 1).

#### Cardiomyocyte differentiation

To further analyze their differentiation potential *in vitro*, hiPSCs were induced to directly differentiate into cardiomyocytes. The procedure was performed using a PSC cardiomyocyte differentiation Kit (Life Technologies), according to the manufacturer's protocol. Briefly, after splitting, cells were fed by cardiomyocyte differentiation medium A at day 4. Cells were then fed by cardiomyocyte differentiation medium B on day 6 and were maintained in a cardiomyocyte maintenance medium from day 8 to day 14. At that time point, cells were evaluated for their ability to show contraction as a normal feature of cardiomyocytes (Supplementary Video), characterized by immunocytochemistry for cardiomyocyte cell markers (Table 1) and, stained with fluorescent phalloidin actin binding (Life Technologies) performed according to the manufacture instructions.

### hiPSC differentiation *in vivo*

In order to evaluate the ability of hiPSC to develop teratomas, approximately 3x10^6^ hiPSCs from ALS and non-ALS patients were injected subcutaneously into the dorsal flanks of nude rats anesthetized with ketamin (Cristália, Brazil)-xilazin (Vetbrands, Brazil; 62.5 mg/kg). Teratomas were allowed to expand for about 6 weeks. They were visualized and dissected by means of surgical procedure, fixed overnight in 4% paraformaldehyde and transferred to 70% ethanol until they were embedded in paraffin. Sections were stained with hematoxylin and eosin for microscopy analyses. Protocols for animal use were previously approved by the School of Medicine of the University of São Paulo Institutional Animal Care and Use Committee.

### Motor neuron differentiation and characterization

Motor neuron differentiation was performed according to a protocol described by Hu and Zhang (2009) and modified in our lab. Briefly, after reaching confluence, hiPSC colonies were cultured in suspension in the presence of embryoid body medium to achieve embryoid body formation as described above. The medium was replaced on day 4 by a neural differentiation medium containing DMEM/F12 (Life Technologies), N2-supplement (Invitrogen, USA), 100 μM NEAA (Life Technologies), 1% antibiotic-antimycotic (Sigma-Aldrich) and 2 μg/ml heparin (Cristália) to induce the formation of the neural progenitor cells. Clusters attached to laminin-coated dishes (20 μg/ml, Sigma-Aldrich) after 1 week in suspension. Primitive neuroepithelial cells were posteriorized by addition of 0.1 μM retinoic acid (Sigma-Aldrich) at day 10 and ventralizated by the addition of 100 ng/ml sonic hedgehog (Shh; Sigma-Aldrich) and B27 supplement (Gibco, USA) at day 14. The cells were collected at differentiation day 20 for microarray experiments. Samples of hiPSC-derived motor neurons from ALS and non-ALS subjects were also obtained on small laminin-coated coverslips (13 mm) and characterized by immunocytochemistry (Table 1), RT-PCR, qPCR (Table 2) and Hb9::GFP live reporter.

#### Hb9::GFP live reporter technique for motor neuron visualization

The construct containing Hb9-GFP (Hb9::GFP) reporter was transferred to the differentiated motor neurons using a lentivirus system (Marchetto et al., 2008). Hb9 is a motor neuron-specific transcription factor expressed in mature cells that binds to the promoter region of the green fluorescent protein (GFP) sequence in the construct. HEK 293T cells were employed for virus production. Briefly, triple transfection with calcium phosphate was performed 24 h after plating using the PSPAX2 plasmids, the pCMV-VSVg and the pLenti-Hb9-GFP (Addgene plasmid # 37080) or pEGIP (constitutive lentiviral vector for expression of GFP; Addgene plasmid # 26777) (Marchetto et al., 2008; Zou et al., 2009). The supernatant containing viral particles was collected 24 and 48 h after transfection. In parallel, differentiated motor neurons were plated at a concentration of 2 × 10^6^ cells per dish on 60 mm plates. Transduction was achieved by adding the lentivirus to hiPSC-derived motor neuron cell cultures after the treatment with Shh. The differentiated motor neurons were maintained on small glass coverslips in 24-well plates and transduced using 200 μl viral supernatant plus 100 μl of neural differentiation medium containing 8 mg/ml polybrene. The medium was replaced 6 h after incubation at 37°C and 5% CO_2_. The cells were fixed 72 h after transduction and were evaluated for GFP expression using an Olympus AX-70 microscope (Olympus, JP).

#### Quantification of differentiated motor neurons

The amount of differentiated motor neurons was quantified by the counting of ChAT immunoreactive profiles and also Hb9::GFP profiles counterstained with nuclear DAPI. Briefly, a sample of cells were seeded onto small laminin-coated (Sigma) glass coverslips and immunostained for ChAT (Millipore) or labeled with Hb9:GFP methodology (the construct Hb9::GFP as described above) followed by a DAPI nuclear labeling in order to determine the number of differentiated motor neurons in the sample fields. Evaluation of motor neuron vitality was performed by means of Fluoro-Jade C (FJC) analysis as described in the Figures S4E,F of the Supplementary Material (Schmued et al., 1997, 2005).

### Immunocytochemical characterization

The hiPSC colonies, the embryoid body differentiation *in vitro* and the differentiated motor neurons were characterized by indirect immunofluorescence, as described elsewhere (Maximino et al., 2014). Briefly, cells were fixed with 4% paraformaldehyde and incubated with primary antibodies shown in Table 1. Primary antibodies were detected using Alexa Fluor® 488 or 594-conjugated secondary antibodies specific for mouse, rabbit and goat (all from Invitrogen). Preparations were mounted on microscope slides and counterstained with nuclear 4′, 6-diamidino-2-phenylindole dihydrochloride (DAPI; Vector, USA). Digital images of immunofluorescence staining were obtained by means of an Olympus AX-70 microscope (Olympus).

### RT-PCR and qPCR characterization

Molecular characterizations of hiPSC and of differentiated motor neurons of the chamber culture were performed by means of RT-PCR and qPCR as described elsewhere (Maximino et al., 2014). Briefly, total RNA of cells contained in the 24-well plates was extracted using Trizol (Life Technologies) according to the manufacturer's protocol. The quantity (NanoDrop 1000 Spectrophotometer; Thermo Scientific, USA) and quality (Agilent 2100 bioanalyser, RNA 6000 Pico LabChip; Agilent Technologies, USA) of RNA were determined. cDNAs were amplified by PCR using the primers specific for hiPSC and differentiated motor neurons (Table 2). PCR products were submitted to electrophoresis in 2% agarosis gels containing ethidium bromide for 60 min at 100 volts, and then visualized under ultraviolet light exposure. qPCR reactions were performed as described below. hESC (Fraga et al., 2011) and neural progenitor cells were used as positive controls for hiPSC and negative controls for motor neuron characterization, respectively (Marti et al., 2013; Jha et al., 2015).

### RNA isolation and microarray experiments

The differentiated motor neurons that were achieved from a pool of neuronal progenitor lines of the two sporadic ALS and two non-ALS patients, this process were performed in three different times. A total of three ALS and three non-ALS (control) samples of differentiated motor neurons obtained were subjected to large gene profiling by means of microarray analysis. The same sampling procedure was employed in other quantification methods of the study. The procedures of RNA isolation and the methodology of microarray experiments were described in our previous publication (Maximino et al., 2014). Briefly, total RNA was extracted from differentiated motor neurons and linear amplification of RNA was performed using the RiboampHSplus kit (Arcturus) according to the manufacturer's protocol, control of RNA quality was them assessed, as described above. A representative electropherogram from a Bioanalyzer evaluation of RNA integrity of the differentiated motor neurons is shown in the Supplementary Material (Figure S4H). RNAs of samples (25 ng) and reference (100 ng) were reverse transcribed by the low-input RNA linear amplification kit and then transcribed to Cy5-labeled (samples) or Cy3-labeled (reference) according to the manufacturer's instructions (Agilent Technologies) and to our previous descriptions (de Oliveira et al., 2013). A total of 300 ng of Cy5-labeled cRNA was hybridized together with the same amount of Cy3-labeled reference to Whole Human Genome Oligo 8 × 60 K. All steps were performed according to the manufacturer's instructions (Agilent Technologies) and to our previous descriptions (de Oliveira et al., 2013; Maximino et al., 2014).

### Microarray analysis

The slide were scanned by a Microarray Scanner (Agilent) and the raw image data were converted to numerical data using the Agilent Feature Extraction Software, version 11.0.1.1 (Agilent Technologies), as described in our previous study (Maximino et al., 2014). Microarray raw data (.txt files) were imported into GeneSpring v.13 GX software package (Agilent Technologies). Raw signal intensities were normalized using this software. The probes were tested for differential expression using a moderated t-test for the comparisons between differentiated motor neurons that were achieved from a pool of neuronal progenitor lines of the two control patients and the two ALS patients, using GeneSpring v.13 GX Software. Genes with *p* < 0.05 were accepted as differentially expressed genes. The raw data from hybridizations are available on the Gene Expression Omnibus Database, and the GEO accession number is GSE68240. The data were registered in the GEO according to the following description: control samples (control sample 1, control sample 2, control sample 3) and ALS samples (ALS sample 1, ALS sample 2, ALS sample 3) of differentiated motor neurons that were achieved from a pool of neuronal progenitor lines of the two control patients and the two ALS patients, respectively.

### Functional enrichment analysis

The functional annotation analysis of differentially transcribed genes was performed using the Database for Annotation, Visualization and Integrated Discovery (DAVID) web-server v.6.7 (http://david.abcc.ncifcrf.gov) (Huang Da et al., 2009). Gene Ontology (GO) terms of Biological Process and Molecular Functions, and also Kyoto Encyclopedia of Genes and Genomes (KEGG) pathways included in the DAVID knowledgebase were considered. We have also focused on the GO terms mitochondrion of the Cellular Component and their related genes. Furthermore, the mean of gene expression normalized signals related to the mitochondrion was compared between the two groups. GO terms were submitted to REVIGO, a web server that takes long lists of GO terms and summarizes them in categories and clusters of differentially expressed genes by removing redundant entries (Supek et al., 2011). The analysis was conducted on the list containing the up-regulated and down-regulated genes. High stringency (EASE score) parameters were selected to improve confidence in those values designated as enriched (Ashburner et al., 2000; Kanehisa et al., 2006).

### Quantitative PCR

qPCR was carried out for the analysis the expression of pluripotency markers, *NANOG, SOX2, OCT4*, and *CHAT* in the hiPSCs and also the expression of the *PAX6* (neural stem cell marker), *OLIG2* (motor neuron precursor marker), and the *CHAT, HB9* (mature motor neuron markers) in the differentiated motor neurons. The details of the normalization procedures were described in the legends of the **Figures 2, 5** and Figure S4. Furthermore, a sample of differentially expressed genes described by microarray was selected for verification by qPCR, as described in our previous publication (de Oliveira et al., 2014). The genes were chosen for verification based on their high or low fold relative expression levels and possible involvement in ALS or other neurodegeneration related mechanisms. Thus, dual specificity phosphatase 6 (*DUSP6*), diacylglycerol o-acyltransferase 1 (*DGAT1*), potassium channel, subfamily K, member 12 (*KCNK12*), kinesin family member C1 (*KIFC1*); keratin associated protein 4-11 (*KRTAP4-11*); leucine zipper-EF-hand containing transmembrane protein 1 (*LETM1*); succinate dehydrogenase complex assembly factor 1 (*SDHAF1*); caspase 9, apoptosis-related cysteine peptidase (*CASP9*); vacuolar protein sorting 35 homolog (*S. cerevisiae*) (*VPS35*); and insulin-like growth factor 2 (*IGF2*) were verified by qPCR.

Briefly, cDNA of differentiated motor neurons, as described above was synthesized from 50 ng of total RNA by using a Maxima First Strand cDNA Synthesis Kit (ThermoScientific,USA) according to manufacturer. qPCR reactions were carried out in duplicate with 5 ng cDNA, the DyNAmo Color Flash SYBR Green qPCR kit (Thermo Scientific) and 400 nM of each primer in a final volume reaction of 20 μl, by using the Applied Biosystems 750 Real-Time PCR System (Applied Biosystems). The SYBR primers used in qPCR verification can be found in Table 3.

**Table 3:** **Primers used in SYBR qPCR verification of sporadic ALS motor neuron deregulated genes**.

**Gene**	**Forward 5′–3′**	**Reverse 5′–3′**	**pb**
*DUSP6*	ACAAGCAAATCCCCATCTCG	CAGCCAAGCAATGTACCAAG	123
*DGAT1*	CAACTACCGTGGCATCCTG	TTCTCCAGAAATAACCGGGC	72
*KCNK12*	CTTCTACTTCGTGGGCACC	GCTCCAGGAAGAGGTTGAAG	143
*KIFC1*	TGGTAGTGCTAAGATGCTCATG	ATCCGTCTTCACTTCCTGTTG	144
*KRTAP4-11*	CCCAACCTGTGTCATCTCC	CTCCATGTGTCCTAATAGTCAGC	147
*LETM1*	CAAAGAGAAGGCAGAGAAGGAG	GATGAGCCACTGAGAATCACC	137
*SDHAF1*	CGACAGTCCAAGGAACCC	CCATGCAGTCAGGCGAG	127
*CASP9*	CCAACCCTAGAAAACCTTACCC	TCTGCATTTCCCCTCAAACTC	149
*VPS35*	GTGGCAGATCTCTACGAACTTG	TGGACTGAGGAAATGACTTGAC	106
*IGF2*	CCTGATTGCTCTACCCACC	AACCTGATGGAAACGTCCG	150
*GAPDH*	ACATCGCTCAGACACCATG	TGTAGTTGAGGTCAATGAAGGG	143

*DUSP6 (dual specificity phosphatase 6), DGAT1 (diacylglycerol o-acyltransferase 1), KCNK12 (potassium channel, subfamily K, member 12), KIFC1 (kinesin family member C1), KRTAP4-11 (keratin associated protein 4–11), LETM1 (leucine zipper-EF-hand containing transmembrane protein 1), SDHAF1 (succinate dehydrogenase complex assembly factor 1), CASP9 (caspase 9, apoptosis-related cysteine peptidase), VPS35 (vacuolar protein sorting 35 homolog), IGF2 (insulin-like growth factor 2) and GAPDH (Glyceraldehyde-3-Phosphate Dehydrogenase). hiPSC were obtained from motor nerve fibroblasts of sporadic ALS and non-ALS subjects*.

The cycling in the SYBR reactions was composed of an initial denaturation at 95°C for 10 min. Templates were subsequently amplified by 40 cycles of 95°C for 15 s and 60°C for 30 s. A dissociation curve was then generated to ensure amplification of a single product, and the verification of no primer dimers formation. A standard curve was generated for each primer pair in order to determine the efficiency of the PCR reaction over a range of template concentrations from 0.032 ng/μl to 20 ng/μl, using cDNA synthesized from human differentiated motor neuron reference RNA. The efficiency for each set of primers was 100 ± 5%. Gene expression was normalized to *GAPDH*, and was determined using the ΔΔCt mathematical model (ABI PRISM 7700 Sequence Detection System protocol; Applied Biosystems). *GAPDH* was chosen as a housekeeping gene to normalize the qPCR values because the microarray analysis showed that its expression was stable across samples.

### Statistical analyses

The statistical method employed in the microarray analysis is described above. The One-Way ANOVA was applied with Tukey's multiple comparisons post-test to identify statistical significances between samples within groups. Furthermore, one-tailed unpaired *t*-test was used to determine the statistical significance of differences in qPCR experiments. All analyses were performed using Graphpad Prism 5 (GraphPad, USA). Data were presented as means ± SEM and significance level was set at *p* < 0.05.

## Results

### Clinical evaluation of ALS patients and DNA sequencing

Sporadic ALS patients showed clinical and laboratory features of ALS according to EL SCORIAL (Table 4). The evolution of the disease did not exceed 29 months after initial symptoms and ALS patients showed preserved movements of the ankle and the big toe in the foot corresponding to the site of biopsy. The amplitude of the big toe movement diminished after the biopsy of the extensor hallucis brevis nerve, but they showed a progressive recovery after 4 weeks of surgical intervention. Unfortunately, due the natural evolution of the disease, ALS patients lost movements of the entire inferior limbs in the subsequent months. ALS patients did not present with any cognitive impairments or familial history of neurodegenerative disease.

**Table 4:** **Patient's information**.

	**ALS patients**		**Control patients**	
Patient	Sporadic ALS	Sporadic ALS	Non-ALS	Non-ALS
Age	60	68	43	57
Gender	Male	Male	Female	Male
Biopsy date	August 2013	August 2013	August 2013	August 2013
Date of onset	March 2011	June 2011		
Site of onset	RLL	LUL		
Duration^a^ (months)	29	26		
ALS-FR scale in 2012	34/40	8/40		
ALS-FR scale in 2014	30/40	34/40		
Strength in the unilateral big toe	3/5	3/5		
Electroneuromyography	Chronic disease with anterior tip cervico-thoraco-lumbar	Chronic disease with anterior tip cervico-thoraco-lumbar and bulbar nucleus		
Medicines	Riluzole	Riluzole, B vitamins and venlafaxin		
Additional informations		Depressive symptoms		

*RLL, right lower limb; LUL, left upper limb*.

a *Time from symptom onset to nerve biopsy. Patients from the ALS Ambulatory Unit of Department of Neurology were diagnosed accordingly to EL ESCORIAL and were submitted to a biopsy of extensor hallucis brevis nerve. Non-ALS patients were followed at Neurosurgery Division of Department of Neurology and were submitted to a biopsy of motor branches of accessory nerve during the reconstructive surgery of brachial plexus. The strength in the big toe of 3/5 indicates that functionality of segment was substantially preserved*.

Non-ALS patients were adult subjects that had suffered a unilateral injury of the brachial plexus months before the procedure of plexus surgery. The distal fragments of the preserved accessory nerve were obtained when the nerve was cut and transferred to one of the major nerves of the brachial plexus. That means the donor nerve was functional and did not show signs of local inflammation at the time of surgery.

Sporadic ALS patients showed no *SOD1* and *TARDBP* gene mutations within the coding regions of those genes. Furthermore, *C9orf72* repeat expansions were not present in the sporadic ALS patients (Figure S1).

### Generation and characterization of hiPSCs from motor nerve fibroblasts

Integration-free iPS cell lines were generated using a Sendai virus -based on individual delivery of the 4 Yamanaka reprogramming factors, i.e., *OCT4, SOX2, KLF4*, and *CMYC* (Takahashi et al., 2007). Fibroblasts of all subjects showed successful reprogramming, with no morphological changes observed up to 12 days of reprogramming. The colonies of stem cells were tightly packed, showed a round morphology, and lacked well-defined sharp edges (Figures S2A,B). Unfortunately, several colonies regressed 3 weeks later and expansion of embryonic markers (see below) occurred in only a few colonies. The emerging colonies expressed the surface embryonic stem cell marker, SSEA4, and the pluripotency marker, alkaline phosphatase as shown in Supplementary Material (Figures S2C,D).

Based on the above results regarding the low level of reprogramming achieved by the Sendai method in our laboratory, we subsequently transduced fibroblasts using the STEMCCA Cre-Excisable Constitutive Polycistronic Lentivirus (Somers et al., 2010) in order to increase efficiency. In fact, morphological changes were observed as early as 6 days after reprogramming. Thus, 20 embryonic stem cell-like colonies were picked and expanded under feeder-free conditions 4 weeks after transduction. The best candidates for hiPSC clones from sporadic ALS and non-ALS subjects were selected based on their morphology and were employed in further characterization and motor neuron differentiation experiments.

**Figure 1:**
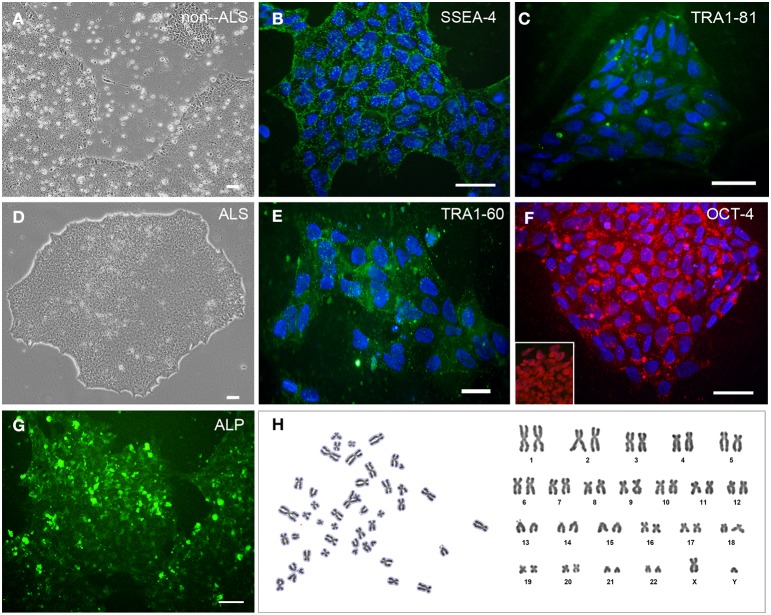
**Generation and characterization of STEMCCA transduced hiPSC**. Primary human induced pluripotent stem cells (hiPSC) colonies derived from non-ALS **(A)** and ALS patients of sporadic form **(B–H)** after transduction with STEMCCA Cre-Excisable Constitutive Polycistronic Lentivirus expressing the embrionary genes *OCT4, SOX2, KLF4*, and *CMYC* after several passages in E8 medium. hiPSCs were derived from fibroblasts of extensor hallucis brevis nerve obtained by biopsy from sporadic ALS patients and from distal fragments of the accessory nerve from a non-ALS subject. Immunostaining of cultured hiPSC for SSEA-4 **(B)**, TRA1-60 **(E)**, TRA1-81 **(C)**, and OCT-4 **(F)**. The presence of OCT-4 immunoreactivity in the nucleus of hiPSC samples that were not counterstained with nuclear DAPI is shown in the box inside of the **(F)**. Of note, OCT-4 has been described in cytoplasmic vesicles **(F)**, an event associated to regulation of pluripotency-associated protein homeostasis of pluripotent cells (Cho et al., 2014; Muratore et al., 2014). Cell nuclei were stained with DAPI (blue). hiPSC colonies were analyzed for alkaline phosphatase activity using the Alkaline Phosphatase Live Stain **(G)**. Karyotyping sampling of hiPSC clone from an ALS patient, in which metaphase plates showed the normal male chromosomal content (**H**; 42,XY). The same pattern was observed for other subjects of the study, including the non-ALS patients (data not shown). Scale bars, 50 μm.

**Figure 2:**
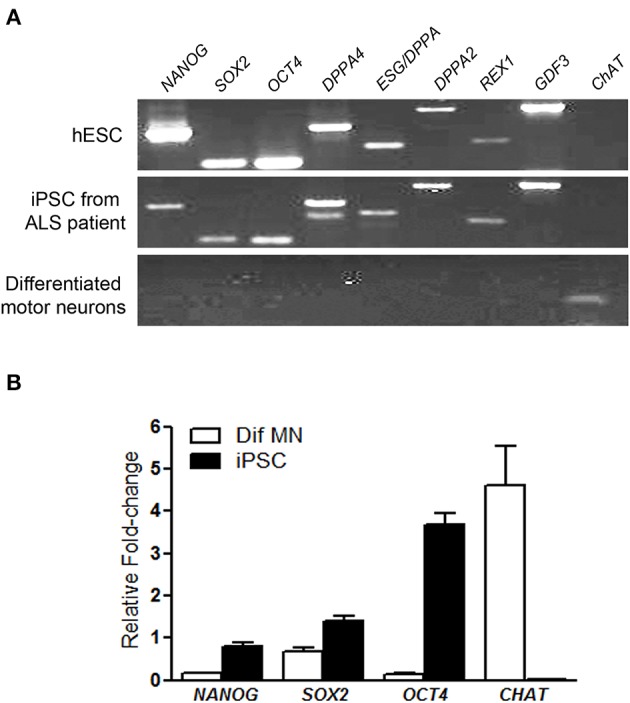
**Pluripotency markers of generated hiPSC**. RT-PCR analysis of pluripotency gene markers in human embryonic stem cells, in induced pluripotent stem cells (iPSC) from motor nerve fibroblasts of an ALS patient and in differentiated motor neurons after *in vitro* differentiation via embryoid body protocol. The pluripotency gene markers, *NANOG, SOX2, OCT4, DPPA4, ESG/DPPA, DPPA2, REX1, GDF* and also the gene choline acetyltransferase (*CHAT*), a marker of differentiated motor neurons were employed **(A)**. The expression of *NANOG, SOX2, OCT4*, and *CHAT* by qPCR in the iPSC and differentiated motor neurons (Dif MN) was quantified and the graph shows relative fold change values; a pool of the hESC was used as reference samples with a reference value of 1 **(B)**. iPSC and hESC show the pluripotency gene markers and differentiated motor neurons show only the *CHAT* marker **(A,B)**. The hESCs were used as a reference parameter.

All hiPSC colonies obtained from sporadic ALS and non-ALS control subjects showed classical morphology and their cells expressed the surface embryonic stem cell markers SSEA-4, TRA1-60, and TRA1-81, as well as the transcription factor OCT4 (Figures 1A–F) and alkaline phosphatase (Figure 1G). Karyotyping analysis of samples of hiPSC lines from all subjects showed an absence of alterations in the hiPSC chromosomal number and morphology (Figure 1H). Furthermore, colonies from ALS and non-ALS subjects showed *NANOG, SOX2, OCT4, DPPA4, ESG/DPPA, DPPA2, REX1, GDF3*, and *CHAT* signals which were similar to those of an hESC line and substantially different from the iPSC-differentiated motor neurons (Figure 2A). Furthermore, hiPSC colonies obtained from sporadic ALS and non-ALS control subjects expressed the higher levels of *NANOG* and *OCT4* as evidenced by the qPCR experiments. Interestingly, the expression of *SOX2*, as seen in the hiPSCs and also in the differentiated motor neurons could be related to the fact that we have not performed the silencing of exogenous factors after lentiviral induction or to a natural time-dependent decrease of these pluripotency factors (Muratore et al., 2014; Raitano et al., 2015) (Figure 2B).

**Figure 3:**
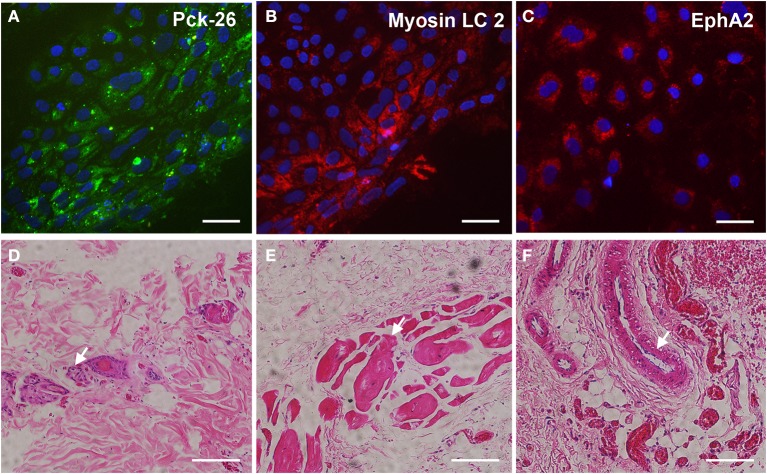
**Spontaneous differentiation of hiPSC into three germ layers ***in vitro*** and ***in vivo*****. Immunocytochemical staining of anti-Pan cytokeratin (**A**; Pck-26, a marker of ectoderm layer), Myosin Light Chain (LC) 2 (**B**; a marker of mesoderm layer) and Ephrin A2 (**C**; a marker of endoderm layer) after spontaneous differentiation *in vitro* of human-induced pluripotent stem cells (hiPSC). hiPSC were derived from fibroblasts which were obtained from motor nerve of sporadic ALS patient. Cell nuclei were stained with DAPI (blue; **A–C**). Hematoxylin and eosin staining of sections from a teratoma formed after a subcutaneous injection of hiPSC into nude rats (**D–F**). Neural rosettes (arrow, **D**), muscle tissue (arrow; **E**), and gut-like epithelium (arrow, **F**) indicate tissues into the teratomas with ectoderm, mesoderm and endoderm features, respectively. Scale bars: 50 μm.

The pluripotency of the hiPSCs from ALS and non-ALS subjects was also evaluated *in vitro* through the formation of embryoid bodies. All hiPSC lines differentiated spontaneously into cell types of the three embryonic germ layers, as indicated by expression of the specific markers; Pck-26 (ectoderm), Myosin LC 2 (mesoderm), and EphA2 (endoderm) (Figures 3A–C).

Furthermore, the pluripotency of hiPSC from ALS and non-ALS subjects was confirmed by the analysis of their ability to develop teratomas *in vivo*, after subcutaneous injection in nude rats. Histopathological analyses demonstrated tissues derived from the three germ layers, including neural rosettes from ectodermal origin, muscle tissue from mesodermal origin and gut-like cells from endodermal origin (Figures 3D–F) in the tumors 6 weeks after injection. These findings indicated a successful reprogramming of fibroblasts to a pluripotent state from sporadic ALS and non-ALS subjects.

### Differentiation of hiPSC into cardiomyocytes

hiPSCs were also differentiated into cardiomyocytes in order to further characterize their potential to generate additional mature cell types for further functional analyses. At 14 days, ALS differentiated cardiomyocytes were present as spontaneously contracting syncytia (Supplementary Video) expressing troponin T cardiac type 2 (cTnT) (Figures S3A,B), but were negative for CD-31 (an endothelial cell marker; Figure S3C), and TRA-1 (a stem cell marker, Figure S3D). hiPSC-differentiated cardiomyocytes expressed positive double-staining against myosin and actin to show the identity and the structural pattern of cardiac differentiated cells (Figures S3E–H).

### Differentiation of hiPSCs into motor neurons

**Figure 4:**
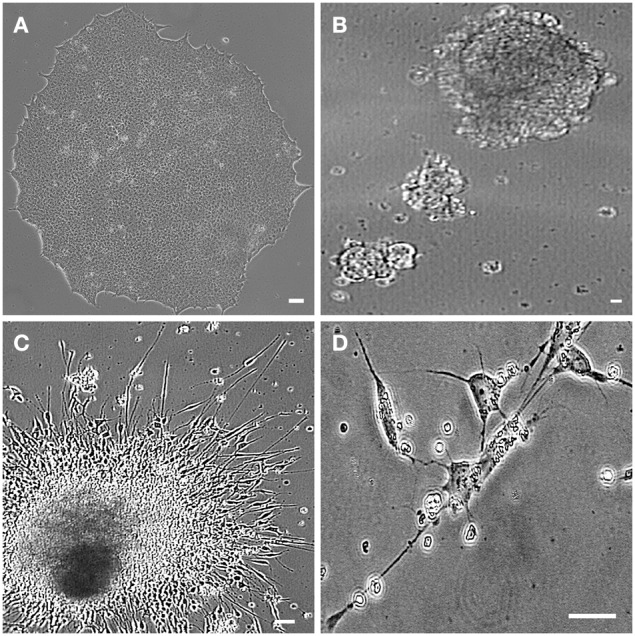
**hiPSC-differentiated motor neurons**. Human induced pluripotent stem cells (hiPSC) sample obtained from motor nerve fibroblasts of a sporadic ALS patient. hiPSC colony growing on feeder free medium forms uniform colonies **(A)**. The motor neuron progenitors are cultured in suspension (embryoid bodies; **B**). Axonal lengths project from the clusters in an adherent culture **(C)**. The hiPSC-derived motor neurons are showing classical morphology with axonal projections **(D)**. Scale bars: 50 μm.

**Figure 5:**
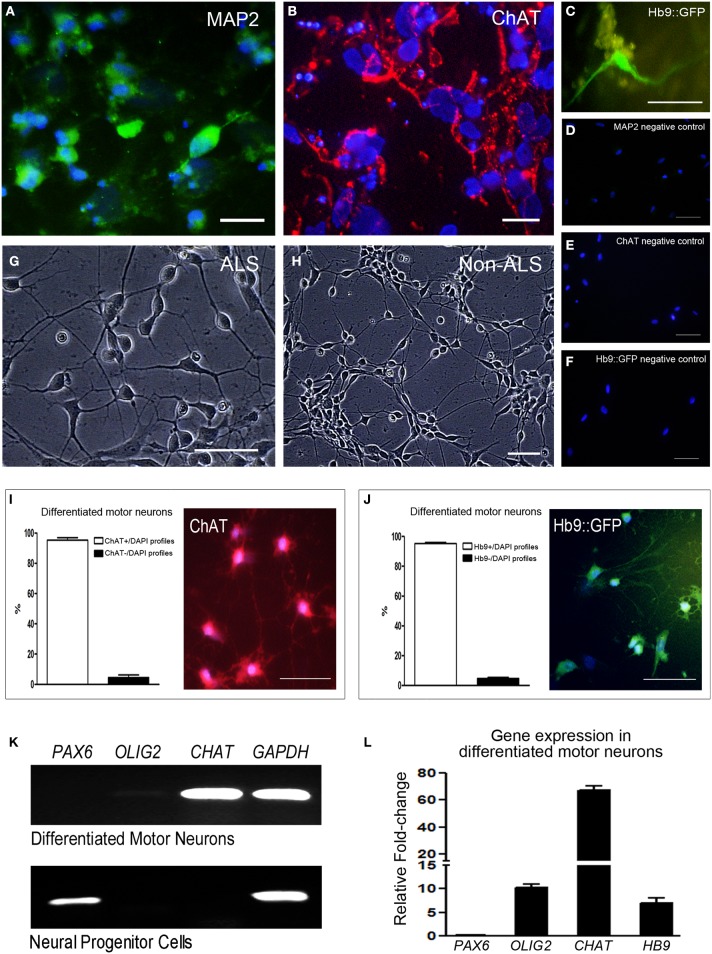
**Specific labeling and qPCR differentiation gene markers of hiPSC-derived motor neurons**. Sample of differentiated human induced pluripotent stem cells (hiPSC)-mature motor neurons are arranged in clusters and show axonal projections. Cells are positive for MAP2 (**A**; green), ChAT (**B**; red) and express GFP under control of motor neuron-specific gene *Hb9* **(C)**. The negative controls for each motor neuron marker mentioned above are seen in **(D–F)**, respectively, and no specific labeling was found. Human induced pluripotent stem cells (hiPSC)-differentiated mature motor neurons from ALS patients **(G)** and their non-ALS controls **(H)** are arranged in clusters and show axonal projections. Number of differentiated motor neurons was quantified by the counting of ChAT+/DAPI profiles **(I)** and Hb9+/DAPI profiles **(J)** in sample regions of the coverslips and the results are expressed as percentage of total cells. Scale bars: 50 μm. RT-PCR analysis of neuronal gene markers of differentiated motor neurons and neural progenitor cells **(K)** contained in the 24-well plates. The markers of *PAX6* (neural progenitor stage), *OLIG2* (motor neuron progenitor stage), *CHAT* (mature motor neuron stage) and *GAPDH* (endogenous control gene) are evidenced. The expression of *PAX6, OLIG2, CHAT*, and *HB9* was quantified in differentiated motor neurons contained in the 24-well plates by qPCR and the graphic shows the relative fold-change values; a pool of the neural progenitor cells was used as reference samples with a reference value of 1 **(L)**.

hiPSC lines from ALS and non-ALS subjects were also manipulated in order to generate mature motor neurons. hiPSC colonies (Figure 4A) first generated embryoid bodies in suspension (Figure 4B) and neuronal progenitor cells. Neural progenitor cells expressing the PAX6 marker (Figure 5K) were differentiated into post-mitotic motor neurons (Figures 4C,D) expressing MAP2 (Figure 5A) and ChAT (Figure 5B). Motor neurons were characterized further by means of a Hb9 (promoter/enhancer)-GFP (*Hb9*::GFP) reporter construct (Figure 5C), an early marker of these maturing motor neurons. Negative controls are shown in Figures 5D–F. There were no differences in the neuronal differentiation process between hiPSCs derived from sporadic ALS or non-ALS subjects. Samples of differentiated hiPSC-mature motor neurons from ALS patients (Figure 5G) and their non-ALS controls (Figure 5H) showed axonal projections. The results of the quantification of the number of differentiated motor neurons in the sample regions of the coverslips revealed the presence of 95.39 and 95.3% of ChAT positive and Hb9 positive profiles, respectively, in relation to the total amount of DAPI nuclear profiles in these regions (Figures 5I,J).

RT-PCR and qPCR analyses of neuronal gene markers of neural progenitor cells and differentiated motor neurons contained in the 24-well plates are shown in the Figures 5K,L and Figure S4G of Supplementary Material. The marker *PAX6* is present only in neural progenitor stage. Despite the very low *OLIG2* signal seen by RT-PCR (Figure 5K), a higher level of signal amplification of *OLIG2* was detected in differentiated motor neurons by qPCR (Figure 5L and Figure S4G of Supplementary Material). Furthermore, qPCR showed high expressions of *CHAT* and *HB9* in the differentiated motor neurons compared to neural progenitor cells (Figure 5L and Figure S4G). Of note, an absence of Fluoro-Jade C staining was found in the 20 day-differentiated motor neurons, indicating that age as an adequate period for molecular study in order to avoid natural stress conditions of *in vitro* analysis.

### Microarray analysis

Microarray analysis demonstrated differentially expressed genes in the hiPSC-derived motor neurons from sporadic ALS and non-ALS subjects (Table S1). Statistical analyses have identified 1591 deregulated genes in differentiated motor neurons from sporadic ALS compared to control subjects. Of these 1591 genes, 526 were up-regulated and 1065 down-regulated in sporadic ALS compared to non-ALS subjects. The complete list of differentially expressed genes can be found in Supplementary Table S1.

### Gene ontology terms grouped by REVIGO

**Figure 6:**
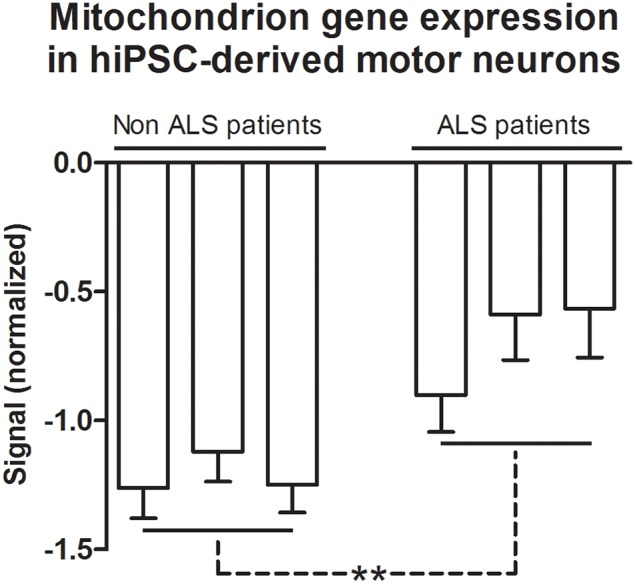
**Mitochondrion gene expression in hiPSC-derived motor neurons**. Gene expression normalized signals of 105 genes from GO terms of Cellular Components that are related to the mitochondrion. Bars represent the means ± SEM of the signals from three samples of differentiated motor neurons of non-ALS and ALS patients, as described in the text. The signal values (Cy5) were normalized by microarray reference (Cy3). The genes referred to GO terms mitochondrion (0005739), mitochondrial part (0044429), mitochondrial matrix (0005759), and mitochondrial lumen (0031980). See text and Table S4 for details. Differences within groups were analyzed by One-Way ANOVA followed by Tukey post-test, whereas differences between non-ALS and ALS groups were analyzed according to unpaired *t*-test ^**^p < 0.01.

Differentially expressed genes based on GO and grouped by REVIGO could be characterized in clusters according to molecular function, e.g., mRNA binding, GTP binding, transcription (co)-repressor activities and low-density lipoprotein particle receptor binding (Figure S5); as well as gene clusters representing various biological processes such as macromolecule catabolism, synapse organization, aging, intracellular transport, negative regulation of DNA binding, death, cell death, mitotic cell cycle and response to inorganic substance (Figure S5). Detailed results for GO terms regarding the GO numbers and number of genes as well as the representative genes in each of these categories can be found in Tables S2, S3 in Supplementary Material. The Cellular Component indicated four GO term related with mitochondrion, specifically the mitochondrion (105 genes), mitochondrion part (65 genes), mitochondrion matrix (30 genes), and mitochondrion lumen (30 genes). The 105 genes of mitochondrion GO term are overlapped with all other GO terms (Table 5). The mean of gene expression normalized signal of 105 genes related to the mitochondrion of differentiated motor neurons from sporadic ALS and non-ALS samples are shown in Figure 6. Of note, the pattern of deregulated gene expression was consistent among the 3 lines of differentiated motor neurons of ALS and non-ALS groups, as exemplified by the absence of statistical differences of the mitochondrion gene expression within the each one of the two studied groups.

**Table 5:** **Genes from GO terms related to the cellular component mitochondrion**.

***ACAA2***	***C1QBP***	***DUT***	***JMJD7***	***MRPS18C***	***NDUFV3***	***SLC25A15***
***ACAD9***	***CAV2***	***E2F1***	***LETM1***	***MRPS22***	***NLRX1***	***SLC25A32***
***ACAT1***	***CHCHD10***	***ELOVL6***	***LIAS***	***MRPS6***	***OPA1***	***SLC25A44***
***ACOT9***	***CKMT1A***	***FANCG***	***LRRC59***	***MTIF3***	***PDHX***	***SOD1***
***ACSM2B***	***COX17***	***GFM1***	***LRRK2***	***MTRR***	***PI4KB***	***STAR***
***AK3***	***COX6A1***	***GFM2***	***LYRM4***	***MUL1***	***POLG***	***STOML2***
***ALDH3A2***	***COX7C***	***GLRX2***	***MACROD1***	***NAPG***	***PPP2R1A***	***SURF1***
***ALKBH3***	***CPOX***	***GLS***	***MCF2L***	***ND2***	***PSMC4***	***TMEM143***
***ASAH2***	***CRAT***	***GRN***	***MMADHC***	***NDUFA12***	***PTPN11***	***TMX4***
***ATP5B***	***CTSB***	***GRPEL1***	***MRPL17***	***NDUFA2***	***ROMO1***	***TOMM20L***
***ATP5C1***	***CYCS***	***HSPD1***	***MRPL23***	***NDUFA4***	***SDHAF1***	***TOMM40***
***ATP5D***	***DBT***	***HTRA2***	***MRPL37***	***NDUFAF3***	***SDHC***	***TSPO***
***ATP5E***	***DIABLO***	***IARS2***	***MRPL46***	***NDUFB5***	***SFXN4***	***VDAC1***
***ATPAF1***	***DNAJA3***	***IDH2***	***MRPL9***	***NDUFB6***	***SIRT3***	***VPS25***
***BZRAP1***	***DTD1***	***IFI6***	***MRPS14***	***NDUFC2***	***SIRT5***	***YWHAZ***

*The Cellular Components GO terms mitochondrion (0005739), mitochondrial part (0044429), mitochondrial matrix (0005759) and mitochondrial lumen (0031980) were composed by 105, 65, 30, and 30 genes, respectively. All genes of GO terms mitochondrion, mitochondrial part, mitochondrial matrix and mitochondrial lumen were also present in the GO term mitochondrion*.

### KEGG analysis

Enriched KEGG terms were identified among differentially expressed genes (*p* < 0.05) in hiPSC-derived motor neurons of sporadic ALS and non-ALS subjects (Table 6). Importantly, differentially expressed genes associated with Parkinson's disease and oxidative phosphorylation are present as over-represented KEGG pathways. These pathways, and respective deregulated genes associated with them, are seen in Table 6. The prostate cancer pathway was omitted from the table because it was composed of genes unrelated to ALS.

**Table 6:** **KEGG pathways obtained from microarray analysis**.

**Gene ID**	**Gene name**	**Gene symbol**	**Fold**
**PARKINSON'S DISEASE**			
842	Caspase 9, apoptosis-related cysteine peptidase	*CASP9*	4.200
7416	Voltage-dependent anion channel 1	*VDAC1*	2.289
514	ATP synthase, H^+^ transporting, mitochondrial F1 complex, epsilon subunit	*ATP5E*	2.184
1350	Cytochrome c oxidase subunit VIIc	*COX7C*	1.928
509	ATP synthase, H^+^ transporting, mitochondrial F1 complex, gamma polypeptide 1	*ATP5C1*	1.887
513	ATP synthase, H^+^ transporting, mitochondrial F1 complex, delta subunit	*ATP5D*	1.865
27429	HtrA serine peptidase 2	*HTRA2*	1.789
4712	NADH dehydrogenase (ubiquinone) 1 beta subcomplex, 6, 17 kDa	*NDUFB6*	1.684
54205	Cytochrome c, somatic	*CYCS*	1.629
1337	Cytochrome c oxidase subunit VIa polypeptide 1	*COX6A1*	1.599
120892	Leucine-rich repeat kinase 2	*LRRK2*	−1.885
506	ATP synthase, H^+^ transporting, mitochondrial F1 complex, beta polypeptide	*ATP5B*	−2.076
4697	NADH dehydrogenase (ubiquinone) 1 alpha subcomplex, 4, 9 kDa	*NDUFA4*	−2.093
4695	NADH dehydrogenase (ubiquinone) 1 alpha subcomplex, 2, 8 kDa	*NDUFA2*	−2.724
4718	NADH dehydrogenase (ubiquinone) 1, subcomplex unknown, 2, 14.5 kDa	*NDUFC2*	−3.004
4731	NADH dehydrogenase (ubiquinone) flavoprotein 3, 10 kDa	*NDUFV3*	−3.109
4536	MTND2	*ND2*	−3.302
7327	Ubiquitin-conjugating enzyme E2G 2	*UBE2G2*	−3.489
6391	Succinate dehydrogenase complex, subunit C, integral membrane protein, 15 kDa	*SDHC*	−7.239
4711	NADH dehydrogenase (ubiquinone) 1 beta subcomplex, 5, 16 kDa	*NDUFB5*	−8.217
**OXIDATIVE PHOSPHORYLATION**			
5464	Pyrophosphatase (inorganic) 1	*PPA1*	2.279
514	ATP synthase, H^+^ transporting, mitochondrial F1 complex, epsilon subunit	*ATP5E*	2.184
1350	Cytochrome c oxidase subunit VIIc	*COX7C*	1.928
509	ATP synthase, H^+^ transporting, mitochondrial F1 complex, gamma polypeptide 1	*ATP5C1*	1.887
513	ATP synthase, H^+^ transporting, mitochondrial F1 complex, delta subunit	*ATP5D*	1.865
4712	NADH dehydrogenase (ubiquinone) 1 beta subcomplex, 6, 17 kDa	*NDUFB6*	1.684
1337	Cytochrome c oxidase subunit VIa polypeptide 1	*COX6A1*	1.599
496	ATPase, H^+^/K^+^ exchanging, beta polypeptide	*ATP4B*	−1.329
10063	COX17 cytochrome c oxidase copper chaperone	*COX17*	−1.863
506	ATP synthase, H^+^ transporting, mitochondrial F1 complex, beta polypeptide	*ATP5B*	−2.076
4697	NADH dehydrogenase (ubiquinone) 1 alpha subcomplex, 4, 9 kDa	*NDUFA4*	−2.093
4695	NADH dehydrogenase (ubiquinone) 1 alpha subcomplex, 2, 8 kDa	*NDUFA2*	−2.724
4718	NADH dehydrogenase (ubiquinone) 1, subcomplex unknown, 2, 14.5 kDa	*NDUFC2*	−3.004
4731	NADH dehydrogenase (ubiquinone) flavoprotein 3, 10 kDa	*NDUFV3*	−3.109
4536	MTND2	*ND2*	−3.302
51382	ATPase, H^+^ transporting, lysosomal 34 kDa, V1 subunit D	*ATP6V1D*	−3.729
6391	Succinate dehydrogenase complex, subunit C, integral membrane protein, 15 kDa	*SDHC*	−7.239
4711	NADH dehydrogenase (ubiquinone) 1 beta subcomplex, 5, 16 kDa	*NDUFB5*	−8.217

*Microarray analyses of hiPSC-derived motor neurons from sporadic ALS and non-ALS subjects. Positive and negative values correspond to up regulation and down regulation fold changes, respectively. The significance of enrichment (EASE score) was considered p < 0.05. The Parkinson disease and oxidative phosphorylation pathways were obtained. The gene names and symbols are seemed*.

### Verification of microarray results by qPCR

**Figure 7:**
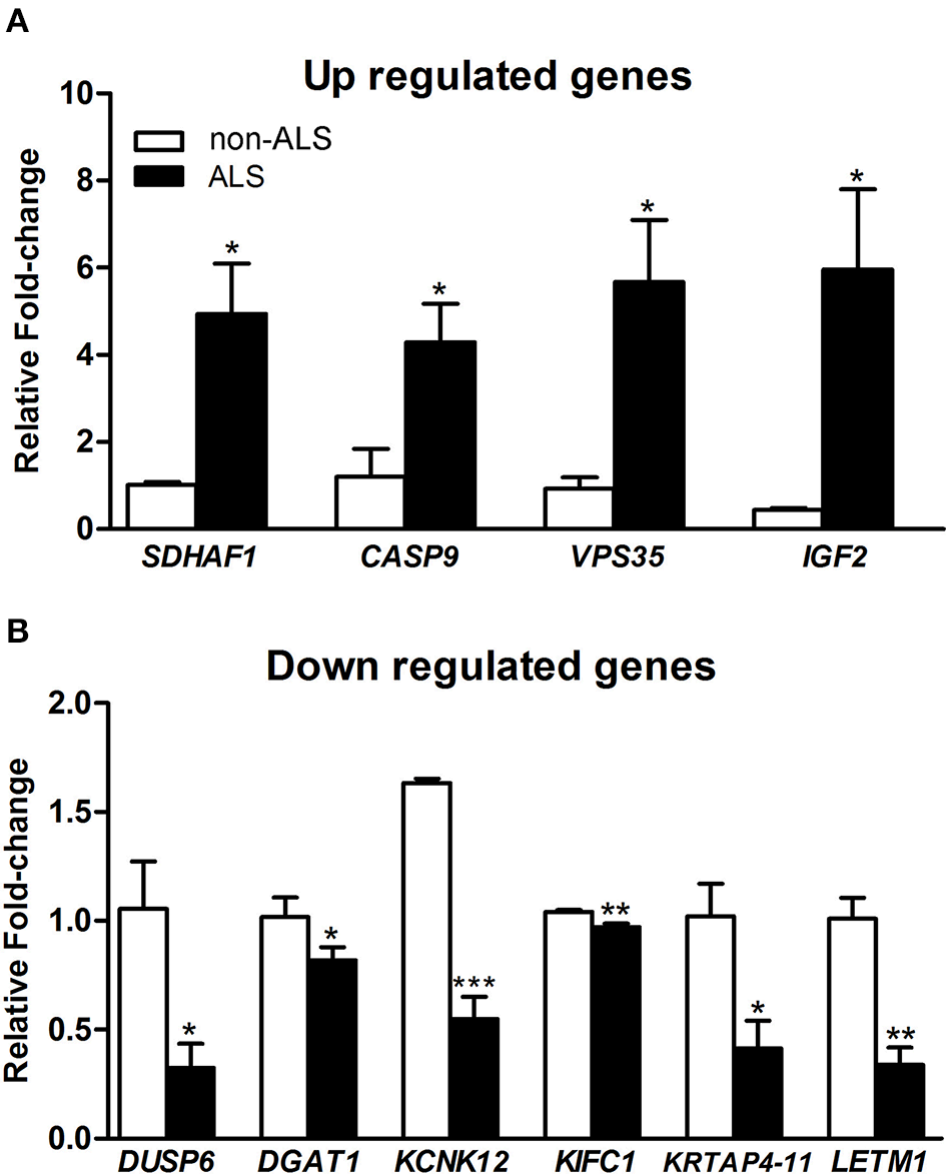
**qPCR verification of deregulated genes**. Graphs show relative fold change values of down regulated **(A)** and up regulated **(B)** of selected genes for verification in human-induced pluripotent stem cells (hiPSC)-differentiated motor neurons from sporadic ALS and non-ALS subjects. hiPSC were obtained from motor nerves of subjects. The selected genes were *IGF2, VPS35, CASP9, HFE*, and *SDHF1* **(A)** and also *DUSP6, DGAT1, KCNK12, KIFC1, KRTAP4-11*, and *LETM1* **(B)**. A pool of the non-ALS group was used as reference samples with a reference value of 1. See text for details. Means ± SEM at least 3 replicates for each group. ^*^, ^**^ and ^***^ *p*-values indicate the < 0.05, < 0.01 and < 0.001 levels of significance, respectively, according to unpaired *t*-test.

The results of qPCR for the 10 representative genes deregulated in differentiated motor neurons from sporadic ALS compared to non-ALS subjects are shown in Figure 7. Both the up (*SDHAF1*; 4.88-fold; *CASP9*; 7.64-fold; *VPS35*; 8.59-fold and *IGF2*; 13.52-fold) and down (*DUSP6*; –3.24-fold; *DGAT1*; –1.23-fold; *KCNK12*; –2.97-fold; *KIFC1*; –1.07-fold; *KRTAP4-11*; –2.46-fold; and *LETM1*; –3.03-fold) regulation observed for these selected genes, as determined by qPCR, were coincident with and supported our microarray results.

## Discussion

Sporadic ALS is a complex disorder and patients present with a wide range of diverse clinical outcomes regarding disease onset, rate of progression and survival (Burkhardt et al., 2013). High throughput gene expression analysis using microarrays has not yet been described for iPSC-derived motor neurons from sporadic ALS patients, thus contributing to a lack of understanding of the mechanisms underlying neurodegeneration in this most common form of that human disease. The absence of clear knowledge regarding the etiopathogenesis and physiopathology of human sporadic ALS has been considered the major cause for failure to translate potential therapeutic strategies to the clinic. Indeed, most experimental studies resulting in therapeutic targets rely fundamentally on experiments using animal models carrying mutated genes of familial ALS (Beghi et al., 2007; Musarò, 2010). Of note, the patients of this study showed an early stage of ALS development, and the biopsy of the motor nerve was performed in a still functioning limb segment, thus the size of samples had to follow the roles of human ethical research. Despite that limitation, the results demonstrated the absence of internal variability.

### Generation of hiPSC-derived motor neurons to study molecular mechanisms in sporadic ALS

Peripheral motor nerves of ALS patients were used for the first time to provide adult fibroblasts, in contrast to numerous previous studies using skin fibroblasts (Almeida et al., 2013; Zhang et al., 2013; Devlin et al., 2015), to be reprogrammed to hiPSCs. Motor nerves, as a source of adult cells for reprogramming, represent a logical choice with which to model ALS particularly since retraction of motor axons and denervation of the neuromuscular junction are the earliest events in the disease (Fischer et al., 2004; Schaefer et al., 2005; Pun et al., 2006). In fact, recent evidence describing neuronal dye-back mechanisms, combined with our own unpublished data, suggest that peripheral cells associated with motor axons, such as Schwann cells, may play a role in motor neuron degeneration in ALS (Keller et al., 2009; Maximino et al., 2014), particularly in the sporadic form. Furthermore, previous microarray studies also showed a differential pattern of gene expression in peripheral fibroblasts from ALS patients (Raman et al., 2015). These non-neuronal cells associated with peripheral motor nerves may provide additional information related to the pathogenic mechanisms of sporadic ALS once hiPSC-derived motor neurons could be produced from them. Of note, the growth and differentiation of peripheral motor nerve fibroblast-derived hiPSCs shown in this work seemed to be comparable to those hiPSCs derived from adult skin fibroblasts described elsewhere (Chestkov et al., 2014).

In support of this approach, in the present study, we initially generated integration-free iPS cell lines using Sendai virus -based individual delivery of each of the four Yamanaka reprogramming factors. However, a greater efficiency was achieved by using STEMCCA Cre-Excisable Constitutive Polycistronic Lentivirus. The combination of all factors in a single transcript using STEMCCA technology may be responsible for the increased efficiency of reprogramming (Awe et al., 2013; Kadari et al., 2014). In our study, this may have allowed the efficient production of hiPSCs from peripheral motor nerve fibroblasts and may have contributed to the subsequent success of motor neuron differentiation. Moreover, as far as we know, it is the first time that the STEMCCA methodology has been applied to generate hiPSC-derived motor neurons.

The potential for motor neuron differentiation achieved by means of the embryoid body method has been previously discussed by Hu and Zhang (2009). The neuronal differentiation process was initiated with the formation of cell aggregates in suspension (free-floating spheres). Furthermore, the subsequent stages involved in the acquisition of the precise motor neuron identities established by the neural progenitor cells, in a process that recapitulated those defined during normal mammalian embryonic development (Dasen and Jessell, 2009). The first step is the acquisition of a “spinal cord” character, i.e., caudalization, by means of extrinsic signals, i.e., retinoic acid. The cell specification along the dorsoventral axis, i.e., ventralization, occurs in response to Shh signaling (Jessell, 2000; Li et al., 2008). After that, differentiating neurons are maintained in the culture medium supplemented with specific neurotrophic factors to undergo further maturation, including the ability to produce specific neurotransmitters and to show specific morphological characteristics of adult motor neurons (Nizzardo et al., 2010). Because our objective was to assess early changes in the gene expression profile of differentiated motor neurons of sporadic ALS patients, microarray analyses were performed on day 20 of differentiation, a time point at which obtained motor neurons already expressed the specific markers ChAT and Hb9. Of note, we did not perform any electrophysiological tests related to mature neuron function on our iPSC-derived motor neurons. However, despite the *OLIG2* expression shown by qPCR, the majority of differentiated motor neurons showed ChAT and also Hb9 markers at that culture age. Also, the cells possessed morphology of mature cells at that time. Thus, the results of *CHAT* and *HB9* by qPCR together with high number of ChAT and Hb9 positive cells in our differentiated motor neuron culture conditions emphasize the state of early mature motor neurons in our system. Furthermore, the expression of *OLIG2* seen in our system has been mentioned previously. In fact, expression of progenitress markers can be encountered in different types of transformed mature cells (Lian et al., 2012). The same should be true for IPSC-derived mature motor neurons because the co-expression of *OLIG2, HB9*, and *CHAT* were found at different stages of maturation (Hester et al., 2011; Jha et al., 2015), a process that may last for several weeks in culture. Nevertheless, the persistent presence of *OLIG2* expression in motor neurons may not impair the differentiation process because generated motor neurons over-expressing Olig2, which were generated by a pLPCX-Olig2 retroviral transfection, were able to acquire morphological, biochemical and electrophysiological properties of mature cells (Lee et al., 2014). Nevertheless, it should be a matter of a next investigation whether a non-excisable system, as it is the case of the present study, may be responsible for the presence of *OLIG2* in the mature differentiated motor neurons. Of note, despite the high number of differentiated motor neurons found in the coverslip, that sample analysis should not be taken as a parameter of reprograming/differentiation efficiency, which was not the scope of the present paper. A highly variable efficiency has been described in the literature, which is due to several factors related to differences of the protocols, iPSCs clones, substratum and also the limitation of the methodology to evaluate the final results (Hu and Zhang, 2009; Takazawa et al., 2012; Amoroso et al., 2013; Qu et al., 2014; Toli et al., 2015). Our qPCR analysis on regulation of transcripts of differentiated motor neuron markers may be a better method to document the type of cultured cell RNA that was evaluated in the microarray.

Furthermore, we decided to evaluate the gene profiling in the 20 day cultured ChAT and Hb9 expressed differentiated motor neurons in order to minimize the stress condition of *in vitro* assay and also because we were interested in the early molecular changes in the disease that could be related to the triggering of motor neuron death. Our decision to study molecular regulation on early mature differentiated motor neurons was also based on the fact that cultured motor neurons from ALS mouse start showing morphological alterations after 14 days in culture (Nagai et al., 2007), the expression of the mature marker may decrease in these cells from culture days 20–30 (Hester et al., 2011), and also because human derived motor neurons could show molecular instability in the process of maturation (Almeida et al., 2013). Despite the early age of differentiated mature motor neurons, that time point seems to be a safer stage to study molecular feature of the disease model in reprogrammed cells. Nevertheless, a detailed analysis of molecular changes at different phases of motor neuron maturation should be a matter of a future investigation.

The adequacy of sporadic ALS-derived iPSC-differentiated motor neurons for molecular studies is also highlighted by our results of the large gene profiling analysis, which is in line with profiles previously described using cells or tissues from post-mortem human ALS subjects or from animal models (Olsen et al., 2001; Dangond et al., 2004; Malaspina and De Belleroche, 2004; Jiang et al., 2005; Perrin et al., 2005; Ferraiuolo et al., 2007, 2011; Yamamoto et al., 2007; Offen et al., 2009; Brockington et al., 2010; D'Arrigo et al., 2010; Guipponi et al., 2010; Saris et al., 2013). Of note, we have not performed the excision of inserted cassette in our hiPSC cell lines to avoid an additional step of cell manipulation, what has not impaired the differentiation of mature Hb9 expressing motor neurons. There is a lack of detailed information regarding the presence and effects of residual reprogramming transgene expression the iPSCs-derived motor neurons. Based on the fact that residual reprogramming transgene expression from integrated viruses may alter the biological properties of iPSCs (Sommer et al., 2012), future work should analyze the gene profiling in hiPSC-derived motor neurons after silencing the exogenous factors. Despite the novelty of the methodological approach, the results of the present gene profiling in the differentiated motor neurons should be taken into attention before *in vivo* experiments because it is difficult to control all consequences of cell reprograming and also to avoid the stress conditions of any *in vitro* studies.

Importantly, the present gene profiling analysis of iPSC-derived motor neurons from sporadic ALS patients provides evidence of possible novel and autonomous mechanisms underlying neurodegeneration in the most abundant form of human ALS. It should be emphasized that previous descriptions on the regulation of gene expression in ALS are based solely on studies with animal models or human post-mortem tissue.

### GO molecular function and biological processes from sporadic ALS motor neuron deregulated genes

The microarray analyses identified 1591 deregulated genes in differentiated motor neurons from sporadic ALS patients compared to control subjects. The differentially expressed genes with a *p*-value lower than 0.05 were submitted to enrichment analyses based on GO and KEGG databases, according to our previous publications (de Oliveira et al., 2013; Maximino et al., 2014). Differentially expressed genes based on GO and grouped by REVIGO displayed molecular functions and biological processes consistent with mechanisms potentially contributing to the autonomous motor neuron cell death characteristic of sporadic ALS.

A wide range of terms of GO molecular function and biological processes grouped by REVIGO have already been implicated in ALS etiopathology. Moreover, their wide ranging and diverse activities underline the high degree of complexity of molecular and biochemical events taking place in the sporadic form of this disease. For instance, within the mRNA binding cluster (Takanashi and Yamaguchi, 2014; Štalekar et al., 2015), *hnRNPA1*, which codes for a heterogenous nuclear ribonucleoprotein A1, was up-regulated, and has been related to frontotemporal dementia and ALS (Seelen et al., 2014). The GTP binding cluster was previously correlated with ALS (Otomo et al., 2003; Droppelmann et al., 2014). However, the possible involvement of the down regulation of *FKBP-4* in the cell-autonomous neurodegeneration characteristic of the sporadic form of ALS is an original contribution of this study. Furthermore, the deregulated *FKBP-4*, which belongs to the immunophilin chaperone protein family, may contribute to anti-aggregation processes of proteins widely described in ALS (Steiner et al., 1997; Gold et al., 1999; Manabe et al., 2002). Of note, the transcription (co)-repressor activity cluster (Tan et al., 2012; Dini Modigliani et al., 2014) revealed by REVIGO, identified *NKX2* as an up-regulated gene product. *NKX2* encodes a natural antisense RNA that modulates the expression of sense transcripts and mRNA processing, and might be involved in intracellular processes related to neuronal degeneration in sporadic ALS (Werner and Sayer, 2009). The synapse organization cluster (Shefner et al., 2006; Venkova et al., 2014) contains up-regulated *MAP1B*, which encodes a molecule linked to microtubule and synaptic stabilization and was found altered in spinal cords from ALS patients (Coyne et al., 2014).

Perhaps most significantly, REVIGO analysis has revealed a possible critical role for mitochondria in the diverse mechanisms contributing to neurodegeneration in ALS. Of note, the synapse organization cluster included the mitochondrion organization category in which the strongly deregulated genes, *LETM1* and *SDHF1*, were identified (discussed below). Also, the intracellular transport cluster (Alami et al., 2014; Foran et al., 2014) contains the highly down-regulated gene, *APOE*, whose product is involved in mitochondrial oxidative stress in ALS, and its variants have been correlated to an increased risk of bulbar-onset of human ALS (Praline et al., 2011). Furthermore, the intracellular transport category also contains the significantly down-regulated *KIF1A* and *KIFC1* genes (also included in several GO terms) whose protein products are responsible for anterograde axonal transport of mitochondria (De Vos et al., 2008; Hou and Yang, 2013), a process that is altered in ALS (De Vos et al., 2008). Additionally, the mitotic cell cycle cluster (Cova et al., 2010) revealed an up-regulation of *DCTN1*, encoding the p150 subunit of the axonal transport protein dynactin. Importantly, mutations in this gene have been associated with sporadic and familial ALS (Münch et al., 2004, 2005).

Significantly, REVIGO analysis provided direct evidence of mitochondrial involvement in the autonomous mechanisms underlying neurodegeneration in ALS with data associated with the cell death and death clusters (Martin et al., 2000; Tapia, 2014). These clusters contained the up-regulated gene, *ATXN10*, which has previously been associated with ALS (Figley et al., 2014), possibly involving mitochondria and activation of caspase 3 (White et al., 2010). Interestingly, the down-regulation of *DYNLL1*, also associated with this cluster, may be associated with impairment of dynein induced retrograde axonal transport of mitochondria in ALS (Chen et al., 2010).

Admittedly, it is difficult to separate the initial triggering from secondary events associated with stress of oncoming neurodegeneration based solely on results of the GO analysis of molecular function and biological process. Moreover, despite a huge diversity of events revealed by GO analysis, the general processes involving mitochondria may represent possible early autonomous mechanisms associated with sporadic ALS motor neurons. It should be stressed that mitochondrial functions also appear in several terms associated with cellular components of GO (data not shown).

### KEGG pathways from sporadic ALS motor neuron deregulated genes

The DAVID analysis identified two specific KEGG pathways that might be related to ALS mechanisms of motor neuron degeneration in sporadic ALS, the Parkinson's disease and oxidative phosphorylation pathways. ALS and Parkinson's disease share some features and mechanisms of neuronal degeneration (Bosco et al., 2011; Shulman et al., 2011), thus emphasizing the relevance of the present molecular analysis employing human motor neurons. In fact, there is growing evidence from work of the past two decades suggesting that ALS may show a complex combination of events at both molecular and cellular levels that are common to Parkinsonism and frontotemporal dementia (Espay et al., 2011). For instance, *TARDBP* mutations (Mosca et al., 2012), TDP-43 proteinopathy (Mackenzie et al., 2010) and *C9orf72* expansion (Lagier-Tourenne et al., 2013) are common to all three pathologies.

The oxidative phosphorylation pathway identified in the KEEG analysis is consistent with mitochondrial participation in sporadic ALS pathology (Ladd et al., 2014). For example, impairments of mitochondrial bioenergetics and energy metabolism, oxidative stress and apoptosis, among other features have been previously described (Cassina et al., 2008; Hedlund et al., 2010; Dupuis et al., 2011; Reddy et al., 2011; Cozzolino and Carrì, 2012; Federico et al., 2012; Brockington et al., 2013; Heath et al., 2013).

The importance of the above two KEEG pathways in the autonomous mechanisms related to neurodegeneration in sporadic ALS is highlighted by recent descriptions of the involvement of their genes in the degeneration of motor neurons in ALS and other neurodegenerative disorders related to ALS. For instance, *LRRK2* mutations are associated with Parkinsonism, ALS and dementia (Wszolek et al., 1997; Zimprich et al., 2004), leading to mitochondrial fragmentation, mitochondrial dysfunction and increased reactive oxygen species (Whittle et al., 2007; Wang et al., 2012). Thus, the down regulation of *LRRK2* in iPSC-derived motor neurons from sporadic ALS subjects in the present study provides further confirmation of the importance of mitochondrial dysfunction in this neurodegenerative disorder.

The down regulation of *ATP5B* we have identified in differentiated motor neurons from sporadic ALS patients in the present microarray analysis correlates with previous findings of reduced cytochrome oxidase and ATP synthase activities described in neurodegenerative disorders (Schägger and Ohm, 1995; Bosetti et al., 2002; Sergeant et al., 2003; Basso et al., 2004; Pamplona et al., 2005). For example, *ATP5B* deregulation was previously characterized in western Pacific ALS-parkinsonism-dementia complex (Shiraki, 1975).

Mitochondrial involvement in the autonomous motor neuron cell death in sporadic ALS is also supported by the up-regulation of *VDAC1* seen in the microarray analyses. *VDAC1* encodes for a voltage dependent anion channel in the outer mitochondrial membrane, and thus is able to regulate local ATP/ADP flux (Colombini, 2004; Lemasters and Holmuhamedov, 2006). Interestingly, VDAC1 is also a key player in mitochondria-mediated apoptosis and has been implicated in apoptotic-relevant mitochondrial events (Shimizu et al., 1999; Shoshan-Barmatz et al., 2006, 2008; Tajeddine et al., 2008; Abu-Hamad et al., 2009; Arbel and Shoshan-Barmatz, 2010). Misfolded mutant SOD1 directly binds and inhibits VDAC1 conductance, thus impacting the onset and survival in the ALS mouse model (Israelson et al., 2010). Furthermore, the deregulation of *VDAC1* may also trigger neuronal damage by a direct interference in the regulation of mitochondrial reactive oxygen species (Liu et al., 2004; Vande Velde et al., 2004) or by a direct activation of mitochondrial apoptotic pathways (Tsujimoto and Shimizu, 2002; Shoshan-Barmatz et al., 2006; Yagoda et al., 2007; Abu-Hamad et al., 2008).

Additional evidence in support of mitochondrial dysfunction in autonomous sporadic ALS is provided by the demonstration that significant up-regulation of *HFE* may be associated with iron homeostasis. *HFE* mutations have been linked to human ALS (Rothstein, 2009; Kwan et al., 2012) and changes in iron metabolism may confer susceptibility to this disease in both humans (Li et al., 2014) and animals (Nandar et al., 2014). Because mitochondria regulate intracellular iron homeostasis (Horowitz and Greenamyre, 2010), *HFE* deregulation may represent an additional mitochondrial-based pathogenic mechanism in sporadic ALS. Mitochondrial dysfunction leading to cell autonomous neuronal damage in sporadic ALS is also supported by the significant deregulation of both *TOMM20L* (Ryan and Gogvadze, 1999) and *HtrA2/Omi* (Srinivasula et al., 2003), as seen in our microarray analysis.

Finally, many of the genes identified as being dysregulated in our microarray analysis have not yet been previously associated with ALS in any context. However, some (eg. *ATP5D, ATP5E, NDUFA4, NDUFB5, NDUFB6)* have been implicated in other neurodegenerative disorders (Chen et al., 2014; Suszyńska-Zajczyk et al., 2014). Additionally, *NDUFC2, ND2, COX7C, NDUFV3, SDHC, ATP5C1, COX6A1, ATP4B, ATP6V1D*, and *COX17* are associated with mitochondrial function or impaired bioenergetic metabolism, and thus further support a role for mitochondrial dysfunction in the cell autonomous degeneration of motor neurons in sporadic ALS (Burman et al., 2014).

### GO mitochondrion genes of the cellular component

Regulation of some of the mitochondrial genes and their related proteins of the cellular component mitochondrion described in the microarray analysis have been mentioned in the context of neuronal degeneration and ALS. For instance, *C1QBP, CAV2, CTSB, DIABLO, GLRX2, NDUFA4, NLRX1, OPA1, SDHC, SIRT3, SURF1* have been correlated to neuronal death and to excitotoxic events (Manfredi and Beal, 2000; Weiergräber et al., 2007; Hayashi et al., 2009; Bräutigam et al., 2011; Gray et al., 2013; Tian et al., 2013; Imbeault et al., 2014). Some of them were related to complex cellular mechanisms of neurodegenerative disorders, also involving other cellular organelles as it is the case of *CTSB* in lysosomal function (Sevenich et al., 2006; Cho et al., 2013). Further indication of the importance of mitochondrion gene regulation in the mechanisms of ALS is highlighted with the descriptions of mitochondrial oxidative stress leading SDHC mutation-induced apoptosis (Ishii et al., 2011) and of neuroprotection after inhibition of mitochondrial release of the pro-apoptotic protein Smac/DIABLO (Soustiel and Larisch, 2010; Luan et al., 2012).

The genes *ATP5B, E2F1, HTRA2, LETM1, LRRK2, POLG, SIRT3, SOD1, VDAC1* have been specifically described in the context of neuronal death in ALS. For instance, the gene polymerase gamma (POLG), which is related to mitochondrial biogenesis, was investigated in the context of ALS (Ladd et al., 2014). The mitochondrial protein ATP5B was also correlated to pathogenesis of neurodegenerative disorders (Yoon et al., 2009), actually the Western Pacific ALS-parkinsonism-dementia complex (Kisby et al., 2006). Mitochondrial proteins involved in cell-cycle and transcriptional regulation, i.e., E2F1, participate in the neuronal death pathways (Wu et al., 2012) and in ALS (Ranganathan and Bowser, 2003). Also, impairments of the apoptotic intermembrane mitochondrial serine protease HTRA2 lead to its accumulation in motor neuronal inclusions in ALS (Kawamoto et al., 2010) and neuronal death (Patterson et al., 2014). Importantly, the mitochondrial Ca^(2+)^ transporter LETM1 is elevated in death resistant motor neurons of ALS mice. Conversely, LRRK2 was implicated in early neuronal death (Chen et al., 2012; Chou et al., 2014) while SIRT3 may exert neuroprotection by avoiding mitochondrial fragmentation in ALS mouse (Song et al., 2013), indicating the complex role of mitochondria in neurodegenerative diseases (Mühling et al., 2014). Furthermore, dysfunction of the mitochondrial voltage-dependent anion channel (VDAC) was associated with neuroprotection failure (Fernandez-Echevarria et al., 2014) and neurodegeneration in ALS (Israelson et al., 2010). Of note, a specific pathogenic *LRRK2* mutation was described in ALS (Whittle et al., 2007). It should be noticed that the role of SOD1 in mitochondrial mechanism related to ALS has been largely described (Dupuis et al., 2004).

Finally, the *PSMC4, GRN*, and *ND2* encoded genes of mitochondrial proteins have also been described in ALS, as it is the case of the 26S proteasome regulator PSMC4 (Trippier et al., 2014). Interestingly, a co-occurrence of GRN/C9ORF72 (Testi et al., 2015) and of NADH dehydrogenase subunit 2 (ND2) mutations were also detected in ALS patients (Lin et al., 1992).

It should be noticed that mutations of *CHCHD10* have been described in ALS, a gene that encodes an intermembrane mitochondrial protein involved in mitochondrial genome stability and morphology (Bannwarth et al., 2014; Johnson et al., 2014).

It is well known that the regulation of mitochondrial gene expression is not restricted to pathological conditions but instead to a large range of cellular physiological conditions. However, we have demonstrated in our work no variations of mitochondrial gene expression within the groups but a significant difference between non-ALS and ALS subjects, emphasizing a possible participation of deregulated genes in the motor neurons disease. Actually, statistical significance between groups and small intragroup variability indicated an homogeneity of the samples.

### qPCR verification of sporadic ALS motor neuron deregulated genes

The results of the qPCR and microarray analyses correlated well for all selected genes, confirming the efficacy of large gene profiling in the search for therapeutic targets in neurodegenerative disorders (de Oliveira et al., 2013; Maximino et al., 2014). Notably, a relationship between these deregulated genes with various molecular/biological processes related to intracellular trafficking, intercellular signaling, and neurotrophic functions (Vucic and Kiernan, 2009; Martin, 2012; Chaturvedi and Flint Beal, 2013) is supportive of a role for mitochondrial dysfunction in ALS, as previously postulated for other neurodegenerative diseases (Pagano et al., 2014) Furthermore, a critical analysis of the up and down regulated genes by qPCR supports their roles in both the events initiating neuronal damage and subsequent reactive events leading to neuroprotection.

Of note, *LETM1*, which encodes a mitochondrial Ca^2+^/H^+^ exchanger protein (Waldeck-Weiermair et al., 2011; Nowikovsky et al., 2012) was found to be up-regulated in iPSC-developed motor neurons of sporadic ALS patients. This mitochondrial ion exchange protein has been linked to protection of rescued neurons in the ALS mouse model (Mühling et al., 2014), possibly by its ability to regulate Ca^2+^ homeostasis. The high down regulation of *LETM1* in sporadic ALS motor neurons supports a role for mitochondrial dysfunction in the cell-autonomous failure of neuroprotection. Moreover, *DUSP6* was the most significantly down regulated gene identified by the microarray analyses. This gene encodes for a dual specificity phosphatase-6 protein, which is a critical determinant of ERK signaling (Bhalla et al., 2002), and is thus able to influence cell survival after neurotoxicity (Hetman and Gozdz, 2004). As ERK signaling has been implicated in ALS (Kim and Choi, 2010), a strong down-regulation of *DUSP6* in sporadic ALS motor neurons is worthy of further investigation. Of note, despite of microarray and qPCR have shown a down regulation of *DUSP6*, the degree of regulation demonstrated by microarray was higher than that obtained by qPCR analysis The use of qPCR analysis to qualitatively verify the microarray results is largely accepted in the literature. However, small discrepancies in the degree of gene regulation between the two methods have been reported (Ferraiuolo et al., 2007). These methods have their specific quantitative differences (Chuaqui et al., 2002), which are thought to be related to the variation in the hybridization kinetics of the technologies, low fold changes or lack of concordance between transcripts accessed in each method (de Oliveira et al., 2013).

Dysfunction in lipid metabolism has also been associated with ALS (Schmitt et al., 2014; Palamiuc et al., 2015). The present study is the first to demonstrate a significant deregulation of *DGAT1*, a gene which encodes a multipass transmembrane protein that functions as a key metabolic enzyme in the conversion of diacylglycerol and fatty acetyl CoA to triacylglycerol, in the context of human ALS.

Also, specific processes involving potassium channels have been described in non-autonomous glial mechanisms of ALS (Bataveljić et al., 2012; Sato et al., 2014). However, this work contributed to the original description of this potassium channel's (subfamily K, member 12 gene, *KCNK12*) deregulation in differentiated human motor neurons from sporadic ALS patients.

The contribution of kinesins in the deregulated axonal transport (Landers et al., 2009; Shi et al., 2010; Kuzma-Kozakiewicz et al., 2013) as well as the participation of cytoskeleton components (Maximino et al., 2014) in ALS have been well documented. However, the significant down regulation in the genes, *KIFC-1* and *KRTAP4-11*, point to the possibility of abnormalities in intracellular trafficking and cytoskeletal structural changes respectively, in the differentiated motor neurons from sporadic ALS patients.

The up-regulation of *CASP9*, which encodes for caspase-9 protein, might activate mitochondria-dependent apoptotic signaling, an event that has been described in neurodegenerative diseases (Andreoli et al., 2009; Darwish and Amiridze, 2010) including ALS (Pasinelli et al., 2000; Guégan et al., 2001, 2002; Inoue et al., 2003). Interestingly, caspase-9 levels have been correlated with clinical severity in ALS patients (Ilzecka, 2012). Considering the disease-specific phenotype of the iPSC-derived motor neurons in the present analysis, an early dysfunction of caspase-9 mediating motor neuron death may take place in sporadic ALS. Furthermore, the up regulation *SDHAF1*, which encodes for succinate dehydrogenase complex, an enzyme that participates in the respiratory chain and Krebs cycle (Rutter et al., 2010) further implicates mitochondrial participation in sporadic ALS.

Conversely, distinct from the putative roles of *CASP9* and *SDHAF-1*, the up-regulation of *IGF2* and *VPS35* may indicate the occurrence of reactive autocrine neuroprotective responses in the sporadic ALS-differentiated motor neurons after an initial damage. Thus, it is likely that up-regulation of *IGF2* may be involved in neuroprotection of motor neurons (Leventhal et al., 1999; Silva et al., 2009), in line to its previously suggested role in non-autonomous glial ALS mechanisms (Kihira et al., 2007; Dagvajantsan et al., 2008). The fact that IGF-1 treatment led to neuroprotection in an ALS animal model (Jablonka et al., 2011; Saenger et al., 2012) and to partial benefits in ALS clinical trials (Lai et al., 1997; Borasio et al., 1998), further supports our finding that up-regulation of *IGF2* expression in the differentiated motor neurons from sporadic ALS patients highlights the importance of IGF family proteins as potential therapeutic targets for this disease. Accordingly, the regulation of *VPS35* corroborates our findings with *IGF2*. The *VPS35* gene product is involved in retrograde transport of proteins from endosomes to the Golgi complex (Zhang et al., 2001). *VPS35* mutations and disruption of endosomal trafficking has previously been implicated in other neurodegenerative disorders (Small et al., 2005; Zimprich et al., 2011; Ando et al., 2012; Vilariño-Güell et al., 2014; Perrett et al., 2015). Thus, up-regulation of *VPS35* might account for a neuroprotective response in ALS before degeneration of motor neurons begins.

In conclusion, the large gene profiling of early stage mature motor neurons differentiated from hiPSCs, which in turn, were derived from adult motor nerve associated fibroblasts from sporadic ALS subjects, has provided evidence for a wide range of molecular and cellular mechanisms possibly involved in the cell autonomous neuronal death in the disease. Of note, evidence supporting impairment of intracellular trafficking, intercellular signaling, oxidative phosphorylation and neurotrophic factor function among others, strongly implicate mitochondrial dysfunction as a key factor in this cell-autonomous neurodegeneration process. Interestingly, evidence is also provided that autocrine neuroprotective mechanisms coexist with those related to cell toxicity in these human sporadic ALS differentiated motor neurons. These results thus emphasize the importance of the use of human iPSC-derived motor neurons, obtained from motor nerve fibroblasts, as a reliable method to model disease mechanisms associated with neuronal degeneration in sporadic ALS. Importantly, the approach may also provide a viable opportunity to translate these results to the development of potential therapeutic targets specific to sporadic ALS.

## Author contributions

CA, RD, MM, and JM performed the experiments. FJ, GC, RM, and DC selected the patients and performed the biopsies. CA, JM, BS, JK, and GC designed the study, analyzed the results and wrote the manuscript. GC is responsible for the ALS Brazil Project of the University of São Paulo School of Medicine. All authors read and approved the final manuscript.

### Conflict of interest statement

The authors declare that the research was conducted in the absence of any commercial or financial relationships that could be construed as a potential conflict of interest.
