# Supplementary material for: Gene expression profiling for human iPS-derived motor neurons from sporadic ALS patients reveals a strong association between mitochondrial functions and neurodegeneration
Source: Front Cell Neurosci. 2015 Aug 4;9:289. doi: 10.3389/fncel.2015.00289 (PMC4523944; doi:10.3389/fncel.2015.00289)
Supplement: Supplementary file 2 [file DataSheet1.PDF]

Supplementary Material for:

Gene expression profiling for human iPS-derived motor neurons from sporadic ALS patients reveals a strong association between mitochondrial functions and neurodegeneration

Chrystian Junqueira Alves[1], Rafael Dariolli[2], Frederico Mennucci de Haidar Jorge[1], Matheus Rodrigues Monteiro[1], Jessica Ruivo Maximino[1], Roberto Sergio Martins[3], Bryan Eric Strauss[4], José Eduardo Krieger[2], Dagoberto Callegaro[1], Gerson Chadi[1*]

(Alves CJ, Dariolli R, Jorge FMH, Monteiro MR, Maximino JR, Martins RS, Strauss BE, Krieger JE, Callegaro D, Chadi G)

1 Neuroregeneration Center, Department of Neurology, University of São Paulo School of Medicine, University of São Paulo, São Paulo, Brazil.

2 Laboratory of Genetics and Molecular Cardiology/LIM13, Heart Institute, University of São Paulo School of Medicine, São Paulo, Brazil.

3 Surgical Center of Functional Neurosurgery, Department of Neurosurgery, Clinics Hospital of University of São Paulo, São Paulo, Brazil.

4 Viral Vector Laboratory, Heart Institute, University of São Paulo School of Medicine, São Paulo, Brazil.

*Corresponding author:
Gerson Chadi. M.D., Ph.D.
Full Professor
Department of Neurology
University of São Paulo
Av. Dr. Arnaldo, 455, 2nd floor, room 2119
01246-903, São Paulo. Brazil
Phone: 55 11 3061-7460
gerchadi@usp.br

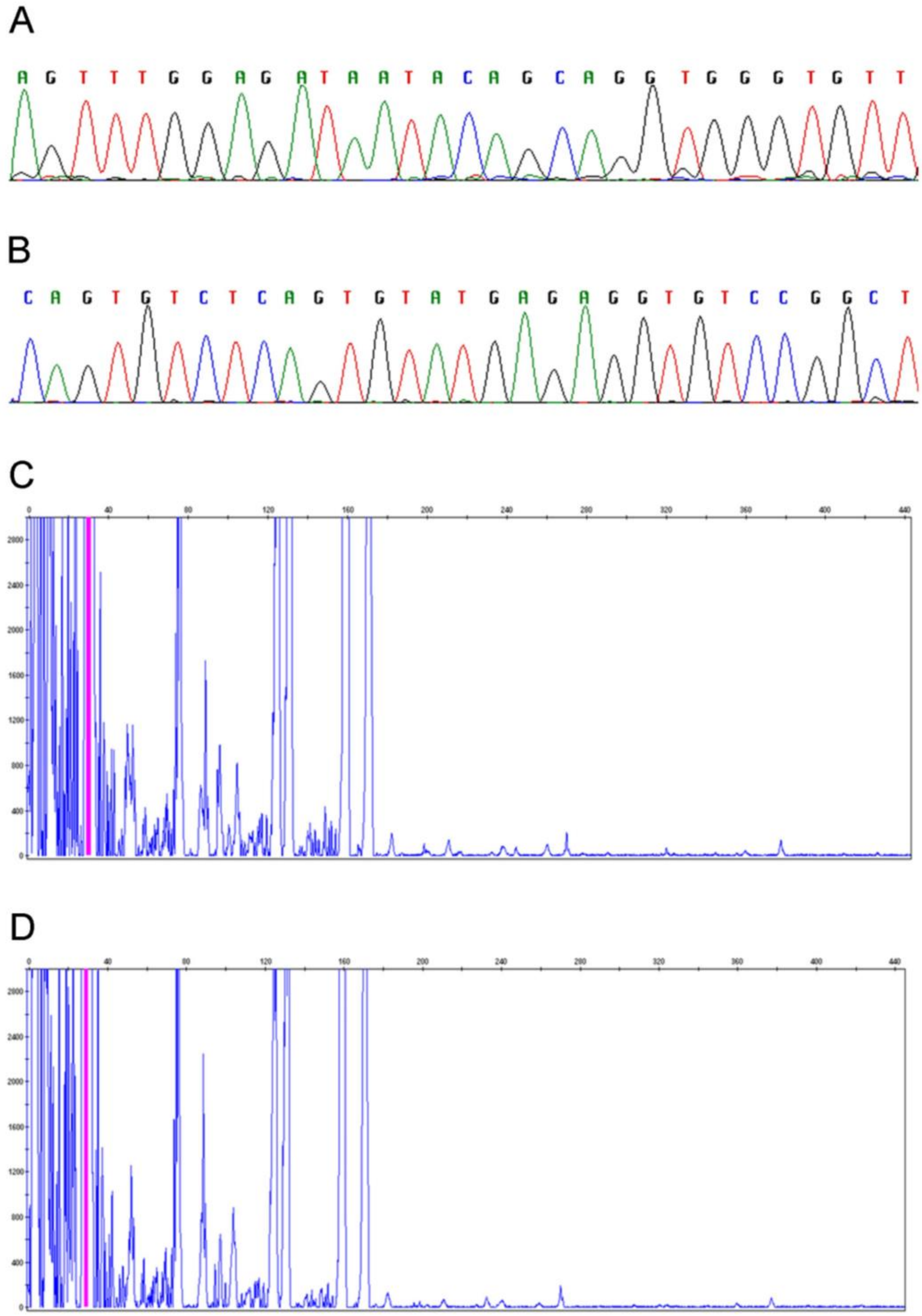

Figure S1. *SOD1* and *TARDBP* DNA sequencing and *C9orf72* repeat expansion analyses. Samples of sequencing chromatograms without mutations for *SOD1* (A) and *TARDBP* (B) gene of a sporadic ALS patient illustrating a random region of exon 2 of the both genes. Samples of *C9orf72* repeat expansion analyses of two sporadic ALS patients (C, D) showing no expansions above 30 repeats. No mutations in the evaluated genes were found in the subjects of the present study.

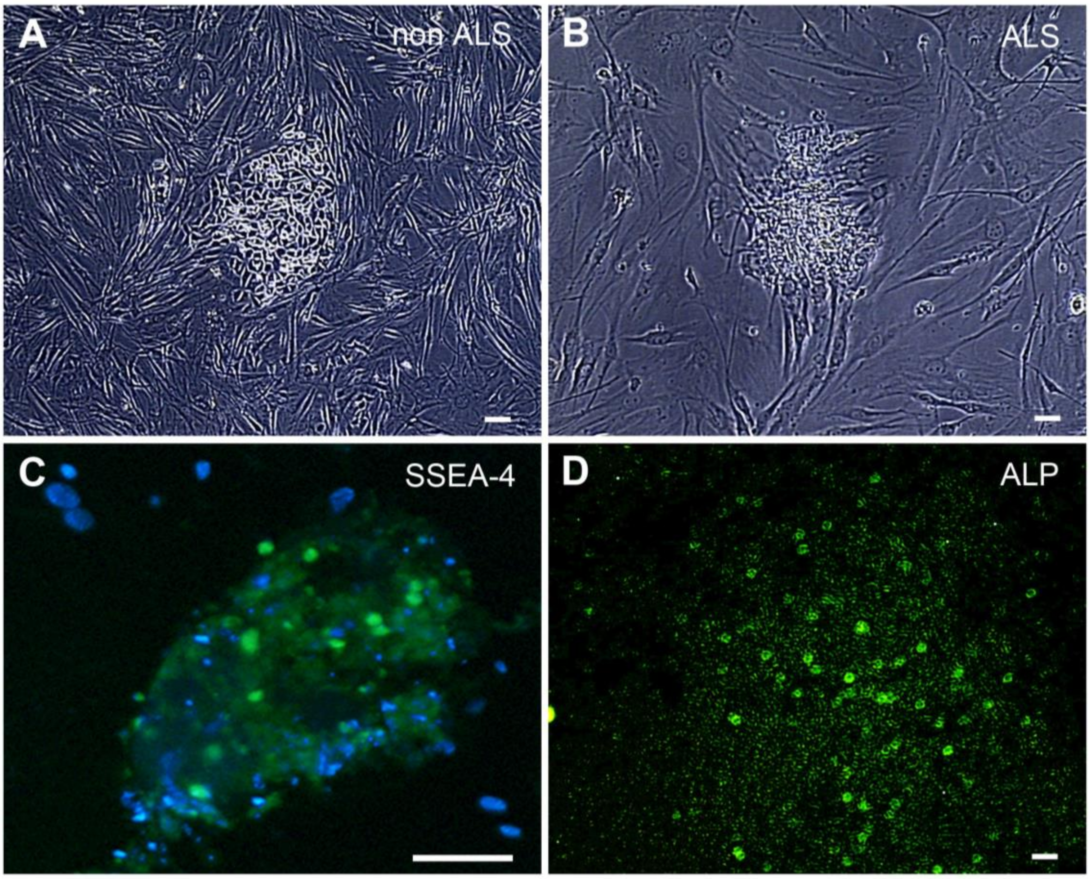

Figure S2. Generation and characterization of SENDAI transduced hiPSC. Primary hiPSC colonies derived from a non ALS (A, C) and a sporadic ALS (B, D) subjects after transduction with CytoTune iPS Reprogramming Kit containing Sendai virus vectors expressing the embrionary genes Oct4, Sox2, Klf4 and cMyc separately. hiPSC were reprogrammed from fibroblasts obtained from motor neurons of ALS and non ALS subjects as described in the text. hiPSC colonies were photographed under phase contrast (A, B). Immunostaining of cultured hiPSC for SSEA-4 and cell nuclei stained with DAPI (blue, C). Sample of a hiPSC colony was also analyzed for alkaline phosphatase (ALP) activity using the Alkaline Phosphatase Live Stain (D). Scale bars, 50 µm.

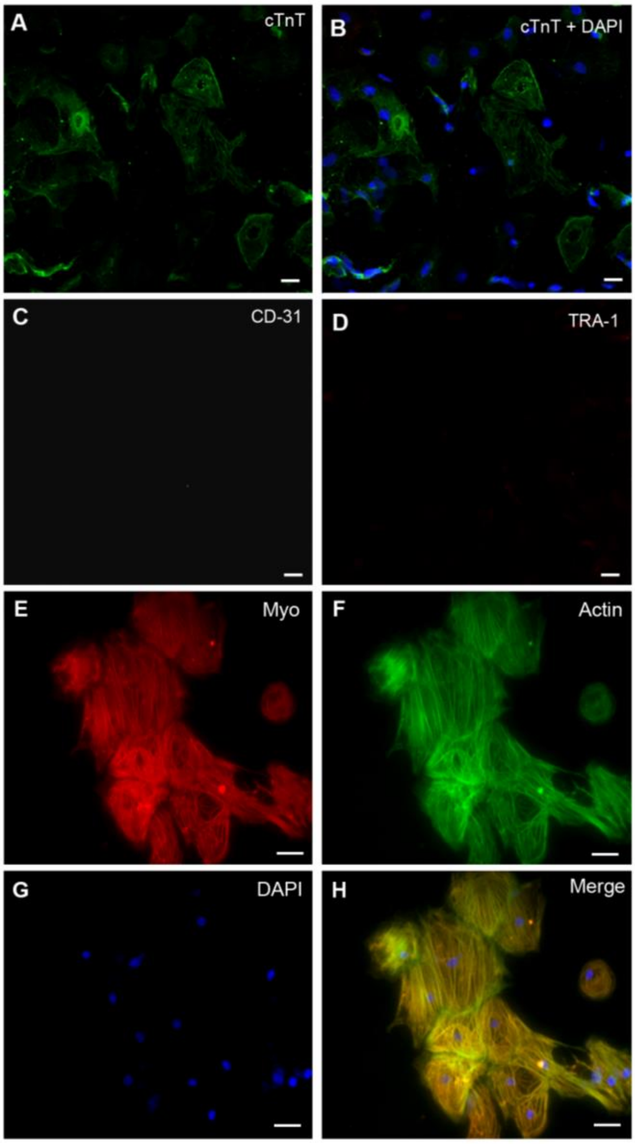

Figure S3. hiPSC differentiated cardiomyocytes. A sample of cultured hiPSC-differentiated cardiomyocytes from a sporadic ALS patient showing immunopositive staining for Troponin T (green; cTnT, a cardiomyocyte marker; A, B) and also the nuclei marker DAPI (blue; B). Same cells showed negative labelings for CD-31 (an endothelial marker; C) and for TRA-1 (an embrionary stem cell marker; D). hiPSC were reprogramed from fibroblasts obtained from extensor halluces brevis nerve of ALS patient of sporadic form. In addition, a sample of cultured hiPSC-differentiated cardiomyocytes showing positive double-staining against myosin (red; Myo; a cardiomyocyte marker; E) and actin (green; Actin; a cardiomyocyte marker; F). The nuclei were marked with DAPI (blue; G) and the images were merged (yellow; H) to show the identity and the structural pattern of cardiac differentiated cells. Scale bars, 20 µm (A-D) and 50 µm (E-H).

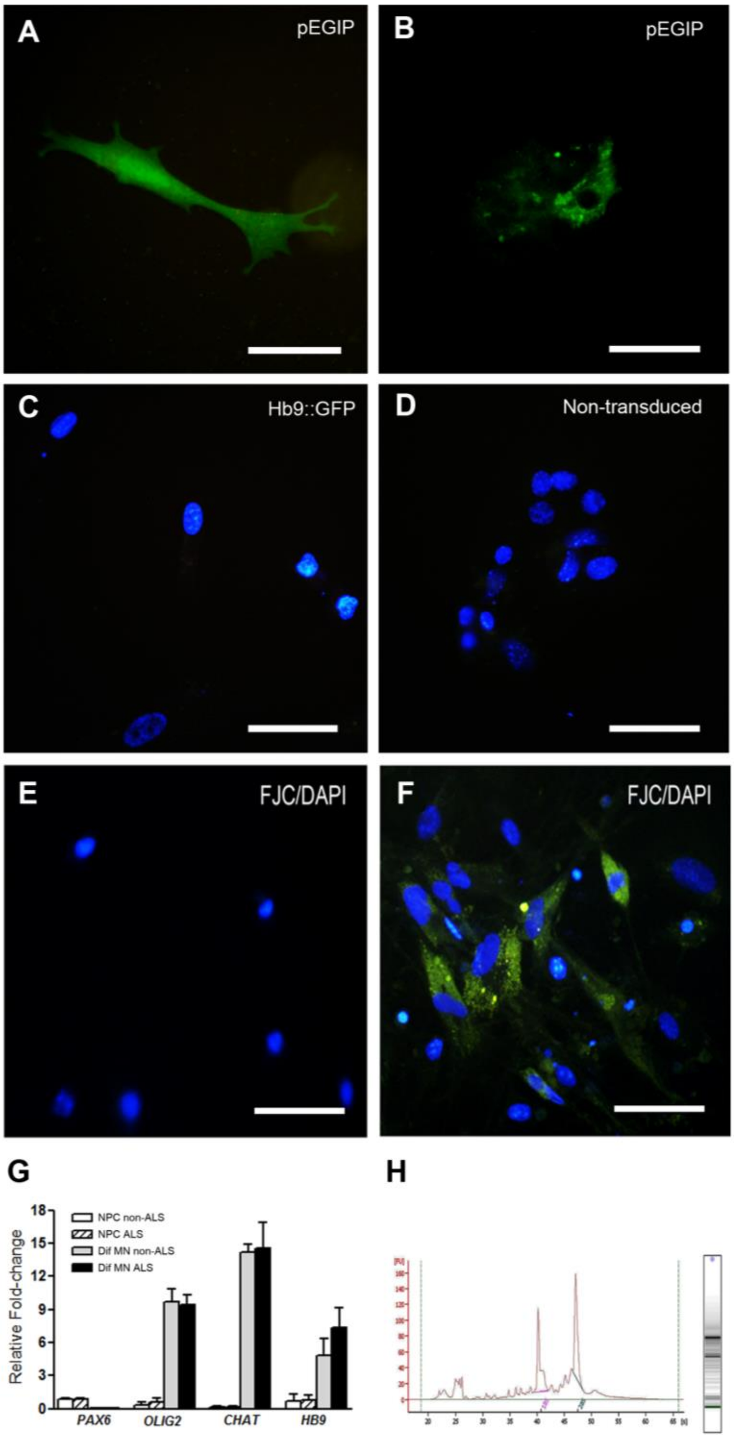

Figure S4. Hb9::GFP, vital state and motor neuron markers. GFP expression (A,B) by a constitutive lentiviral vector (pEGIP) in human fibroblast (A) and differentiated motor neuron. The absence of GFP expression (C,D) is seen in fibroblast under control of specific gene *Hb9* (C; Hb9::GFP) and in a non transduced motor neuron (D). The vital state of differentiated motor neurons was verified by means of a Fluoro-Jade C (FJC) marker at 20 day-period of motor neuron differentiation in culture. The method is based on the ability of acidic Fluoro-Jade C, an anionic highly acidic derivative of fluorescein (Schmued et al., 2005), to bind in specific intracellular basic targets of non vital injured neurons which will develop neuronal degeneration (Schmued et al., 1997). hiPSC-differenciated motor neurons (E) derived fibroblasts from extensor halluces brevis nerve of an ALS patient of sporadic form were submitted to FJC staining and nuclear DAPI labeling. The absence of FJC labeling in the 20 day-cultured motor neurons indicate the vital state of the cells

(E). FJC is seen in primary spinal cord motor neurons of $SOD1^{G93A}$ ALS mice after six days *in vitro* (F). Scale bars: 50µm. qPCR verification of neural and motor neuron gene markers (*PAX6*, *OLIG2*, *CHAT* and *HB9*) in the neural progenitor cells (NPC) and differentiated (Dif) motor neurons (MN) from sporadic ALS patients and their controls (G; non-ALS). A pool of the neural progenitor cells (non-ALS and ALS groups) was used as reference samples with a reference value of 1. Means ± SEM at least 3 replicates for each group. Representative electropherogram of RNA integrity of a sample (iPSC-differentiated motor neurons from sporadic ALS patient) employed in the experiments (H). Calculation was based on the ratios between 28S and 18S subunities from ribosomal RNA and the RNA integrity number (RIN) was equal 8 (values of all samples ranged from 7.0 to 8.9).

Fluoro-Jade C labeling in differentiated motor neurons

The cells were plated onto 13-mm coverslips were fixed and stained with Fluoro-Jade C (FJC; Millipore), a specific marker of neuronal degeneration (Schmuck et al., 2009). Briefly, cultured cells were washed twice in PBS and fixed with 4% formaldehyde (Synth) for 30 minutes. After, fixed cells were washed with distilled water, permeabilized with 0.3% Triton X 100 (Sigma) in PBS for 10 minutes and exposed to Fluoro-Jade C at 0.001% final concentration for 30 minutes in the dark. The cells onto coverslips were washed 3 times with distilled water for 5 minutes and then placed on slides with mounting medium containing DAPI (Montex; Vector).

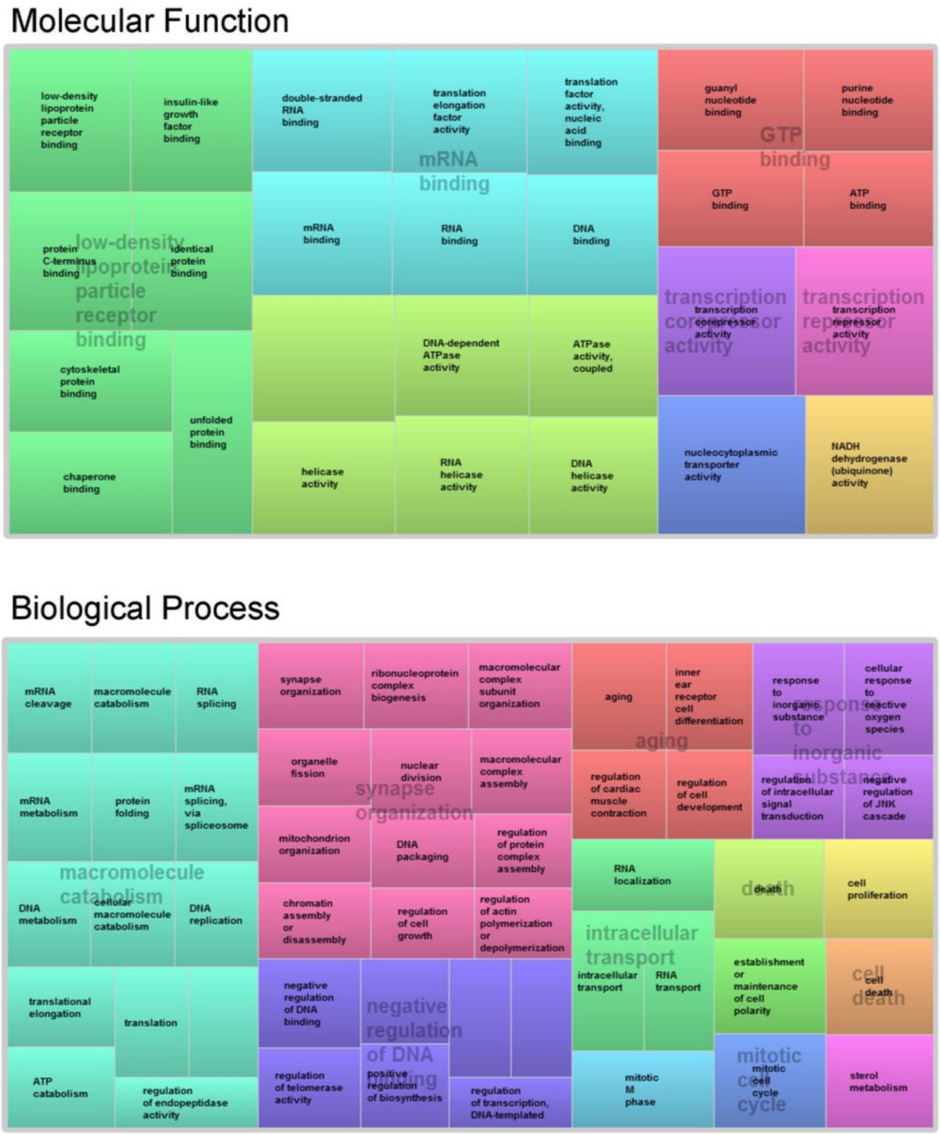

Figure S5. Gene Ontology terms grouped by REVIGO. iPSC-differentiated motor neurons from motor nerve fibroblasts of sporadic ALS and non ALS subjects were submitted to microarray analyses. Differentially expressed genes based on gene ontology (GO) and grouped by REVIGO evidenced molecular functions (mRNA binding, GTP binding, transcription (co)-repressor activities and low-density lipoprotein particle receptor binding) and biological processes (macromolecule catabolism, synapse organization, aging, intracellular transport, negative regulation of DNA binding, death, cell death, mitotic cell cycle and response to inorganic substance) clusters of deregulated genes.

Supplementary Video. Cardiomyocyte differentiation. The video shows spontaneously contracting syncytium of hiPSC-differentiated cardiomyocytes from fibroblasts obtained from the extensor halluces brevis nerve of a sporadic ALS patient. Magnification: 40X.

Table S1. List of all containing differentially expressed genes (p<0.05) in hiPSC-derived motor neurons from sporadic ALS patients compared to non-ALS subjects obtained from microarray analyses.

Probe Name	Gene Symbol	Gene Name	Fold Change	P value
A_23_P139704	*DUSP6*	dual specificity phosphatase 6	-57.42	0.000522
A_33_P3271990	*DGAT1*	diacylglycerol O-acyltransferase 1	-32.83	0.031143
A_33_P3256510	*KCNK12*	potassium channel, subfamily K, member 12	-23.78	0.045734
A_19_P00810156	*KIFC1*	kinesin family member C1	-22.95	0.044022
A_23_P4400	*KRTAP4-11*	keratin associated protein 4-11	-22.95	0.006583
A_19_P00809119	*CASC15*	cancer susceptibility candidate 15 (non-protein coding)	-22.61	0.041169
A_33_P3405022	*LETM1*	leucine zipper-EF-hand containing transmembrane protein 1	-21.61	0.046630
A_24_P117942	*TOMM20L*	translocase of outer mitochondrial membrane 20 homolog (yeast)-like	-20.42	0.000854
A_23_P32165	*LHX2*	LIM homeobox 2	-19.81	0.031360
A_33_P3324383	*FIGNL2*	fidgetin-like 2	-19.49	0.042618
A_33_P3233150	*ZSWIM4*	zinc finger, SWIM-type containing 4	-19.30	0.000769
A_33_P3329088	*PRSS8*	protease, serine, 8	-19.25	0.011072
A_33_P3292840	*C11orf49*	chromosome 11 open reading frame 49	-18.64	0.021540
A_33_P3302255	*ITM2B*	integral membrane protein 2B	-18.16	0.000082
A_24_P8371	*SPNS2*	spinster homolog 2 (Drosophila)	-17.76	0.000002
A_33_P3322373	*GBGT1*	globoside alpha-1,3-N-acetylgalactosaminyltransferase 1	-17.37	0.045698
A_32_P43050	*FRG1*	FSHD region gene 1	-17.24	0.000002
A_23_P163306	*CGNL1*	cingulin-like 1	-16.69	0.020880
A_33_P3251703	*CRIP1*	cysteine-rich protein 1 (intestinal)	-16.40	0.037538
A_33_P3307363	*LPHN2*	latrophilin 2	-16.38	0.003262
A_23_P8452	*LFNG*	LFNG O-fucosylpeptide 3-beta-N-acetylglucosaminyltransferase	-16.31	0.041847
A_33_P3327479	*ZDHHC3*	zinc finger, DHHC-type containing 3	-15.84	0.011077
A_23_P48585	*SALL2*	spalt-like transcription factor 2	-15.76	0.016200
A_33_P3220911	*BST2*	bone marrow stromal cell antigen 2	-15.64	0.000387
A_23_P202520	*ABLIM1*	actin binding LIM protein 1	-15.57	0.000771
A_33_P3424861	*FAM118A*	family with sequence similarity 118, member A	-15.38	0.013017
A_32_P47754	*SLC2A14*	solute carrier family 2 (facilitated glucose transporter), member 14	-15.28	0.047948
A_33_P3230264	*GPC3*	glypican 3	-14.28	0.015214
A_33_P3223592	*APOE*	apolipoprotein E	-14.13	0.012743
A_32_P9842	*MTMR14*	myotubularin related protein 14	-13.96	0.001135
A_23_P215060	*PODXL*	podocalyxin-like	-13.77	0.007311
A_33_P3219256	*BMPR1A*	bone morphogenetic protein receptor, type IA	-13.52	0.043647
A_33_P3260322	*NR6A1*	nuclear receptor subfamily 6, group A, member 1	-13.17	0.033977
A_33_P3319041	*HMGB3*	high mobility group box 3	-12.81	0.015086
A_23_P250212	*SGK223*	homolog of rat pragma of Rnd2	-12.64	0.002689
A_33_P3278941	*REC8*	REC8 meiotic recombination protein	-12.47	0.041208
A_33_P3324495	*ZFP41*	ZFP41 zinc finger protein	-12.38	0.001106
A_23_P166686	*AMOTL2*	angiomotin like 2	-12.17	0.034488
A_23_P63379	*CA14*	carbonic anhydrase XIV	-11.86	0.030162
A_33_P3421748	*SON*	SON DNA binding protein	-11.70	0.005404

A_23_P52727	*NAV2*	neuron navigator 2	-11.39	0.043724
A_33_P3262789	*REEP6*	receptor accessory protein 6	-11.25	0.000217
A_33_P3230698	*KIF1A*	kinesin family member 1A	-11.12	0.000753
A_24_P104512	*EVPL*	envoplakin	-11.02	0.001795
A_33_P3352557	*DMRTA2*	DMRT-like family A2	-10.88	0.043898
A_33_P3325275	*NRSN2*	neurensin 2	-10.73	0.000204
A_33_P3326553	*TMEM82*	transmembrane protein 82	-10.26	0.000170
A_33_P3220698	*EPS8L1*	EPS8-like 1	-10.21	0.002030
A_33_P3414122	*ZNF260*	zinc finger protein 260	-10.20	0.009067
A_23_P216501	**TPM2*	tropomyosin 2 (beta)	-9.60	0.019243
A_33_P3376971	*CHAC1*	ChaC, cation transport regulator homolog 1 (E. coli)	-9.54	0.043647
A_33_P3407042	*B3GALT6*	UDP-Gal:betaGal beta 1,3-galactosyltransferase polypeptide 6	-9.46	0.004707
A_23_P164623	*ZBTB45*	zinc finger and BTB domain containing 45	-9.26	0.000203
A_33_P3387861	*CENPN*	centromere protein N	-9.22	0.005247
A_33_P3243439	*GPR162*	G protein-coupled receptor 162	-8.90	0.031156
A_23_P98580	*FADS2*	fatty acid desaturase 2	-8.90	0.036879
A_24_P149124	*NREP*	neuronal regeneration related protein	-8.86	0.040528
A_33_P3392325	*CDC16*	cell division cycle 16	-8.78	0.037349
A_33_P3390032	**EXOC7*	exocyst complex component 7	-8.73	0.022212
A_23_P214876	*JARID2*	jumonji, AT rich interactive domain 2	-8.68	0.032532
A_33_P3255209	*MGAT5*	mannosyl (alpha-1,6-)-glycoprotein beta-1,6-N-acetyl-glucosaminyltransferase	-8.55	0.000011
A_23_P60499	*ZNF462*	zinc finger protein 462	-8.45	0.018006
A_33_P3349637	*PCDH1*	protocadherin 1	-8.32	0.007901
A_33_P3302312	**IER5L*	immediate early response 5-like	-8.27	0.048587
A_33_P3291294	*EXTL3*	exostosin-like glycosyltransferase 3	-8.23	0.007971
A_33_P3296682	*NDUFB5*	NADH dehydrogenase (ubiquinone) 1 beta subcomplex, 5, 16kDa	-8.22	0.000129
A_33_P3244283	*CAMK2N2*	calcium/calmodulin-dependent protein kinase II inhibitor 2	-8.21	0.000737
A_19_P00329806	*HOTAIR*	HOX transcript antisense RNA	-8.08	0.000125
A_23_P163647	*ECI1*	enoyl-CoA delta isomerase 1	-8.03	0.003713
A_19_P00318396	*HLA-L*	major histocompatibility complex, class I, L (pseudogene)	-7.87	0.013458
A_33_P3219720	*ZNF248*	zinc finger protein 248	-7.76	0.014682
A_23_P87011	*TAGLN*	transgelin	-7.67	0.021991
A_23_P133596	*DROSHA*	drosha, ribonuclease type III	-7.67	0.000019
A_33_P3373358	*GJC1*	gap junction protein, gamma 1, 45kDa	-7.63	0.014595
A_33_P3316323	*RBMS1*	RNA binding motif, single stranded interacting protein 1	-7.63	0.028507
A_33_P3297245	*RRAS2*	related RAS viral (r-ras) oncogene homolog 2	-7.56	0.040594
A_33_P3241753	*NDUFA12*	NADH dehydrogenase (ubiquinone) 1 alpha subcomplex, 12	-7.55	0.000047
A_24_P126181	*NR2C2AP*	nuclear receptor 2C2-associated protein	-7.44	0.021463
A_23_P141893	*PPM1N*	protein phosphatase, Mg2+/Mn2+ dependent, 1N (putative)	-7.29	0.008925
A_23_P355536	*USP54*	ubiquitin specific peptidase 54	-7.24	0.007499
A_33_P3410724	*SDHC*	succinate dehydrogenase complex, subunit C, integral membrane protein, 15kDa	-7.24	0.000044
A_23_P215051	*ECHDC1*	enoyl CoA hydratase domain containing 1	-7.22	0.041035
A_33_P3619819	*KCTD21-AS1*	KCTD21 antisense RNA 1	-7.22	0.036049

A_24_P18802	*VPS18*	vacuolar protein sorting 18 homolog (S. cerevisiae)	-7.21	0.046763
A_23_P215669	*POLR2J2*	polymerase (RNA) II (DNA directed) polypeptide J2	-7.19	0.043478
A_33_P3350828	*EPS15L1*	epidermal growth factor receptor pathway substrate 15-like 1	-7.18	0.020312
A_33_P3297205	*C22orf29*	chromosome 22 open reading frame 29	-7.11	0.000128
A_23_P52986	**VWCE*	von Willebrand factor C and EGF domains	-7.09	0.022841
A_33_P3358799	*SLC35C2*	solute carrier family 35 (GDP-fucose transporter), member C2	-7.02	0.000045
A_24_P664995	*CBX5*	chromobox homolog 5	-6.97	0.047191
A_23_P55948	*PRR12*	proline rich 12	-6.97	0.022876
A_32_P234184	*HES5*	hes family bHLH transcription factor 5	-6.96	0.035273
A_23_P107795	*ATP5SL*	ATP5S-like	-6.93	0.026863
A_33_P3248794	*NAB2*	NGFI-A binding protein 2 (EGR1 binding protein 2)	-6.86	0.002757
A_23_P142310	*MKNK2*	MAP kinase interacting serine/threonine kinase 2	-6.83	0.027141
A_33_P3361027	*TFIP11*	tuftelin interacting protein 11	-6.80	0.008251
A_23_P117582	*JDP2*	Jun dimerization protein 2	-6.79	0.049670
A_23_P77000	*VASH1*	vasohibin 1	-6.77	0.002244
A_23_P53646	*NAP1L1*	nucleosome assembly protein 1-like 1	-6.71	0.047933
A_33_P3402474	*ATAT1*	alpha tubulin acetyltransferase 1	-6.63	0.000156
A_24_P45005	*NPEPL1*	aminopeptidase-like 1	-6.61	0.007941
A_33_P3629678	*COL5A1*	collagen, type V, alpha 1	-6.59	0.000379
A_24_P686965	*SH2D5*	SH2 domain containing 5	-6.58	0.001422
A_33_P3217834	*SMARCAL1*	SWI/SNF related, matrix associated, actin dependent regulator of chromatin, subfamily a-like 1	-6.56	0.005006
A_24_P378019	*IRF7*	interferon regulatory factor 7	-6.55	0.015064
A_23_P39955	*ACTG2*	actin, gamma 2, smooth muscle, enteric	-6.54	0.049786
A_24_P295543	*BLOC1S2*	biogenesis of lysosomal organelles complex-1, subunit 2	-6.53	0.023434
A_23_P34956	*MRPL9*	mitochondrial ribosomal protein L9	-6.50	0.010888
A_33_P3275801	*DES*	desmin	-6.49	0.035772
A_23_P207811	*PAIP1*	poly(A) binding protein interacting protein 1	-6.48	0.047427
A_33_P3286724	*PLD2*	phospholipase D2	-6.42	0.010285
A_24_P50458	*TERF1*	telomeric repeat binding factor (NIMA-interacting) 1	-6.37	0.039925
A_32_P101301	*USP39*	ubiquitin specific peptidase 39	-6.35	0.009295
A_33_P3356502	*RALB*	v-ral simian leukemia viral oncogene homolog B	-6.30	0.013545
A_24_P280901	*RPL21*	ribosomal protein L21	-6.30	0.008809
A_33_P3378835	*SLC9A3R1*	solute carrier family 9, subfamily A (NHE3, cation proton antiporter 3), member 3 regulator 1	-6.27	0.016675
A_33_P3317198	*GTF2F2*	general transcription factor IIF, polypeptide 2, 30kDa	-6.23	0.016988
A_23_P326170	*CALM2*	calmodulin 2 (phosphorylase kinase, delta)	-6.19	0.040629
A_23_P160787	*PEX14*	peroxisomal biogenesis factor 14	-6.09	0.004789
A_33_P3412353	*ZNF268*	zinc finger protein 268	-6.08	0.020791
A_33_P3349947	*TCP1*	t-complex 1	-6.06	0.004641
A_23_P62932	*ATP1B1*	ATPase, Na+/K+ transporting, beta 1 polypeptide	-6.06	0.001795
A_33_P3263387	*TTLL4*	tubulin tyrosine ligase-like family, member 4	-6.02	0.040693
A_33_P3366903	*CHST6*	carbohydrate (N-acetylglucosamine 6-O) sulfotransferase 6	-6.00	0.036849
A_23_P141484	*FAM222B*	family with sequence similarity 222, member B	-5.99	0.018236
A_33_P3376007	*TXLNA*	taxilin alpha	-5.97	0.037973
A_23_P51679	*MEF2D*	myocyte enhancer factor 2D	-5.96	0.000043

A_23_P74449	*HPDL*	4-hydroxyphenylpyruvate dioxygenase-like	-5.95	0.039406
A_33_P3218649	*GPM6B*	glycoprotein M6B	-5.93	0.044536
A_23_P40295	*LAMP5*	lysosomal-associated membrane protein family, member 5	-5.90	0.012238
A_33_P3294881	**CCDC174*	coiled-coil domain containing 174	-5.88	0.041986
A_33_P3313055	*NOTCH3*	notch 3	-5.88	0.006440
A_23_P102320	*NUP35*	nucleoporin 35kDa	-5.86	0.048034
A_23_P256059	*FLJ43681*	ribosomal protein L23a pseudogene	-5.80	0.024667
A_23_P6762	*JAGN1*	jagunal homolog 1 (Drosophila)	-5.80	0.003442
A_33_P3277614	*OLA1*	Obg-like ATPase 1	-5.80	0.007067
A_23_P94319	*KBTBD11*	kelch repeat and BTB (POZ) domain containing 11	-5.79	0.007575
A_33_P3287631	*CTSB*	cathepsin B	-5.78	0.007943
A_24_P244356	*NLRX1*	NLR family member X1	-5.77	0.001832
A_23_P129829	*ORMDL3*	ORMDL sphingolipid biosynthesis regulator 3	-5.75	0.021771
A_24_P131580	*ALPPL2*	alkaline phosphatase, placental-like 2	-5.74	0.001966
A_33_P3266744	*SYTL1*	synaptotagmin-like 1	-5.73	0.007138
A_23_P69877	*ZFP62*	ZFP62 zinc finger protein	-5.70	0.000646
A_23_P358542	*KIFC2*	kinesin family member C2	-5.70	0.045873
A_23_P380614	*ATP9A*	ATPase, class II, type 9A	-5.70	0.034044
A_24_P159837	*ZNF302*	zinc finger protein 302	-5.63	0.040297
A_23_P200551	*USP48*	ubiquitin specific peptidase 48	-5.62	0.000061
A_23_P253221	*ARHGEF4*	Rho guanine nucleotide exchange factor (GEF) 4	-5.58	0.001563
A_33_P3273534	*KRT81*	keratin 81	-5.56	0.001404
A_33_P3339103	*POLR1C*	polymerase (RNA) I polypeptide C, 30kDa	-5.55	0.007600
A_33_P3301025	*CPOX*	coproporphyrinogen oxidase	-5.53	0.017542
A_33_P3289426	*ZNF775*	zinc finger protein 775	-5.50	0.033254
A_23_P17512	*DTD1*	D-tyrosyl-tRNA deacylase 1	-5.48	0.000603
A_23_P166536	*BRD1*	bromodomain containing 1	-5.47	0.014317
A_23_P4572	*MYL12A*	myosin, light chain 12A, regulatory, non-sarcomeric	-5.46	0.047378
A_33_P3381771	*GID4*	GID complex subunit 4	-5.43	0.022212
A_33_P3296308	*DCAF16*	DDB1 and CUL4 associated factor 16	-5.42	0.004383
A_33_P3420655	*KDM4A*	lysine (K)-specific demethylase 4A	-5.40	0.044814
A_23_P108751	*FHL2*	four and a half LIM domains 2	-5.39	0.027286
A_23_P419202	*ZNF658*	zinc finger protein 658	-5.39	0.000651
A_23_P4522	*TXNL1*	thioredoxin-like 1	-5.36	0.036935
A_33_P3290672	**SELT*	selenoprotein T	-5.34	0.001663
A_33_P3318122	*C16orf62*	chromosome 16 open reading frame 62	-5.31	0.002787
A_33_P3210203	*SPIRE2*	spire-type actin nucleation factor 2	-5.30	0.000192
A_33_P3402116	**AGO1*	argonaute RISC catalytic component 1	-5.30	0.022882
A_33_P3217347	*PJA1*	praja ring finger 1, E3 ubiquitin protein ligase	-5.29	0.002450
A_23_P70748	*REPS1*	RALBP1 associated Eps domain containing 1	-5.29	0.000688
A_23_P100220	*ESRP2*	epithelial splicing regulatory protein 2	-5.27	0.039800
A_23_P399001	*CXXC5*	CXXC finger protein 5	-5.27	0.045678
A_23_P37514	*C15orf39*	chromosome 15 open reading frame 39	-5.26	0.006523
A_33_P3265956	**CBFA2T2*	core-binding factor, runt domain, alpha subunit 2; translocated to, 2	-5.25	0.000017

A_33_P3273409	*SLC35E1*	solute carrier family 35, member E1	-5.25	0.000227
A_33_P3320127	*RAB8A*	RAB8A, member RAS oncogene family	-5.25	0.033933
A_33_P3421365	**ZNF169*	zinc finger protein 169	-5.21	0.007317
A_33_P3362631	*GUF1*	GUF1 GTPase homolog (S. cerevisiae)	-5.21	0.008680
A_23_P122906	**AUTS2*	autism susceptibility candidate 2	-5.20	0.027903
A_23_P203420	*MTA2*	metastasis associated 1 family, member 2	-5.20	0.015022
A_33_P3390441	*KPNB1*	karyopherin (importin) beta 1	-5.19	0.015396
A_24_P98914	*PFKM*	phosphofructokinase, muscle	-5.18	0.013618
A_33_P3395743	**VWA1*	von Willebrand factor A domain containing 1	-5.16	0.022393
A_23_P14062	*NUP107*	nucleoporin 107kDa	-5.06	0.020287
A_23_P81392	*WWC1*	WW and C2 domain containing 1	-5.05	0.035120
A_33_P3289296	*TMEM37*	transmembrane protein 37	-5.04	0.046970
A_33_P3781394	*SCAMP1-AS1*	SCAMP1 antisense RNA 1	-5.00	0.000030
A_33_P3315410	*COIL*	coilin	-5.00	0.034517
A_24_P194714	*UBALD2*	UBA-like domain containing 2	-4.97	0.028199
A_24_P296568	*CBX1*	chromobox homolog 1	-4.97	0.000624
A_23_P81212	*MRPS18C*	mitochondrial ribosomal protein S18C	-4.93	0.000079
A_24_P69439	*SLC25A32*	solute carrier family 25 (mitochondrial folate carrier), member 32	-4.92	0.000665
A_24_P873764	*BCR*	breakpoint cluster region	-4.91	0.017624
A_33_P3230688	*CDK10*	cyclin-dependent kinase 10	-4.91	0.010364
A_24_P113131	*BZRAP1*	benzodiazepine receptor (peripheral) associated protein 1	-4.89	0.001177
A_33_P3359543	*ZNF561*	zinc finger protein 561	-4.86	0.006967
A_23_P40194	*DDX27*	DEAD (Asp-Glu-Ala-Asp) box polypeptide 27	-4.83	0.013847
A_24_P296808	*PNMAL1*	paraneoplastic Ma antigen family-like 1	-4.82	0.003057
A_33_P3343295	*CORO7*	coronin 7	-4.81	0.000247
A_23_P215491	*CCL24*	chemokine (C-C motif) ligand 24	-4.79	0.019076
A_33_P3252794	*GLI4*	GLI family zinc finger 4	-4.78	0.011438
A_23_P130149	*ENO3*	enolase 3 (beta, muscle)	-4.77	0.007320
A_33_P3345354	*RRNAD1*	ribosomal RNA adenine dimethylase domain containing 1	-4.76	0.012342
A_23_P121182	*HACL1*	2-hydroxyacyl-CoA lyase 1	-4.75	0.030911
A_33_P3230658	*TSNAX*	translin-associated factor X	-4.74	0.000387
A_33_P3278435	*COA4*	cytochrome c oxidase assembly factor 4 homolog (S. cerevisiae)	-4.74	0.044301
A_33_P3288859	*CDPF1*	cysteine-rich, DPF motif domain containing 1	-4.73	0.026889
A_33_P3385101	*TOLLIP*	toll interacting protein	-4.72	0.000826
A_24_P941336	*TSR1*	TSR1, 20S rRNA accumulation, homolog (S. cerevisiae)	-4.72	0.001765
A_33_P3387771	*USP9X*	ubiquitin specific peptidase 9, X-linked	-4.72	0.010165
A_23_P301138	*IP6K2*	inositol hexakisphosphate kinase 2	-4.70	0.004848
A_33_P3284646	*FBXL6*	F-box and leucine-rich repeat protein 6	-4.70	0.035945
A_33_P3399373	*TPRA1*	transmembrane protein, adipocyte asscociated 1	-4.69	0.009732
A_24_P412976	*TMEM143*	transmembrane protein 143	-4.69	0.000362
A_23_P334709	*FKBP9*	FK506 binding protein 9, 63 kDa	-4.67	0.000323
A_23_P53217	*EED*	embryonic ectoderm development	-4.66	0.001219
A_32_P99100	*PTPRK*	protein tyrosine phosphatase, receptor type, K	-4.65	0.027525
A_23_P150255	**RBM14*	RNA binding motif protein 14	-4.61	0.001980

A_33_P3326733	*GOLGA2P6*	golgin A2 pseudogene 6	-4.60	0.001396
A_33_P3275199	*JRK*	Jrk homolog (mouse)	-4.60	0.027029
A_24_P219552	*NFE2L1*	nuclear factor, erythroid 2-like 1	-4.59	0.013981
A_33_P3397568	*MEMO1*	mediator of cell motility 1	-4.58	0.034424
A_33_P3380567	*SHARPIN*	SHANK-associated RH domain interactor	-4.58	0.045466
A_33_P3338559	**RBBP4*	retinoblastoma binding protein 4	-4.55	0.023963
A_33_P3253234	*IQSEC2*	IQ motif and Sec7 domain 2	-4.54	0.005706
A_33_P3329344	*FASN*	fatty acid synthase	-4.54	0.016945
A_23_P132405	*ACAD9*	acyl-CoA dehydrogenase family, member 9	-4.54	0.028158
A_33_P3367830	*EFEMP2*	EGF containing fibulin-like extracellular matrix protein 2	-4.53	0.002607
A_24_P245246	*PIP4K2B*	phosphatidylinositol-5-phosphate 4-kinase, type II, beta	-4.53	0.013717
A_23_P132784	*FXR1*	fragile X mental retardation, autosomal homolog 1	-4.52	0.011961
A_24_P98411	*HSPA5*	heat shock 70kDa protein 5 (glucose-regulated protein, 78kDa)	-4.49	0.008560
A_23_P217938	*SPHAR*	S-phase response (cyclin related)	-4.46	0.003777
A_33_P3317850	*RIOK3*	RIO kinase 3	-4.46	0.003680
A_23_P5912	*YTHDF1*	YTH domain family, member 1	-4.45	0.005197
A_23_P259741	*SATB1*	SATB homeobox 1	-4.45	0.048886
A_33_P3213752	*CDK5RAP1*	CDK5 regulatory subunit associated protein 1	-4.44	0.021545
A_33_P3279708	*RNU2-1*	RNA, U2 small nuclear 1	-4.42	0.002708
A_33_P3382560	*RPL23A*	ribosomal protein L23a	-4.41	0.023552
A_23_P201279	*UBE4B*	ubiquitination factor E4B	-4.41	0.039980
A_23_P76538	*TESC*	tescalcin	-4.41	0.013767
A_23_P398275	*GOLGA2P7*	golgin A2 pseudogene 7	-4.41	0.020108
A_33_P3328426	*ANO10*	anoctamin 10	-4.38	0.044137
A_33_P3357322	*SMC2*	structural maintenance of chromosomes 2	-4.37	0.032471
A_33_P3386467	*RPL23AP7*	ribosomal protein L23a pseudogene 7	-4.37	0.000397
A_33_P3379726	*CCDC106*	coiled-coil domain containing 106	-4.36	0.032428
A_23_P41734	*RNF130*	ring finger protein 130	-4.35	0.000340
A_23_P88753	*TSR3*	TSR3, 20S rRNA accumulation, homolog (S. cerevisiae)	-4.33	0.008750
A_23_P74115	*RAD54L*	RAD54-like (S. cerevisiae)	-4.33	0.005280
A_33_P3245066	*SLC35E2B*	solute carrier family 35, member E2B	-4.31	0.002755
A_33_P3238166	*PXDN*	peroxidasin homolog (Drosophila)	-4.29	0.017904
A_23_P133923	*GPANK1*	G patch domain and ankyrin repeats 1	-4.28	0.019291
A_23_P345674	*ZNF71*	zinc finger protein 71	-4.28	0.037042
A_23_P101960	*ZFP36L2*	ZFP36 ring finger protein-like 2	-4.28	0.028429
A_24_P234116	*C1orf56*	chromosome 1 open reading frame 56	-4.25	0.047638
A_33_P3295283	*LPCAT3*	lysophosphatidylcholine acyltransferase 3	-4.24	0.006235
A_23_P152984	*ALYREF*	Aly/REF export factor	-4.24	0.004967
A_23_P405531	*WIZ*	widely interspaced zinc finger motifs	-4.24	0.021531
A_33_P3372580	*COG1*	component of oligomeric golgi complex 1	-4.24	0.029086
A_23_P400147	*CCDC61*	coiled-coil domain containing 61	-4.22	0.001969
A_23_P200222	*LRP8*	low density lipoprotein receptor-related protein 8, apolipoprotein e receptor	-4.22	0.045763
A_33_P3255499	*SLC25A51*	solute carrier family 25, member 51	-4.18	0.037085
A_19_P00806490	*TET1*	tet methylcytosine dioxygenase 1	-4.18	0.040834

A_33_P3220837	*MAFB*	v-maf avian musculoaponeurotic fibrosarcoma oncogene homolog B	-4.18	0.018291
A_23_P54376	*STOML1*	stomatin (EPB72)-like 1	-4.17	0.011458
A_33_P3299854	*NCBP1*	nuclear cap binding protein subunit 1, 80kDa	-4.17	0.045446
A_19_P00800241	*TPGS2*	tubulin polyglutamylase complex subunit 2	-4.17	0.010757
A_24_P246351	*FAM71E1*	family with sequence similarity 71, member E1	-4.17	0.044441
A_33_P3268567	*NCK2*	NCK adaptor protein 2	-4.16	0.015970
A_33_P3601163	*ILF3-AS1*	ILF3 antisense RNA 1 (head to head)	-4.15	0.015832
A_33_P3269203	*SERPINH1*	serpin peptidase inhibitor, clade H (heat shock protein 47), member 1, (collagen binding protein 1)	-4.15	0.021790
A_23_P303181	*SPHK2*	sphingosine kinase 2	-4.15	0.015786
A_23_P30813	*HIST1H4K*	histone cluster 1, H4k	-4.15	0.049089
A_23_P385861	*CDCA2*	cell division cycle associated 2	-4.14	0.001527
A_33_P3263061	*AK3*	adenylate kinase 3	-4.13	0.024240
A_33_P3387696	*TMBIM4*	transmembrane BAX inhibitor motif containing 4	-4.11	0.037817
A_23_P312174	*ALMS1*	Alstrom syndrome 1	-4.11	0.045222
A_23_P118462	*OVCA2*	ovarian tumor suppressor candidate 2	-4.10	0.003660
A_24_P232696	*SMARCD1*	SWI/SNF related, matrix associated, actin dependent regulator of chromatin, subfamily d, member 1	-4.08	0.009140
A_33_P3262575	*BAIAP2L1*	BAI1-associated protein 2-like 1	-4.08	0.026195
A_23_P57667	*PLXNA1*	plexin A1	-4.08	0.023471
A_23_P257911	*USP16*	ubiquitin specific peptidase 16	-4.06	0.001667
A_23_P355824	*MGRN1*	mahogunin ring finger 1, E3 ubiquitin protein ligase	-4.06	0.048720
A_33_P3295550	*TET1*	tet methylcytosine dioxygenase 1	-4.05	0.004983
A_33_P3272593	*CTPS2*	CTP synthase 2	-4.03	0.018906
A_33_P3284463	*ASAH1*	N-acylsphingosine amidohydrolase (acid ceramidase) 1	-4.03	0.047649
A_23_P501435	*CSRP2BP*	CSRP2 binding protein	-4.02	0.027967
A_23_P12363	*ROR1*	receptor tyrosine kinase-like orphan receptor 1	-4.01	0.049232
A_33_P3210338	*RFX7*	regulatory factor X, 7	-4.00	0.045307
A_33_P3221748	*RUNX3*	runt-related transcription factor 3	-3.98	0.000037
A_23_P62731	*MRPS14*	mitochondrial ribosomal protein S14	-3.97	0.006109
A_33_P3413053	*NUBP1*	nucleotide binding protein 1	-3.97	0.034597
A_33_P3358740	*OSBPL7*	oxysterol binding protein-like 7	-3.97	0.027934
A_33_P3257279	*TMEM145*	transmembrane protein 145	-3.97	0.000117
A_23_P154605	*SULF2*	sulfatase 2	-3.96	0.029936
A_24_P85158	*SIRT3*	sirtuin 3	-3.95	0.013792
A_23_P27315	*EMILIN2*	elastin microfibril interfacer 2	-3.94	0.001481
A_33_P3308914	*CIB2*	calcium and integrin binding family member 2	-3.92	0.005586
A_23_P120414	*YWHAB*	tyrosine 3-monooxygenase/tryptophan 5-monooxygenase activation protein, beta	-3.92	0.030951
A_33_P3237574	*HLA-A*	major histocompatibility complex, class I, A	-3.92	0.008095
A_23_P115573	*SHISA4*	shisa family member 4	-3.92	0.000475
A_24_P177964	*NCKAP5L*	NCK-associated protein 5-like	-3.91	0.003013
A_32_P101031	*LYPD1*	LY6/PLAUR domain containing 1	-3.90	0.039273
A_33_P3372099	*DDIT4L*	DNA-damage-inducible transcript 4-like	-3.90	0.007563
A_23_P165494	*GORASP2*	golgi reassembly stacking protein 2, 55kDa	-3.90	0.013891
A_33_P3270034	*NCOA4*	nuclear receptor coactivator 4	-3.89	0.034132

A_23_P72138	*MRPS22*	mitochondrial ribosomal protein S22	-3.87	0.024971
A_33_P3329839	*CDK2AP2*	cyclin-dependent kinase 2 associated protein 2	-3.87	0.032253
A_23_P24926	*FNTA*	farnesyltransferase, CAAX box, alpha	-3.87	0.000883
A_23_P111621	*GTF2IRD1*	GTF2I repeat domain containing 1	-3.86	0.041177
A_23_P83436	*PEPD*	peptidase D	-3.85	0.001330
A_23_P397376	*MAF*	v-maf avian musculoaponeurotic fibrosarcoma oncogene homolog	-3.85	0.005942
A_33_P3231750	*ZNF738*	zinc finger protein 738	-3.84	0.035723
A_33_P3335386	**FAM83G*	family with sequence similarity 83, member G	-3.83	0.021951
A_23_P385034	*E2F3*	E2F transcription factor 3	-3.82	0.036896
A_23_P158725	*SLC16A3*	solute carrier family 16 (monocarboxylate transporter), member 3	-3.81	0.022295
A_23_P17870	*TMEM184B*	transmembrane protein 184B	-3.81	0.035419
A_24_P89911	*NAIF1*	nuclear apoptosis inducing factor 1	-3.81	0.047960
A_23_P166716	*TRMT10C*	tRNA methyltransferase 10 homolog C (S. cerevisiae)	-3.81	0.027570
A_23_P371865	*CDYL2*	chromodomain protein, Y-like 2	-3.80	0.040053
A_33_P3290729	*POU3F2*	POU class 3 homeobox 2	-3.79	0.007228
A_33_P3416350	*ITSN2*	intersectin 2	-3.79	0.016478
A_33_P3329255	*NDST2*	N-deacetylase/N-sulfotransferase (heparan glucosaminyl) 2	-3.78	0.028349
A_33_P3362668	*LOC100132352*	FSHD region gene 1 pseudogene	-3.78	0.000573
A_19_P00315843	*SCARNA16*	small Cajal body-specific RNA 16	-3.78	0.025788
A_33_P3297444	*ABTB1*	ankyrin repeat and BTB (POZ) domain containing 1	-3.77	0.043419
A_23_P100868	*MYO19*	myosin XIX	-3.76	0.046512
A_33_P3257714	*RPS23*	ribosomal protein S23	-3.76	0.000168
A_23_P360964	*DACT3*	dishevelled-binding antagonist of beta-catenin 3	-3.76	0.005696
A_23_P138856	*DRAP1*	DR1-associated protein 1 (negative cofactor 2 alpha)	-3.74	0.025601
A_33_P3387463	*ATP6V1D*	ATPase, H+ transporting, lysosomal 34kDa, V1 subunit D	-3.73	0.008338
A_33_P3375383	*SNAPC2*	small nuclear RNA activating complex, polypeptide 2, 45kDa	-3.71	0.040815
A_33_P3265290	*RPL24*	ribosomal protein L24	-3.70	0.029889
A_33_P3333587	*FBXL20*	F-box and leucine-rich repeat protein 20	-3.70	0.027476
A_33_P3369550	*SRSF11*	serine/arginine-rich splicing factor 11	-3.70	0.025947
A_33_P3235925	*PRKRIP1*	PRKR interacting protein 1 (IL11 inducible)	-3.69	0.001328
A_33_P3853081	*ALDOAP2*	aldolase A, fructose-bisphosphate pseudogene 2	-3.68	0.027120
A_24_P14010	*NMT1*	N-myristoyltransferase 1	-3.68	0.030953
A_24_P926367	*THRAP3*	thyroid hormone receptor associated protein 3	-3.67	0.012726
A_33_P3397795	*PCNXL4*	pecanex-like 4 (Drosophila)	-3.65	0.045071
A_23_P38365	*TLK2*	tousled-like kinase 2	-3.65	0.004738
A_33_P3406939	*KIF24*	kinesin family member 24	-3.64	0.031590
A_33_P3264121	*TBCA*	tubulin folding cofactor A	-3.63	0.047007
A_33_P3310475	*41893*	septin 11	-3.62	0.029340
A_23_P169178	*TESK1*	testis-specific kinase 1	-3.60	0.017670
A_23_P91491	*C21orf59*	chromosome 21 open reading frame 59	-3.60	0.011364
A_33_P3799936	*ARHGEF10L*	Rho guanine nucleotide exchange factor (GEF) 10-like	-3.60	0.047314
A_33_P3355717	*ZFP36L1*	ZFP36 ring finger protein-like 1	-3.59	0.001443
A_33_P3344308	*APTX*	aprataxin	-3.58	0.011673
A_33_P3499102	*RBM10*	RNA binding motif protein 10	-3.58	0.041954

A_24_P239176	*MUC4*	mucin 4, cell surface associated	-3.58	0.027067
A_23_P133694	*SLC29A1*	solute carrier family 29 (equilibrative nucleoside transporter), member 1	-3.56	0.002066
A_23_P76749	*GALNT16*	polypeptide N-acetylgalactosaminyltransferase 16	-3.55	0.033832
A_33_P3358099	**CD300E*	CD300e molecule	-3.55	0.048299
A_23_P202170	*MGEA5*	meningioma expressed antigen 5 (hyaluronidase)	-3.55	0.001999
A_33_P3360216	*HIST1H2AI*	histone cluster 1, H2ai	-3.54	0.001743
A_23_P88484	*DUT*	deoxyuridine triphosphatase	-3.54	0.038048
A_23_P14649	*ANP32A-IT1*	ANP32A intronic transcript 1 (non-protein coding)	-3.54	0.039281
A_23_P92261	*ECE2*	endothelin converting enzyme 2	-3.53	0.048358
A_24_P219378	**CASKIN1*	CASK interacting protein 1	-3.53	0.000019
A_23_P168130	*IP6K3*	inositol hexakisphosphate kinase 3	-3.51	0.010643
A_23_P24535	*TTC12*	tetratricopeptide repeat domain 12	-3.51	0.011153
A_23_P10385	*DTL*	denticleless E3 ubiquitin protein ligase homolog (Drosophila)	-3.50	0.010854
A_23_P11331	*TCEAL8*	transcription elongation factor A (SII)-like 8	-3.50	0.042164
A_33_P3242388	*PIGX*	phosphatidylinositol glycan anchor biosynthesis, class X	-3.50	0.040387
A_33_P3241786	*ADD2*	adducin 2 (beta)	-3.50	0.037617
A_23_P165186	*LIN37*	lin-37 homolog (C. elegans)	-3.50	0.015009
A_33_P3357247	*USP36*	ubiquitin specific peptidase 36	-3.50	0.042267
A_23_P258221	*ABCC5*	ATP-binding cassette, sub-family C (CFTR/MRP), member 5	-3.49	0.022133
A_24_P357037	*UBE2G2*	ubiquitin-conjugating enzyme E2G 2	-3.49	0.031145
A_24_P261724	*RNF10*	ring finger protein 10	-3.47	0.001679
A_23_P360302	*GUCY2F*	guanylate cyclase 2F, retinal	-3.45	0.032286
A_33_P3292179	*ABCA9*	ATP-binding cassette, sub-family A (ABC1), member 9	-3.45	0.022148
A_33_P3333033	**SGSM2*	small G protein signaling modulator 2	-3.44	0.035626
A_23_P208310	*CD3EAP*	CD3e molecule, epsilon associated protein	-3.44	0.039621
A_23_P112652	*CNOT10*	CCR4-NOT transcription complex, subunit 10	-3.43	0.014482
A_33_P3354021	*RPL23AP53*	ribosomal protein L23a pseudogene 53	-3.43	0.005694
A_23_P138025	*GPN2*	GPN-loop GTPase 2	-3.42	0.007070
A_33_P3292235	*PAQR3*	progestin and adipoQ receptor family member III	-3.41	0.032251
A_23_P107735	*CD79A*	CD79a molecule, immunoglobulin-associated alpha	-3.41	0.008845
A_23_P18317	*SLC41A3*	solute carrier family 41, member 3	-3.40	0.029465
A_33_P3252236	*N6AMT1*	N-6 adenine-specific DNA methyltransferase 1 (putative)	-3.40	0.043422
A_23_P163467	*C15orf52*	chromosome 15 open reading frame 52	-3.39	0.003693
A_23_P109026	*KCNK15*	potassium channel, subfamily K, member 15	-3.39	0.003504
A_23_P140427	*EVL*	Enah/Vasp-like	-3.37	0.002691
A_23_P315933	*ABHD11*	abhydrolase domain containing 11	-3.37	0.013792
A_24_P48898	*APOL2*	apolipoprotein L, 2	-3.34	0.022809
A_23_P8981	*STAR*	steroidogenic acute regulatory protein	-3.34	0.019013
A_33_P3221563	*ARMC5*	armadillo repeat containing 5	-3.33	0.012691
A_24_P235266	*GRB10*	growth factor receptor-bound protein 10	-3.33	0.018468
A_32_P104000	*DCUN1D3*	DCN1, defective in cullin neddylation 1, domain containing 3	-3.32	0.044137
A_33_P3357620	*PAGR1*	PAXIP1 associated glutamate-rich protein 1	-3.32	0.046713
A_23_P128372	*FKBP4*	FK506 binding protein 4, 59kDa	-3.32	0.000668
A_33_P3351175	**WNK2*	WNK lysine deficient protein kinase 2	-3.31	0.022347

A_23_P112801	*CHP1*	calcineurin-like EF-hand protein 1	-3.31	0.049422
A_23_P38346	*DHX58*	DEXH (Asp-Glu-X-His) box polypeptide 58	-3.31	0.002614
A_33_P3362153	*TMEM238*	transmembrane protein 238	-3.30	0.015552
A_23_P431853	*ND2*	MTND2	-3.30	0.022316
A_23_P12463	*QSOX1*	quiescin Q6 sulfhydryl oxidase 1	-3.30	0.010407
A_23_P48550	*CEP170B*	centrosomal protein 170B	-3.30	0.002124
A_33_P3287710	*DLG5-AS1*	DLG5 antisense RNA 1	-3.30	0.033876
A_23_P28318	*NDUFAF7*	NADH dehydrogenase (ubiquinone) complex I, assembly factor 7	-3.27	0.047764
A_23_P137909	*HIST3H3*	histone cluster 3, H3	-3.27	0.027758
A_33_P3417626	*ENHO*	energy homeostasis associated	-3.27	0.030814
A_23_P35564	*SEC31B*	SEC31 homolog B (S. cerevisiae)	-3.25	0.034515
A_23_P153086	*RBFA*	ribosome binding factor A (putative)	-3.25	0.041132
A_23_P19987	*IGF2BP3*	insulin-like growth factor 2 mRNA binding protein 3	-3.24	0.017996
A_33_P3327663	*SUSD4*	sushi domain containing 4	-3.23	0.025141
A_33_P3494748	*TMEM65*	transmembrane protein 65	-3.22	0.000434
A_23_P384044	*CNIH3*	cornichon family AMPA receptor auxiliary protein 3	-3.21	0.037794
A_24_P365365	*TCF3*	transcription factor 3	-3.20	0.030945
A_24_P945283	*DLG3*	discs, large homolog 3 (Drosophila)	-3.20	0.000559
A_23_P171249	*IGBP1*	immunoglobulin (CD79A) binding protein 1	-3.19	0.025474
A_23_P26954	*VAT1*	vesicle amine transport 1	-3.16	0.000418
A_23_P310331	**RANBP3*	RAN binding protein 3	-3.16	0.000386
A_33_P3604591	*SNORA78*	small nucleolar RNA, H/ACA box 78	-3.16	0.001464
A_23_P163546	*TAF1C*	TATA box binding protein (TBP)-associated factor, RNA polymerase I, C, 110kDa	-3.15	0.011011
A_33_P3375613	*C8orf44-SGK3*	C8orf44-SGK3 readthrough	-3.15	0.000543
A_33_P3396591	*PCED1A*	PC-esterase domain containing 1A	-3.15	0.022800
A_33_P3227467	*BNIP2*	BCL2/adenovirus E1B 19kDa interacting protein 2	-3.14	0.040216
A_23_P67725	*LMNB2*	lamin B2	-3.14	0.036221
A_33_P3273020	*FKBP2*	FK506 binding protein 2, 13kDa	-3.13	0.031558
A_23_P71644	*FANCG*	Fanconi anemia, complementation group G	-3.13	0.017604
A_23_P487	*UCK2*	uridine-cytidine kinase 2	-3.12	0.001013
A_23_P356565	*RRP8*	ribosomal RNA processing 8, methyltransferase, homolog (yeast)	-3.11	0.001321
A_23_P156809	*METTL21A*	methyltransferase like 21A	-3.11	0.008484
A_24_P391104	*RFX1*	regulatory factor X, 1 (influences HLA class II expression)	-3.11	0.018007
A_23_P211285	*NDUFV3*	NADH dehydrogenase (ubiquinone) flavoprotein 3, 10kDa	-3.11	0.034873
A_33_P3879920	*TRIM39-RPP21*	TRIM39-RPP21 readthrough	-3.10	0.034494
A_33_P3294297	*RNF126*	ring finger protein 126	-3.09	0.009291
A_33_P3301306	*KMT2C*	lysine (K)-specific methyltransferase 2C	-3.09	0.014479
A_23_P115105	*SZRD1*	SUZ RNA binding domain containing 1	-3.09	0.015828
A_33_P3259507	*FBXO10*	F-box protein 10	-3.08	0.041319
A_33_P3619221	*ZMYND8*	zinc finger, MYND-type containing 8	-3.08	0.026065
A_33_P3332135	*PHOSPHO1*	phosphatase, orphan 1	-3.08	0.031073
A_33_P3260500	*BREA2*	breast cancer estrogen-induced apoptosis 2	-3.07	0.032108
A_19_P00805272	*KIAA1671*	KIAA1671	-3.06	0.000311

A_32_P8529	*SNHG20*	small nucleolar RNA host gene 20 (non-protein coding)	-3.06	0.033225
A_33_P3356220	*STARD3*	StAR-related lipid transfer (START) domain containing 3	-3.04	0.007151
A_23_P54000	*SNX6*	sorting nexin 6	-3.03	0.033218
A_33_P3227324	*MIER2*	mesoderm induction early response 1, family member 2	-3.03	0.001579
A_33_P3246613	*CCDC78*	coiled-coil domain containing 78	-3.02	0.002479
A_32_P219520	*TNFAIP8*	tumor necrosis factor, alpha-induced protein 8	-3.02	0.000863
A_23_P33664	*ELSPBP1*	epididymal sperm binding protein 1	-3.02	0.006821
A_23_P11843	**LRRN2*	leucine rich repeat neuronal 2	-3.01	0.011729
A_33_P3336652	*NDUFC2*	NADH dehydrogenase (ubiquinone) 1, subcomplex unknown, 2, 14.5kDa	-3.00	0.001431
A_23_P120153	*RNF149*	ring finger protein 149	-3.00	0.039944
A_23_P89249	*ERBB2*	v-erb-b2 avian erythroblastic leukemia viral oncogene homolog 2	-3.00	0.027084
A_23_P92424	*UTP3*	UTP3, small subunit (SSU) processome component, homolog (S. cerevisiae)	-2.99	0.000062
A_33_P3337318	*HNRNPA2B1*	heterogeneous nuclear ribonucleoprotein A2/B1	-2.99	0.042901
A_23_P80032	*E2F1*	E2F transcription factor 1	-2.97	0.007427
A_23_P85969	*ZNF326*	zinc finger protein 326	-2.97	0.038142
A_23_P62588	*CALML6*	calmodulin-like 6	-2.96	0.023563
A_33_P3216372	*IQGAP2*	IQ motif containing GTPase activating protein 2	-2.96	0.042370
A_23_P141555	*TBX21*	T-box 21	-2.94	0.027428
A_33_P3221064	*LTBP4*	latent transforming growth factor beta binding protein 4	-2.94	0.022544
A_23_P411833	*WDR90*	WD repeat domain 90	-2.93	0.000805
A_23_P92320	*NUP54*	nucleoporin 54kDa	-2.93	0.018600
A_33_P3232508	*SMARCB1*	SWI/SNF related, matrix associated, actin dependent regulator of chromatin, subfamily b, member 1	-2.93	0.032526
A_33_P3379941	*HMGXB3*	HMG box domain containing 3	-2.92	0.013394
A_33_P3214159	*CDH2*	cadherin 2, type 1, N-cadherin (neuronal)	-2.92	0.024414
A_33_P3350488	*NUSAP1*	nucleolar and spindle associated protein 1	-2.89	0.007633
A_33_P3262555	*MEX3D*	mex-3 RNA binding family member D	-2.88	0.008310
A_23_P88680	*EMC7*	ER membrane protein complex subunit 7	-2.88	0.022830
A_19_P00315717	*FAM200B*	family with sequence similarity 200, member B	-2.88	0.004503
A_23_P314070	*ARHGAP1*	Rho GTPase activating protein 1	-2.88	0.009269
A_33_P3422233	*UVSSA*	UV-stimulated scaffold protein A	-2.85	0.004131
A_32_P36235	*IER2*	immediate early response 2	-2.85	0.045233
A_23_P161237	*GBF1*	golgi brefeldin A resistant guanine nucleotide exchange factor 1	-2.85	0.001445
A_33_P3334220	*ACACB*	acetyl-CoA carboxylase beta	-2.85	0.036711
A_33_P3422330	*KLHL17*	kelch-like family member 17	-2.84	0.004660
A_33_P3314550	*RAB3A*	RAB3A, member RAS oncogene family	-2.84	0.009266
A_23_P28590	*FASTKD1*	FAST kinase domains 1	-2.84	0.041730
A_23_P108708	*RNF181*	ring finger protein 181	-2.82	0.000401
A_23_P34233	*QPRT*	quinolinate phosphoribosyltransferase	-2.82	0.000252
A_33_P3236082	*NSDHL*	NAD(P) dependent steroid dehydrogenase-like	-2.82	0.017414
A_33_P3394183	*ZNF324B*	zinc finger protein 324B	-2.81	0.000744
A_33_P3230090	*LUZP6*	leucine zipper protein 6	-2.81	0.001461
A_23_P163117	*C14orf169*	chromosome 14 open reading frame 169	-2.81	0.032486
A_32_P232192	**DIS3L2*	DIS3 like 3'-5' exoribonuclease 2	-2.81	0.002097

A_23_P53276	*TIMELESS*	timeless circadian clock	-2.80	0.009966
A_24_P238333	*NSA2*	NSA2 ribosome biogenesis homolog (S. cerevisiae)	-2.80	0.036604
A_23_P416468	*PIF1*	PIF1 5'-to-3' DNA helicase	-2.79	0.030935
A_23_P7144	*CXCL1*	chemokine (C-X-C motif) ligand 1 (melanoma growth stimulating activity, alpha)	-2.78	0.017304
A_23_P38677	*SLMO1*	slowmo homolog 1 (Drosophila)	-2.78	0.034496
A_33_P3350169	*CCDC178*	coiled-coil domain containing 178	-2.78	0.018738
A_33_P3220643	*PTRH1*	peptidyl-tRNA hydrolase 1 homolog (S. cerevisiae)	-2.78	0.024489
A_23_P112061	*HGSNAT*	heparan-alpha-glucosaminide N-acetyltransferase	-2.77	0.034613
A_24_P161036	*ACOT1*	acyl-CoA thioesterase 1	-2.77	0.012847
A_23_P71591	*NOL8*	nucleolar protein 8	-2.75	0.043817
A_33_P3223056	*ADAMTS10*	ADAM metallopeptidase with thrombospondin type 1 motif, 10	-2.75	0.007525
A_33_P3412658	*KCNIP2-AS1*	KCNIP2 antisense RNA 1	-2.75	0.009250
A_33_P3213064	*STAT2*	signal transducer and activator of transcription 2, 113kDa	-2.75	0.003104
A_24_P181585	*LRRC59*	leucine rich repeat containing 59	-2.73	0.040369
A_33_P3358148	*CC2D2B*	coiled-coil and C2 domain containing 2B	-2.73	0.018179
A_23_P257795	*NDUFA2*	NADH dehydrogenase (ubiquinone) 1 alpha subcomplex, 2, 8kDa	-2.72	0.029025
A_32_P41405	*THOC3*	THO complex 3	-2.72	0.006028
A_33_P3419321	*CHD5*	chromodomain helicase DNA binding protein 5	-2.72	0.040895
A_24_P194154	*UPF1*	UPF1 regulator of nonsense transcripts homolog (yeast)	-2.71	0.048277
A_33_P3284557	**ZAK*	sterile alpha motif and leucine zipper containing kinase AZK	-2.70	0.023591
A_33_P3238335	*MCOLN3*	mucolipin 3	-2.70	0.044596
A_23_P48717	*NPC2*	Niemann-Pick disease, type C2	-2.69	0.048880
A_33_P3602006	*ADAT2*	adenosine deaminase, tRNA-specific 2	-2.69	0.038499
A_33_P3305482	*EIF2B3*	eukaryotic translation initiation factor 2B, subunit 3 gamma, 58kDa	-2.68	0.019395
A_33_P3234899	*PSMB3*	proteasome (prosome, macropain) subunit, beta type, 3	-2.68	0.037820
A_23_P329890	*TMEM136*	transmembrane protein 136	-2.67	0.024858
A_23_P147984	*TRIP12*	thyroid hormone receptor interactor 12	-2.67	0.040714
A_33_P3368706	*NFYC*	nuclear transcription factor Y, gamma	-2.66	0.034772
A_33_P3250845	*NIPA2*	non imprinted in Prader-Willi/Angelman syndrome 2	-2.66	0.023152
A_23_P109821	*TADA3*	transcriptional adaptor 3	-2.66	0.025008
A_19_P00320643	*CA5BP1*	carbonic anhydrase VB pseudogene 1	-2.66	0.006028
A_23_P12189	*DFFA*	DNA fragmentation factor, 45kDa, alpha polypeptide	-2.65	0.010037
A_23_P117683	*HYPK*	huntingtin interacting protein K	-2.65	0.011909
A_32_P234935	*TARDBP*	TAR DNA binding protein	-2.65	0.016906
A_23_P258748	*C2orf83*	chromosome 2 open reading frame 83	-2.65	0.038680
A_24_P218074	*ZNF467*	zinc finger protein 467	-2.64	0.005598
A_23_P92629	*CWC27*	CWC27 spliceosome-associated protein homolog (S. cerevisiae)	-2.64	0.026825
A_33_P3210965	*TCTN1*	tectonic family member 1	-2.63	0.003319
A_23_P211909	*PLS1*	plastin 1	-2.63	0.033110
A_32_P313405	*LAMA1*	laminin, alpha 1	-2.62	0.014171
A_33_P3269844	*LRRC26*	leucine rich repeat containing 26	-2.62	0.011378
A_33_P3571120	*AP3D1*	adaptor-related protein complex 3, delta 1 subunit	-2.62	0.001607
A_33_P3522525	*GRPEL1*	GrpE-like 1, mitochondrial (E. coli)	-2.62	0.011118

A_33_P3256282	*KRTAP10-2*	keratin associated protein 10-2	-2.62	0.036032
A_33_P3323323	*ZNF497*	zinc finger protein 497	-2.61	0.043270
A_33_P3244843	*PRKAG3*	protein kinase, AMP-activated, gamma 3 non-catalytic subunit	-2.61	0.015720
A_24_P175909	*MARS*	methionyl-tRNA synthetase	-2.60	0.038427
A_33_P3288364	*SPATA2L*	spermatogenesis associated 2-like	-2.60	0.014232
A_23_P39766	*GLS*	glutaminase	-2.59	0.007117
A_33_P3341365	*RNF216*	ring finger protein 216	-2.59	0.042252
A_23_P12643	*AS3MT*	arsenite methyltransferase	-2.59	0.026874
A_23_P395418	*PCDHGB3*	protocadherin gamma subfamily B, 3	-2.58	0.037734
A_24_P280113	*IL13RA1*	interleukin 13 receptor, alpha 1	-2.58	0.047154
A_33_P3424800	*HLA-B*	major histocompatibility complex, class I, B	-2.58	0.048912
A_32_P53524	*NTN1*	netrin 1	-2.58	0.034348
A_33_P3307500	*STRA6*	stimulated by retinoic acid 6	-2.57	0.022523
A_24_P302574	*OSGEPL1*	O-sialoglycoprotein endopeptidase-like 1	-2.57	0.036215
A_23_P213518	*CAST*	calpastatin	-2.56	0.006835
A_33_P3387272	**EEF1A1*	eukaryotic translation elongation factor 1 alpha 1	-2.56	0.000242
A_24_P365807	*EFNB1*	ephrin-B1	-2.56	0.025991
A_33_P3229122	*HIST1H2BF*	histone cluster 1, H2bf	-2.56	0.001992
A_23_P89910	*GEMIN7*	gem (nuclear organelle) associated protein 7	-2.54	0.002689
A_23_P121586	*HERC3*	HECT and RLD domain containing E3 ubiquitin protein ligase 3	-2.53	0.018489
A_33_P3397418	*ZC3HAV1*	zinc finger CCCH-type, antiviral 1	-2.52	0.001861
A_33_P3392077	*TP53I3*	tumor protein p53 inducible protein 3	-2.52	0.035974
A_23_P116387	*INCENP*	inner centromere protein antigens 135/155kDa	-2.52	0.030914
A_24_P144377	*DEXI*	Dexi homolog (mouse)	-2.52	0.014114
A_33_P3423506	*NXPE2*	neurexophilin and PC-esterase domain family, member 2	-2.51	0.034537
A_23_P375524	*LCE1D*	late cornified envelope 1D	-2.50	0.015509
A_23_P7361	*ELOVL6*	ELOVL fatty acid elongase 6	-2.50	0.046266
A_33_P3396692	*SSR1*	signal sequence receptor, alpha	-2.50	0.040181
A_24_P96762	*TRNAU1AP*	tRNA selenocysteine 1 associated protein 1	-2.50	0.003432
A_33_P3360773	*TSSC4*	tumor suppressing subtransferable candidate 4	-2.49	0.027891
A_33_P3301524	*XRCC3*	X-ray repair complementing defective repair in Chinese hamster cells 3	-2.49	0.014605
A_33_P3220919	*ADRBK2*	adrenergic, beta, receptor kinase 2	-2.48	0.001073
A_23_P152055	*EFTUD1*	elongation factor Tu GTP binding domain containing 1	-2.48	0.047375
A_23_P256205	*ABLIM3*	actin binding LIM protein family, member 3	-2.48	0.027266
A_33_P3370226	*RPL7*	ribosomal protein L7	-2.48	0.027036
A_33_P3334443	*FAM69A*	family with sequence similarity 69, member A	-2.47	0.011886
A_23_P380928	*ARPC4-TTLL3*	ARPC4-TTLL3 readthrough	-2.47	0.041709
A_32_P341615	*C9orf84*	chromosome 9 open reading frame 84	-2.47	0.020594
A_33_P3384392	*ADRBK1*	adrenergic, beta, receptor kinase 1	-2.47	0.016060
A_33_P3349414	*ZBTB43*	zinc finger and BTB domain containing 43	-2.47	0.026516
A_23_P130158	*WNT3*	wingless-type MMTV integration site family, member 3	-2.47	0.008741
A_24_P322847	*POLR3H*	polymerase (RNA) III (DNA directed) polypeptide H (22.9kD)	-2.46	0.002305
A_24_P178093	*TOMM40*	translocase of outer mitochondrial membrane 40 homolog (yeast)	-2.46	0.041650
A_23_P10194	*SEZ6L2*	seizure related 6 homolog (mouse)-like 2	-2.45	0.038169

A_23_P129786	*SREBF1*	sterol regulatory element binding transcription factor 1	-2.44	0.035213
A_23_P66599	*VPS25*	vacuolar protein sorting 25 homolog (S. cerevisiae)	-2.43	0.001661
A_33_P3371663	*LTK*	leukocyte receptor tyrosine kinase	-2.43	0.023000
A_23_P106127	*KIAA0586*	KIAA0586	-2.43	0.003216
A_23_P24751	*TTC9C*	tetratricopeptide repeat domain 9C	-2.43	0.033178
A_32_P211045	*DHFR*	dihydrofolate reductase	-2.43	0.023413
A_33_P3219090	*INSIG1*	insulin induced gene 1	-2.43	0.037288
A_23_P365817	*PPP1R14B*	protein phosphatase 1, regulatory (inhibitor) subunit 14B	-2.43	0.013666
A_33_P3233981	*NASP*	nuclear autoantigenic sperm protein (histone-binding)	-2.43	0.020838
A_33_P3663705	*CROCC*	ciliary rootlet coiled-coil, rootletin	-2.43	0.018211
A_23_P24444	*DHCR7*	7-dehydrocholesterol reductase	-2.42	0.021550
A_33_P3289422	*ZNF765*	zinc finger protein 765	-2.42	0.002288
A_23_P19369	*LRRC16A*	leucine rich repeat containing 16A	-2.42	0.046950
A_23_P331700	*SRRM3*	serine/arginine repetitive matrix 3	-2.41	0.001464
A_23_P25194	*HRK*	harakiri, BCL2 interacting protein	-2.41	0.026153
A_19_P00316235	*C5orf56*	chromosome 5 open reading frame 56	-2.41	0.017114
A_33_P3272698	*DUSP23*	dual specificity phosphatase 23	-2.40	0.042394
A_23_P208961	*MUM1*	melanoma associated antigen (mutated) 1	-2.39	0.003271
A_33_P3239287	*CHD3*	chromodomain helicase DNA binding protein 3	-2.39	0.004957
A_33_P3764802	*SIRT5*	sirtuin 5	-2.39	0.045625
A_33_P3388691	*RGS21*	regulator of G-protein signaling 21	-2.39	0.009052
A_24_P6083	*CHCHD10*	coiled-coil-helix-coiled-coil-helix domain containing 10	-2.39	0.003264
A_33_P3410459	*SCARB2*	scavenger receptor class B, member 2	-2.39	0.026275
A_33_P3275835	*TOR2A*	torsin family 2, member A	-2.38	0.022999
A_33_P3410836	*HIST1H4D*	histone cluster 1, H4d	-2.37	0.048236
A_23_P411157	*WNT1*	wingless-type MMTV integration site family, member 1	-2.37	0.032777
A_23_P200493	*LBR*	lamin B receptor	-2.36	0.044373
A_23_P97990	*HTRA1*	HtrA serine peptidase 1	-2.36	0.018507
A_23_P208674	**EMC10*	ER membrane protein complex subunit 10	-2.34	0.036201
A_24_P184555	*PXN*	paxillin	-2.34	0.026317
A_33_P3349334	*SOCS4*	suppressor of cytokine signaling 4	-2.34	0.012431
A_23_P137948	*NENF*	neudesin neurotrophic factor	-2.34	0.001102
A_23_P50535	*DMPK*	dystrophia myotonica-protein kinase	-2.34	0.007432
A_23_P40174	*MMP9*	matrix metallopeptidase 9 (gelatinase B, 92kDa gelatinase, 92kDa type IV collagenase)	-2.34	0.024955
A_33_P3329187	*DNMT1*	DNA (cytosine-5-)-methyltransferase 1	-2.33	0.021722
A_23_P331049	*DPYSL4*	dihydropyrimidinase-like 4	-2.32	0.046450
A_23_P209740	*PSMD1*	proteasome (prosome, macropain) 26S subunit, non-ATPase, 1	-2.32	0.035327
A_23_P361049	*MYO1B*	myosin IB	-2.32	0.038762
A_33_P3418731	*ZNF542P*	zinc finger protein 542, pseudogene	-2.31	0.037300
A_33_P3245011	*DAK*	dihydroxyacetone kinase 2 homolog (S. cerevisiae)	-2.31	0.042831
A_23_P420361	*BRK1*	BRICK1, SCAR/WAVE actin-nucleating complex subunit	-2.30	0.045105
A_33_P3394243	*C1orf101*	chromosome 1 open reading frame 101	-2.29	0.040494
A_23_P210920	*GSS*	glutathione synthetase	-2.29	0.019511
A_24_P37939	**RPS14*	ribosomal protein S14	-2.29	0.048429

A_23_P50942	*RAB3GAP1*	RAB3 GTPase activating protein subunit 1 (catalytic)	-2.29	0.004042
A_23_P207125	*NLGN2*	neuroligin 2	-2.29	0.008303
A_33_P3224380	*DLG1*	discs, large homolog 1 (Drosophila)	-2.29	0.003476
A_24_P68222	*CD99P1*	CD99 molecule pseudogene 1	-2.28	0.031832
A_33_P3319920	*KDM4B*	lysine (K)-specific demethylase 4B	-2.28	0.046387
A_23_P422071	*B3GALT4*	UDP-Gal:betaGlcNAc beta 1,3-galactosyltransferase, polypeptide 4	-2.27	0.010958
A_23_P24345	*SLC39A13*	solute carrier family 39 (zinc transporter), member 13	-2.27	0.032478
A_23_P52336	*UNC5B*	unc-5 homolog B (C. elegans)	-2.27	0.018726
A_33_P3396008	*AGER*	advanced glycosylation end product-specific receptor	-2.25	0.049124
A_24_P242132	*NRBP2*	nuclear receptor binding protein 2	-2.25	0.032079
A_23_P88099	*MCF2L*	MCF.2 cell line derived transforming sequence-like	-2.25	0.045209
A_23_P119943	*IGFBP2*	insulin-like growth factor binding protein 2, 36kDa	-2.25	0.045444
A_19_P00812250	*SMAD4*	SMAD family member 4	-2.24	0.022056
A_24_P712562	*C17orf67*	chromosome 17 open reading frame 67	-2.24	0.041120
A_23_P137381	*ID3*	inhibitor of DNA binding 3, dominant negative helix-loop-helix protein	-2.24	0.008081
A_33_P3232011	*RAB17*	RAB17, member RAS oncogene family	-2.24	0.001008
A_32_P142028	*HNRNPC*	heterogeneous nuclear ribonucleoprotein C (C1/C2)	-2.23	0.008158
A_33_P3329477	*RPL13P5*	ribosomal protein L13 pseudogene 5	-2.23	0.020810
A_33_P3211604	*RAP1B*	RAP1B, member of RAS oncogene family	-2.22	0.027856
A_33_P3302115	*CAPRIN1*	cell cycle associated protein 1	-2.22	0.006974
A_23_P8432	*COPS6*	COP9 signalosome subunit 6	-2.22	0.015761
A_24_P74160	*SNRPD2*	small nuclear ribonucleoprotein D2 polypeptide 16.5kDa	-2.21	0.018327
A_23_P61531	*GLB1*	galactosidase, beta 1	-2.21	0.019289
A_23_P252211	*MTRR*	5-methyltetrahydrofolate-homocysteine methyltransferase reductase	-2.21	0.019123
A_33_P3685216	*A1BG*	alpha-1-B glycoprotein	-2.20	0.038942
A_23_P42353	*ETV7*	ets variant 7	-2.20	0.047097
A_33_P3331687	*GPSM1*	G-protein signaling modulator 1	-2.20	0.034314
A_33_P3359071	*ACD*	adrenocortical dysplasia homolog (mouse)	-2.20	0.019589
A_23_P344988	*ICK*	intestinal cell (MAK-like) kinase	-2.20	0.041345
A_33_P3242748	*ARFRP1*	ADP-ribosylation factor related protein 1	-2.17	0.009826
A_33_P3336617	*ALDH3A2*	aldehyde dehydrogenase 3 family, member A2	-2.17	0.029107
A_23_P100660	*SERPINF1*	serpin peptidase inhibitor, clade F (alpha-2 antiplasmin, pigment epithelium derived factor), member 1	-2.17	0.014282
A_23_P146367	*C9orf89*	chromosome 9 open reading frame 89	-2.17	0.020128
A_24_P383019	*KRTAP13-1*	keratin associated protein 13-1	-2.16	0.016924
A_23_P140563	*TTC23*	tetratricopeptide repeat domain 23	-2.16	0.013759
A_23_P341443	*MNT*	MAX network transcriptional repressor	-2.16	0.026226
A_33_P3387931	*CENPP*	centromere protein P	-2.16	0.039094
A_23_P111981	**LYNX1*	Ly6/neurotoxin 1	-2.16	0.001900
A_23_P383435	*ZNHIT3*	zinc finger, HIT-type containing 3	-2.16	0.044268
A_33_P3324086	*MCUR1*	mitochondrial calcium uniporter regulator 1	-2.16	0.039190
A_23_P15123	*UBFD1*	ubiquitin family domain containing 1	-2.16	0.009342
A_23_P170626	*CHCHD7*	coiled-coil-helix-coiled-coil-helix domain containing 7	-2.15	0.046830
A_23_P64630	*RNF26*	ring finger protein 26	-2.15	0.029980

A_33_P3399268	*IL15RA*	interleukin 15 receptor, alpha	-2.15	0.032871
A_24_P162373	*ZNRF3*	zinc and ring finger 3	-2.15	0.012122
A_23_P210482	*ADA*	adenosine deaminase	-2.15	0.021467
A_33_P3419190	*AREG*	amphiregulin	-2.14	0.039897
A_32_P226149	*YWHAZ*	tyrosine 3-monooxygenase/tryptophan 5-monooxygenase activation protein, zeta	-2.14	0.043070
A_23_P114952	*TMEM9*	transmembrane protein 9	-2.13	0.008691
A_23_P213431	*GFM2*	G elongation factor, mitochondrial 2	-2.12	0.033357
A_23_P377819	*SRSF5*	serine/arginine-rich splicing factor 5	-2.12	0.017090
A_23_P23006	*NRD1*	nardilysin (N-arginine dibasic convertase)	-2.11	0.009805
A_33_P3238976	*TRMT5*	tRNA methyltransferase 5	-2.11	0.011661
A_33_P3332671	*PKD1L3*	polycystic kidney disease 1-like 3	-2.11	0.040747
A_33_P3211473	*XPO7*	exportin 7	-2.11	0.041681
A_24_P382287	*CDH7*	cadherin 7, type 2	-2.11	0.021313
A_33_P3273457	*SPATA21*	spermatogenesis associated 21	-2.11	0.029713
A_33_P3252286	*CRLF1*	cytokine receptor-like factor 1	-2.10	0.048045
A_33_P3415625	*LRRIQ1*	leucine-rich repeats and IQ motif containing 1	-2.10	0.023575
A_23_P92120	*SNTN*	sentan, cilia apical structure protein	-2.10	0.030988
A_23_P2083	*ASCL3*	achaete-scute family bHLH transcription factor 3	-2.10	0.027892
A_23_P356163	*WDR49*	WD repeat domain 49	-2.10	0.042202
A_23_P112341	*RMI1*	RecQ mediated genome instability 1	-2.10	0.046430
A_33_P3260062	*PIAS4*	protein inhibitor of activated STAT, 4	-2.10	0.014970
A_23_P145777	*NDUFA4*	NADH dehydrogenase (ubiquinone) 1 alpha subcomplex, 4, 9kDa	-2.09	0.037584
A_24_P576174	*DCP1A*	decapping mRNA 1A	-2.09	0.044855
A_33_P3268464	*MPDU1*	mannose-P-dolichol utilization defect 1	-2.09	0.031849
A_33_P3309929	*HDAC3*	histone deacetylase 3	-2.09	0.033813
A_23_P63219	*POGZ*	pogo transposable element with ZNF domain	-2.08	0.007804
A_33_P3370364	*PRLHR*	prolactin releasing hormone receptor	-2.08	0.026747
A_23_P33216	*ATP5B*	ATP synthase, H+ transporting, mitochondrial F1 complex, beta polypeptide	-2.08	0.004528
A_23_P28084	*STX10*	syntaxin 10	-2.07	0.019060
A_23_P301340	*ENTHD2*	ENTH domain containing 2	-2.07	0.010608
A_23_P365412	*MFSD4*	major facilitator superfamily domain containing 4	-2.07	0.044712
A_33_P3239152	*RABEP2*	rabaptin, RAB GTPase binding effector protein 2	-2.07	0.024570
A_33_P3356877	*OR13C3*	olfactory receptor, family 13, subfamily C, member 3	-2.07	0.001264
A_23_P42829	*SND1*	staphylococcal nuclease and tudor domain containing 1	-2.07	0.043302
A_24_P354496	*WWC2-AS2*	WWC2 antisense RNA 2	-2.06	0.047024
A_23_P14876	*SRP14*	signal recognition particle 14kDa (homologous Alu RNA binding protein)	-2.06	0.033846
A_33_P3271657	**HHIPL1*	HHIP-like 1	-2.06	0.029947
A_23_P405885	*DPPA2*	developmental pluripotency associated 2	-2.06	0.001340
A_33_P3313411	*ARHGAP33*	Rho GTPase activating protein 33	-2.06	0.006014
A_33_P3214466	*MESP1*	mesoderm posterior 1 homolog (mouse)	-2.06	0.025231
A_33_P3406171	*PABPC5*	poly(A) binding protein, cytoplasmic 5	-2.06	0.010306
A_33_P3424467	*CNST*	consortin, connexin sorting protein	-2.06	0.007615
A_23_P256375	*STX4*	syntaxin 4	-2.05	0.035196

A_23_P5601	*DOK1*	docking protein 1, 62kDa (downstream of tyrosine kinase 1)	-2.05	0.039488
A_19_P00316448	*LINGO1*	leucine rich repeat and Ig domain containing 1	-2.05	0.036781
A_24_P11061	*CSAG1*	chondrosarcoma associated gene 1	-2.05	0.007586
A_23_P371861	*CNBD1*	cyclic nucleotide binding domain containing 1	-2.05	0.006316
A_33_P3417810	*NOL10*	nucleolar protein 10	-2.04	0.006697
A_23_P306346	*C9orf41*	chromosome 9 open reading frame 41	-2.04	0.030370
A_23_P29836	*TMEM42*	transmembrane protein 42	-2.04	0.034882
A_23_P205789	*GABPB1*	GA binding protein transcription factor, beta subunit 1	-2.03	0.014814
A_23_P428184	*HIST1H2AD*	histone cluster 1, H2ad	-2.02	0.007767
A_23_P107963	*FUT1*	fucosyltransferase 1 (galactoside 2-alpha-L-fucosyltransferase, H blood group)	-2.02	0.022400
A_23_P41025	*GNL3*	guanine nucleotide binding protein-like 3 (nucleolar)	-2.02	0.005163
A_33_P3236035	*KDM4C*	lysine (K)-specific demethylase 4C	-2.02	0.046255
A_23_P55518	*SMAD7*	SMAD family member 7	-2.02	0.020661
A_19_P00319862	*MEG3*	maternally expressed 3 (non-protein coding)	-2.00	0.039293
A_23_P122915	*BRI3*	brain protein I3	-2.00	0.022074
A_33_P3411025	*ARHGAP19*	Rho GTPase activating protein 19	-2.00	0.044429
A_23_P102890	*MRPS6*	mitochondrial ribosomal protein S6	-2.00	0.017230
A_33_P3389638	*NOP14*	NOP14 nucleolar protein	-1.99	0.026140
A_24_P348989	*LILRA1*	leukocyte immunoglobulin-like receptor, subfamily A (with TM domain), member 1	-1.98	0.042127
A_23_P159937	*SLC6A8*	solute carrier family 6 (neurotransmitter transporter), member 8	-1.98	0.017493
A_32_P198523	*FSIP2*	fibrous sheath interacting protein 2	-1.98	0.038975
A_23_P109881	*ITIH4*	inter-alpha-trypsin inhibitor heavy chain family, member 4	-1.98	0.043698
A_24_P135276	*USP42*	ubiquitin specific peptidase 42	-1.98	0.028404
A_24_P923757	*ATF7IP*	activating transcription factor 7 interacting protein	-1.97	0.019752
A_23_P95302	*RFC5*	replication factor C (activator 1) 5, 36.5kDa	-1.97	0.021868
A_33_P3280531	*CRAT*	carnitine O-acetyltransferase	-1.97	0.018234
A_33_P3239228	*MUC3A*	mucin 3A, cell surface associated	-1.97	0.048221
A_23_P135474	*MRPL37*	mitochondrial ribosomal protein L37	-1.97	0.002515
A_33_P3394404	*TSPAN32*	tetraspanin 32	-1.97	0.033998
A_23_P324754	*CEMIP*	cell migration inducing protein, hyaluronan binding	-1.97	0.039753
A_23_P344578	*FAM154A*	family with sequence similarity 154, member A	-1.96	0.021723
A_23_P19938	*KDELR2*	KDEL (Lys-Asp-Glu-Leu) endoplasmic reticulum protein retention receptor 2	-1.96	0.016239
A_33_P3352103	*LYPLAL1*	lysophospholipase-like 1	-1.96	0.015318
A_33_P3267380	*SGCD*	sarcoglycan, delta (35kDa dystrophin-associated glycoprotein)	-1.95	0.018517
A_33_P3251727	*RYR2*	ryanodine receptor 2 (cardiac)	-1.95	0.020818
A_23_P96990	*NVL*	nuclear VCP-like	-1.95	0.010439
A_33_P3416882	*ARL9*	ADP-ribosylation factor-like 9	-1.95	0.044525
A_23_P350045	*REEP5*	receptor accessory protein 5	-1.94	0.026235
A_23_P414899	*TTC17*	tetratricopeptide repeat domain 17	-1.94	0.005421
A_23_P398449	*VNN3*	vanin 3	-1.93	0.007946
A_33_P3373298	*STAG3L4*	stromal antigen 3-like 4 (pseudogene)	-1.93	0.030499
A_33_P3309734	*CCDC108*	coiled-coil domain containing 108	-1.93	0.041365
A_24_P263310	*FAM169B*	family with sequence similarity 169, member B	-1.93	0.015127

A_33_P3424302	*SPINT4*	serine peptidase inhibitor, Kunitz type 4	-1.92	0.004192
A_23_P15357	*LGALS3BP*	lectin, galactoside-binding, soluble, 3 binding protein	-1.92	0.012000
A_33_P3570208	*LPP-AS2*	LPP antisense RNA 2	-1.92	0.011692
A_23_P389465	*KSR2*	kinase suppressor of ras 2	-1.92	0.045595
A_33_P3326713	*FAM188B*	family with sequence similarity 188, member B	-1.91	0.003848
A_23_P106973	*41891*	septin 9	-1.90	0.032034
A_33_P3390057	*TM4SF1*	transmembrane 4 L six family member 1	-1.90	0.030685
A_23_P161171	*ASAH2*	N-acylsphingosine amidohydrolase (non-lysosomal ceramidase) 2	-1.90	0.009081
A_23_P138271	*ARL8A*	ADP-ribosylation factor-like 8A	-1.90	0.049433
A_32_P6015	*MNX1*	motor neuron and pancreas homeobox 1	-1.89	0.025869
A_33_P3251289	*VIMP*	VCP-interacting membrane protein	-1.89	0.022236
A_33_P3409090	*CNTN1*	contactin 1	-1.88	0.022485
A_33_P3335511	*FCRL5*	Fc receptor-like 5	-1.88	0.009098
A_23_P10685	*HSPBP1*	HSPA (heat shock 70kDa) binding protein, cytoplasmic cochaperone 1	-1.88	0.009541
A_24_P7121	*NSUN7*	NOP2/Sun domain family, member 7	-1.87	0.012452
A_33_P3244424	*SNRPE*	small nuclear ribonucleoprotein polypeptide E	-1.87	0.049127
A_23_P106859	*EMC8*	ER membrane protein complex subunit 8	-1.87	0.025744
A_23_P65157	*COX17*	COX17 cytochrome c oxidase copper chaperone	-1.86	0.016171
A_32_P129288	*RAB1B*	RAB1B, member RAS oncogene family	-1.86	0.035881
A_33_P3229017	*FAM53C*	family with sequence similarity 53, member C	-1.86	0.002845
A_33_P3384133	*C17orf99*	chromosome 17 open reading frame 99	-1.85	0.024285
A_23_P120883	*HMOX1*	heme oxygenase (decycling) 1	-1.85	0.002414
A_23_P17204	*ANAPC1*	anaphase promoting complex subunit 1	-1.84	0.038014
A_23_P1352	*SFRP5*	secreted frizzled-related protein 5	-1.84	0.026051
A_33_P3766913	*DEFA10P*	defensin, alpha 10 pseudogene	-1.83	0.013838
A_23_P155539	*POU1F1*	POU class 1 homeobox 1	-1.83	0.043150
A_23_P209619	*ATL2*	atlastin GTPase 2	-1.83	0.003900
A_23_P201459	*IFI6*	interferon, alpha-inducible protein 6	-1.83	0.021729
A_33_P3311041	*SHB*	Src homology 2 domain containing adaptor protein B	-1.82	0.017216
A_23_P89799	*ACAA2*	acetyl-CoA acyltransferase 2	-1.82	0.046006
A_23_P65031	*DYNLL1*	dynein, light chain, LC8-type 1	-1.82	0.043230
A_23_P425750	*ARMC6*	armadillo repeat containing 6	-1.82	0.004881
A_24_P244442	*BSCL2*	Berardinelli-Seip congenital lipodystrophy 2 (seipin)	-1.82	0.013402
A_23_P318890	*OR1F1*	olfactory receptor, family 1, subfamily F, member 1	-1.81	0.036450
A_33_P3384543	*POMGNT1*	protein O-linked mannose N-acetylglucosaminyltransferase 1 (beta 1,2-)	-1.81	0.019037
A_23_P83351	*DFNB31*	deafness, autosomal recessive 31	-1.81	0.028258
A_33_P3307267	*VWF*	von Willebrand factor	-1.80	0.015608
A_23_P156284	*DBN1*	drebrin 1	-1.80	0.023811
A_33_P3249135	*NBEAL1*	neurobeachin-like 1	-1.80	0.012790
A_33_P3408949	*LOXHD1*	lipoxygenase homology domains 1	-1.79	0.002895
A_19_P00801627	*ANP32AP1*	acidic (leucine-rich) nuclear phosphoprotein 32 family, member A pseudogene 1	-1.79	0.036174
A_33_P3292560	*SURF1*	surfeit 1	-1.79	0.043535
A_32_P107029	*NAPSA*	napsin A aspartic peptidase	-1.79	0.002991

A_23_P130027	*EPN3*	epsin 3	-1.79	0.044127
A_33_P3359268	*HMG20B*	high mobility group 20B	-1.78	0.048055
A_32_P216734	*SPDYE3*	speedy/RINGO cell cycle regulator family member E3	-1.78	0.015840
A_23_P60962	*MAPK15*	mitogen-activated protein kinase 15	-1.78	0.007831
A_24_P696761	*LEMD1*	LEM domain containing 1	-1.78	0.009747
A_23_P49041	*TMEM62*	transmembrane protein 62	-1.77	0.029949
A_23_P256561	*TLR6*	toll-like receptor 6	-1.76	0.006419
A_19_P00317984	**SNHG5*	small nucleolar RNA host gene 5 (non-protein coding)	-1.76	0.004601
A_23_P256455	*RPA3*	replication protein A3, 14kDa	-1.75	0.045202
A_33_P3351693	*LOC643733*	caspase 4, apoptosis-related cysteine peptidase pseudogene	-1.75	0.044058
A_33_P3289005	*AP1S3*	adaptor-related protein complex 1, sigma 3 subunit	-1.74	0.018891
A_33_P3365760	*STAP1*	signal transducing adaptor family member 1	-1.74	0.018760
A_33_P3402725	*ASH1L*	ash1 (absent, small, or homeotic)-like (Drosophila)	-1.74	0.036570
A_23_P427148	*PROCA1*	protein interacting with cyclin A1	-1.74	0.025458
A_23_P130435	*LIM2*	lens intrinsic membrane protein 2, 19kDa	-1.74	0.016046
A_33_P3368313	*MT1H*	metallothionein 1H	-1.73	0.008279
A_23_P166	*MOB3C*	MOB kinase activator 3C	-1.73	0.014473
A_33_P3295029	*KBTBD12*	kelch repeat and BTB (POZ) domain containing 12	-1.73	0.024978
A_33_P3380472	*HCAR1*	hydroxycarboxylic acid receptor 1	-1.73	0.015220
A_23_P30464	*PRR7*	proline rich 7 (synaptic)	-1.73	0.037967
A_23_P352389	*SPATA32*	spermatogenesis associated 32	-1.72	0.035816
A_33_P3269924	*HIP1R*	huntingtin interacting protein 1 related	-1.72	0.015900
A_24_P156635	*TEX35*	testis expressed 35	-1.72	0.023247
A_33_P3338186	*HEXDC*	hexosaminidase (glycosyl hydrolase family 20, catalytic domain) containing	-1.72	0.016309
A_33_P3308949	*DBT*	dihydrolipoamide branched chain transacylase E2	-1.72	0.036341
A_33_P3381147	*KRTAP5-3*	keratin associated protein 5-3	-1.72	0.041450
A_23_P428729	*ZMYM6*	zinc finger, MYM-type 6	-1.72	0.034432
A_33_P3237201	*SYT1*	synaptotagmin I	-1.71	0.019137
A_23_P320261	*DMKN*	dermokine	-1.71	0.023174
A_32_P182662	*AIDA*	axin interactor, dorsalization associated	-1.71	0.019158
A_23_P356667	*CCDC83*	coiled-coil domain containing 83	-1.71	0.030935
A_33_P3344334	*KCNH5*	potassium voltage-gated channel, subfamily H (eag-related), member 5	-1.71	0.020969
A_33_P3285299	*GPRIN2*	G protein regulated inducer of neurite outgrowth 2	-1.70	0.031897
A_33_P3417150	*P2RY1*	purinergic receptor P2Y, G-protein coupled, 1	-1.69	0.019728
A_33_P3840512	*SLC25A15*	solute carrier family 25 (mitochondrial carrier; ornithine transporter) member 15	-1.68	0.040378
A_32_P231179	*TEKT4*	tektin 4	-1.68	0.011742
A_23_P17456	*SIRPB1*	signal-regulatory protein beta 1	-1.68	0.034287
A_23_P29922	*TLR3*	toll-like receptor 3	-1.68	0.027064
A_23_P76914	*SIX1*	SIX homeobox 1	-1.68	0.011295
A_24_P377124	*THPO*	thrombopoietin	-1.67	0.026537
A_23_P146644	*ANXA2*	annexin A2	-1.67	0.032765
A_23_P40240	*CTSZ*	cathepsin Z	-1.67	0.002361
A_23_P16242	*ZNF20*	zinc finger protein 20	-1.67	0.002458

A_23_P82859	*OSGIN2*	oxidative stress induced growth inhibitor family member 2	-1.66	0.044457
A_33_P3246026	*DEFB131*	defensin, beta 131	-1.66	0.015463
A_23_P3038	*GPX2*	glutathione peroxidase 2 (gastrointestinal)	-1.66	0.038254
A_23_P90855	*FARP2*	FERM, RhoGEF and pleckstrin domain protein 2	-1.66	0.033034
A_23_P204246	*PHC1*	polyhomeotic homolog 1 (Drosophila)	-1.66	0.018242
A_33_P3367731	*SLC24A2*	solute carrier family 24 (sodium/potassium/calcium exchanger), member 2	-1.66	0.018760
A_33_P3211138	*CADM1*	cell adhesion molecule 1	-1.65	0.014502
A_23_P58466	*SMN1*	survival of motor neuron 1, telomeric	-1.65	0.034263
A_33_P3386262	*CDT1*	chromatin licensing and DNA replication factor 1	-1.65	0.037382
A_33_P3234197	*TRIM45*	tripartite motif containing 45	-1.65	0.032736
A_33_P3235716	*SPSB3*	splA/ryanodine receptor domain and SOCS box containing 3	-1.65	0.012005
A_33_P3267420	*APOB*	apolipoprotein B	-1.64	0.038862
A_33_P3393200	*SRRM4*	serine/arginine repetitive matrix 4	-1.64	0.028822
A_33_P3318027	*CSNK2B*	casein kinase 2, beta polypeptide	-1.64	0.034991
A_23_P204252	*M6PR*	mannose-6-phosphate receptor (cation dependent)	-1.64	0.044623
A_23_P52227	*GDF10*	growth differentiation factor 10	-1.63	0.029955
A_23_P49181	*LRRC29*	leucine rich repeat containing 29	-1.63	0.035701
A_23_P86855	*MACROD1*	MACRO domain containing 1	-1.63	0.025507
A_23_P407142	*LUZP1*	leucine zipper protein 1	-1.63	0.038735
A_33_P3241316	*PLA2G4F*	phospholipase A2, group IVF	-1.63	0.044023
A_23_P131263	*MPP4*	membrane protein, palmitoylated 4 (MAGUK p55 subfamily member 4)	-1.63	0.028623
A_33_P3214785	*LEPROT*	leptin receptor overlapping transcript	-1.62	0.015126
A_33_P3292417	*WDR5*	WD repeat domain 5	-1.61	0.038369
A_23_P334218	*TBC1D31*	TBC1 domain family, member 31	-1.60	0.039313
A_23_P615	*INSRR*	insulin receptor-related receptor	-1.60	0.031273
A_33_P3267799	*LILRB4*	leukocyte immunoglobulin-like receptor, subfamily B (with TM and ITIM domains), member 4	-1.60	0.025887
A_23_P139418	*GALNT18*	polypeptide N-acetylgalactosaminyltransferase 18	-1.60	0.026972
A_32_P195719	*CENPVP2*	centromere protein V pseudogene 2	-1.60	0.015376
A_19_P00317490	*CCDC141*	coiled-coil domain containing 141	-1.60	0.036587
A_23_P500400	*ABCA6*	ATP-binding cassette, sub-family A (ABC1), member 6	-1.60	0.049875
A_33_P3269779	*ZNF3*	zinc finger protein 3	-1.60	0.025823
A_23_P48109	*NINJ2*	ninjurin 2	-1.60	0.015963
A_33_P3309491	*PTPRU*	protein tyrosine phosphatase, receptor type, U	-1.60	0.030181
A_23_P24244	*FAM208B*	family with sequence similarity 208, member B	-1.60	0.006781
A_33_P3244593	*FHAD1*	forkhead-associated (FHA) phosphopeptide binding domain 1	-1.59	0.027088
A_23_P62764	*CCDC28B*	coiled-coil domain containing 28B	-1.59	0.009474
A_33_P3389678	*SPPL2B*	signal peptide peptidase like 2B	-1.59	0.043557
A_23_P346327	*FLJ30679*	uncharacterized protein FLJ30679	-1.58	0.009702
A_32_P19000	*TNRC6C-AS1*	TNRC6C antisense RNA 1	-1.58	0.018962
A_24_P250815	*POF1B*	premature ovarian failure, 1B	-1.57	0.038527
A_24_P333019	*RNF24*	ring finger protein 24	-1.57	0.047548
A_23_P363826	*FBXO3*	F-box protein 3	-1.57	0.043619
A_33_P3270435	*HIGD2B*	HIG1 hypoxia inducible domain family, member 2B	-1.57	0.034067

A_32_P141682	*EVPLL*	envoplakin-like	-1.57	0.038024
A_24_P76854	*KRTAP2-1*	keratin associated protein 2-1	-1.56	0.043735
A_33_P3217322	*TTC40*	tetratricopeptide repeat domain 40	-1.56	0.038660
A_33_P3388822	*ZNF233*	zinc finger protein 233	-1.56	0.024278
A_33_P3329128	*LOC729930*	chromosome 11 open reading frame 74 pseudogene	-1.56	0.042352
A_23_P412515	*CLDN12*	claudin 12	-1.56	0.039867
A_24_P355626	*ABCG4*	ATP-binding cassette, sub-family G (WHITE), member 4	-1.56	0.044841
A_33_P3816042	*ACTR3BP2*	ACTR3B pseudogene 2	-1.56	0.008585
A_24_P645765	*KLHL42*	kelch-like family member 42	-1.56	0.032372
A_24_P337746	*RABGEF1*	RAB guanine nucleotide exchange factor (GEF) 1	-1.56	0.033656
A_33_P3292724	*SZT2*	seizure threshold 2 homolog (mouse)	-1.55	0.032957
A_32_P452655	*LGALS9C*	lectin, galactoside-binding, soluble, 9C	-1.55	0.031651
A_23_P120103	*KCNS3*	potassium voltage-gated channel, delayed-rectifier, subfamily S, member 3	-1.55	0.047070
A_23_P27075	*GABARAP*	GABA(A) receptor-associated protein	-1.55	0.038903
A_23_P13486	*C11orf16*	chromosome 11 open reading frame 16	-1.55	0.036039
A_23_P107661	*PPP2R1A*	protein phosphatase 2, regulatory subunit A, alpha	-1.55	0.024960
A_24_P914495	*MARK2*	MAP/microtubule affinity-regulating kinase 2	-1.55	0.020762
A_33_P3215929	*PRR5*	proline rich 5 (renal)	-1.55	0.025322
A_33_P3398513	*LOC728819*	hCG1645220	-1.55	0.040032
A_19_P00321817	*KC6*	keratoconus gene 6	-1.55	0.019243
A_23_P134347	*CPVL*	carboxypeptidase, vitellogenic-like	-1.55	0.033802
A_33_P3372859	*DDX54*	DEAD (Asp-Glu-Ala-Asp) box polypeptide 54	-1.55	0.012200
A_33_P3414362	*USP32*	ubiquitin specific peptidase 32	-1.54	0.049732
A_33_P3222183	*DPYSL5*	dihydropyrimidinase-like 5	-1.54	0.040532
A_33_P3315149	*HEXA-AS1*	HEXA antisense RNA 1	-1.54	0.040024
A_33_P3254751	*ST6GALNAC6*	ST6 (alpha-N-acetyl-neuraminyl-2,3-beta-galactosyl-1,3)-N-acetylgalactosaminide alpha-2,6-sialyltransferase 6	-1.54	0.038971
A_23_P133536	*CAPSL*	calcyphosine-like	-1.53	0.044959
A_23_P13493	*OR51E1*	olfactory receptor, family 51, subfamily E, member 1	-1.53	0.019942
A_33_P3308142	*OR4F13P*	olfactory receptor, family 4, subfamily F, member 13 pseudogene	-1.53	0.031581
A_23_P137157	*RENBP*	renin binding protein	-1.53	0.016145
A_33_P3307536	*FAM230B*	family with sequence similarity 230, member B (non-protein coding)	-1.52	0.037886
A_23_P119222	*RETN*	resistin	-1.52	0.025329
A_33_P3236109	*OCA2*	oculocutaneous albinism II	-1.52	0.042312
A_23_P328740	*NEURL3*	neuralized E3 ubiquitin protein ligase 3	-1.52	0.047415
A_33_P3358213	*PADI6*	peptidyl arginine deiminase, type VI	-1.52	0.038076
A_23_P57910	*RTP3*	receptor (chemosensory) transporter protein 3	-1.52	0.031746
A_23_P66328	*ACSM2B*	acyl-CoA synthetase medium-chain family member 2B	-1.52	0.028943
A_24_P22746	*ZADH2*	zinc binding alcohol dehydrogenase domain containing 2	-1.51	0.035329
A_23_P391857	*ESRRB*	estrogen-related receptor beta	-1.51	0.041667
A_33_P3249888	*BEST3*	bestrophin 3	-1.51	0.048518
A_33_P3221683	*SPATA45*	spermatogenesis associated 45	-1.51	0.038603
A_33_P3264657	*CYP27C1*	cytochrome P450, family 27, subfamily C, polypeptide 1	-1.51	0.032111
A_33_P3311717	*TGIF1*	TGFB-induced factor homeobox 1	-1.51	0.023660

A_32_P203029	*FAM9B*	family with sequence similarity 9, member B	-1.51	0.031802
A_23_P19291	*TUBB2A*	tubulin, beta 2A class IIa	-1.50	0.027543
A_24_P626932	*MUC3*	intestinal mucin-like	-1.50	0.012663
A_33_P3319856	*SH2D4B*	SH2 domain containing 4B	-1.50	0.033334
A_24_P90900	*CTRL*	chymotrypsin-like	-1.50	0.047058
A_19_P00808367	*PPP4R1L*	protein phosphatase 4, regulatory subunit 1-like	-1.50	0.008110
A_33_P3391895	*HRNR*	hornerin	-1.50	0.027853
A_33_P3264672	*CYP26C1*	cytochrome P450, family 26, subfamily C, polypeptide 1	-1.50	0.049009
A_23_P93602	*C6orf58*	chromosome 6 open reading frame 58	-1.50	0.009728
A_23_P339117	*NKAIN3*	Na+/K+ transporting ATPase interacting 3	-1.50	0.033244
A_23_P257815	*CD180*	CD180 molecule	-1.50	0.047105
A_33_P3371360	*METTL24*	methyltransferase like 24	-1.50	0.046818
A_23_P139912	*IGFBP6*	insulin-like growth factor binding protein 6	-1.50	0.049429
A_33_P3252661	*POTEA*	POTE ankyrin domain family, member A	-1.50	0.020921
A_23_P64661	*ARHGAP9*	Rho GTPase activating protein 9	-1.50	0.043487
A_23_P45234	*ZNF41*	zinc finger protein 41	-1.50	0.032319
A_33_P3345796	*SNX3*	sorting nexin 3	-1.50	0.024191
A_33_P3319276	*FAM178B*	family with sequence similarity 178, member B	-1.49	0.026245
A_23_P85963	*OR6Y1*	olfactory receptor, family 6, subfamily Y, member 1	-1.48	0.040489
A_33_P3420382	*LOC100131492*	PP12901	-1.48	0.046749
A_33_P3238548	*C3orf36*	chromosome 3 open reading frame 36	-1.48	0.020253
A_24_P919899	*ATPAF1*	ATP synthase mitochondrial F1 complex assembly factor 1	-1.48	0.035421
A_33_P3253628	*PHYKPL*	5-phosphohydroxy-L-lysine phospho-lyase	-1.48	0.038623
A_32_P139738	*HERC2P4*	hect domain and RLD 2 pseudogene 4	-1.48	0.048673
A_33_P3298460	*GCG*	glucagon	-1.48	0.023870
A_19_P00325300	*RPSA*	ribosomal protein SA	-1.48	0.048308
A_23_P115161	*ACKR1*	atypical chemokine receptor 1 (Duffy blood group)	-1.47	0.032114
A_23_P25503	*FNDC3A*	fibronectin type III domain containing 3A	-1.47	0.049328
A_23_P41314	*F11*	coagulation factor XI	-1.47	0.027454
A_23_P416191	*TAS2R31*	taste receptor, type 2, member 31	-1.47	0.041976
A_23_P12746	*MRC1*	mannose receptor, C type 1	-1.47	0.011525
A_32_P167904	*ZNF681*	zinc finger protein 681	-1.47	0.010613
A_33_P3268555	*SP140*	SP140 nuclear body protein	-1.47	0.042710
A_33_P3256303	*SELPLG*	selectin P ligand	-1.47	0.019943
A_23_P126349	*BARHL2*	BarH-like homeobox 2	-1.47	0.010557
A_33_P3393504	*S100A7A*	S100 calcium binding protein A7A	-1.46	0.046289
A_33_P3364904	*ZNF662*	zinc finger protein 662	-1.46	0.025068
A_33_P3312212	*PRKAA1*	protein kinase, AMP-activated, alpha 1 catalytic subunit	-1.46	0.027171
A_33_P3285271	*FMO6P*	flavin containing monooxygenase 6 pseudogene	-1.46	0.045053
A_23_P39755	*B3GNT7*	UDP-GlcNAc:betaGal beta-1,3-N-acetylglucosaminyltransferase 7	-1.46	0.023572
A_33_P3244574	*HPN-AS1*	HPN antisense RNA 1	-1.46	0.025290
A_23_P303833	*SCN4B*	sodium channel, voltage-gated, type IV, beta subunit	-1.46	0.049810
A_23_P360804	*CPNE5*	copine V	-1.46	0.029950
A_24_P713267	*SLCO1B7*	solute carrier organic anion transporter family, member 1B7 (non-functional)	-1.46	0.044624

A_33_P3303319	*LPPR5*	lipid phosphate phosphatase-related protein type 5	-1.46	0.019278
A_33_P3417081	*ABCA17P*	ATP-binding cassette, sub-family A (ABC1), member 17, pseudogene	-1.46	0.039649
A_33_P3382403	*SPATA31A3*	SPATA31 subfamily A, member 3	-1.46	0.028170
A_23_P35820	*CFL1*	cofilin 1 (non-muscle)	-1.45	0.025780
A_33_P3246935	*CSMD2*	CUB and Sushi multiple domains 2	-1.45	0.029324
A_23_P79108	*ATP8B3*	ATPase, aminophospholipid transporter, class I, type 8B, member 3	-1.45	0.018732
A_23_P207245	*TEKT3*	tektin 3	-1.45	0.023660
A_23_P379789	*ST8SIA5*	ST8 alpha-N-acetyl-neuraminide alpha-2,8-sialyltransferase 5	-1.45	0.035785
A_24_P379104	*PIM2*	pim-2 oncogene	-1.45	0.017755
A_23_P24457	*LRRC4C*	leucine rich repeat containing 4C	-1.45	0.013871
A_23_P148121	*EHBP1L1*	EH domain binding protein 1-like 1	-1.45	0.045340
A_33_P3319896	*PRAMEF4*	PRAME family member 4	-1.44	0.041816
A_23_P333038	*WDR96*	WD repeat domain 96	-1.44	0.030654
A_24_P113287	*ZNF229*	zinc finger protein 229	-1.44	0.048227
A_23_P377291	*TGFA*	transforming growth factor, alpha	-1.44	0.033658
A_32_P494620	*LHFPL5*	lipoma HMGIC fusion partner-like 5	-1.44	0.043770
A_23_P26154	*PLIN1*	perilipin 1	-1.44	0.016555
A_23_P321892	*HRG*	histidine-rich glycoprotein	-1.44	0.022620
A_23_P364567	*CLRN1*	clarin 1	-1.44	0.029090
A_33_P3366064	*SOGA1*	suppressor of glucose, autophagy associated 1	-1.44	0.048003
A_32_P325823	*LOC100996634*	transmembrane protein FLJ37396	-1.43	0.039885
A_24_P252462	*TTPA*	tocopherol (alpha) transfer protein	-1.43	0.029391
A_24_P187774	*SVEP1*	sushi, von Willebrand factor type A, EGF and pentraxin domain containing 1	-1.43	0.044173
A_33_P3338798	*SDR16C6P*	short chain dehydrogenase/reductase family 16C, member 6, pseudogene	-1.42	0.020544
A_33_P3278877	*ROBO2*	roundabout, axon guidance receptor, homolog 2 (Drosophila)	-1.42	0.032428
A_24_P254084	*ZNF69*	zinc finger protein 69	-1.42	0.020251
A_33_P3405980	*SULT6B1*	sulfotransferase family, cytosolic, 6B, member 1	-1.42	0.045772
A_23_P169097	*WISP1*	WNT1 inducible signaling pathway protein 1	-1.42	0.031509
A_33_P3310154	*OR51B6*	olfactory receptor, family 51, subfamily B, member 6	-1.42	0.039563
A_32_P178758	*LY86-AS1*	LY86 antisense RNA 1	-1.42	0.028614
A_24_P382026	*MIF4GD*	MIF4G domain containing	-1.42	0.016166
A_32_P65533	*ZC3H12D*	zinc finger CCCH-type containing 12D	-1.41	0.031230
A_33_P3243454	*IGFL3*	IGF-like family member 3	-1.41	0.031931
A_33_P3299982	*USF2*	upstream transcription factor 2, c-fos interacting	-1.41	0.041429
A_33_P3306619	*KRTAP25-1*	keratin associated protein 25-1	-1.41	0.032355
A_23_P6201	*LIPI*	lipase, member I	-1.41	0.032187
A_33_P3420020	*PPIP5K1*	diphosphoinositol pentakisphosphate kinase 1	-1.40	0.049406
A_32_P14762	*OOEP*	oocyte expressed protein	-1.40	0.039713
A_24_P472007	*UNC13A*	unc-13 homolog A (C. elegans)	-1.40	0.041232
A_24_P453497	*RBM20*	RNA binding motif protein 20	-1.40	0.038201
A_23_P128375	*FAM222A*	family with sequence similarity 222, member A	-1.40	0.029325
A_33_P3313441	*FAM220BP*	family with sequence similarity 220, member B, pseudogene	-1.39	0.030858
A_23_P323823	*HIST1H2BA*	histone cluster 1, H2ba	-1.39	0.043927

A_23_P414654	*RAB37*	RAB37, member RAS oncogene family	-1.39	0.037171
A_24_P940275	*FRMPD4*	FERM and PDZ domain containing 4	-1.39	0.029469
A_23_P54488	*ACSBG1*	acyl-CoA synthetase bubblegum family member 1	-1.39	0.038552
A_23_P203488	*SMPD1*	sphingomyelin phosphodiesterase 1, acid lysosomal	-1.39	0.049675
A_23_P79360	*NOSTRIN*	nitric oxide synthase trafficking	-1.39	0.026847
A_23_P18123	*NLGN1*	neuroligin 1	-1.39	0.028075
A_23_P78170	*MYBBP1A*	MYB binding protein (P160) 1a	-1.38	0.030828
A_24_P21985	*FOXJ2*	forkhead box J2	-1.38	0.028490
A_33_P3343790	*OR11L1*	olfactory receptor, family 11, subfamily L, member 1	-1.38	0.033920
A_24_P280664	*GBP7*	guanylate binding protein 7	-1.38	0.037320
A_33_P3343727	*CELA3A*	chymotrypsin-like elastase family, member 3A	-1.38	0.040687
A_24_P385732	*SLC51A*	solute carrier family 51, alpha subunit	-1.38	0.035479
A_24_P410408	*KRT83*	keratin 83	-1.38	0.044996
A_33_P3369581	*EIF3G*	eukaryotic translation initiation factor 3, subunit G	-1.38	0.046987
A_23_P145631	*GIMAP6*	GTPase, IMAP family member 6	-1.38	0.048236
A_23_P422724	*PPIC*	peptidylprolyl isomerase C (cyclophilin C)	-1.37	0.049373
A_19_P00327335	*ZBTB20*	zinc finger and BTB domain containing 20	-1.37	0.042491
A_23_P20697	*ADAMTSL2*	ADAMTS-like 2	-1.37	0.024106
A_23_P103349	*OCLM*	oculomedin	-1.37	0.033535
A_33_P3589033	*DIAPH2-AS1*	DIAPH2 antisense RNA 1	-1.37	0.044495
A_33_P3245517	*LOC441666*	zinc finger protein 91 pseudogene	-1.37	0.049049
A_33_P3370454	*DEFB108B*	defensin, beta 108B	-1.37	0.043033
A_23_P44849	*KCTD13*	potassium channel tetramerization domain containing 13	-1.37	0.036916
A_33_P3288491	*KLHL38*	kelch-like family member 38	-1.37	0.036318
A_23_P43369	*SIT1*	signaling threshold regulating transmembrane adaptor 1	-1.37	0.038575
A_33_P3369944	*ADAM21*	ADAM metallopeptidase domain 21	-1.36	0.043336
A_24_P174793	*PCSK1*	proprotein convertase subtilisin/kexin type 1	-1.36	0.025632
A_32_P213881	*C20orf202*	chromosome 20 open reading frame 202	-1.36	0.042378
A_23_P129629	*MT3*	metallothionein 3	-1.36	0.047827
A_23_P25964	*GALC*	galactosylceramidase	-1.36	0.036400
A_33_P3659876	*NCAPG2*	non-SMC condensin II complex, subunit G2	-1.36	0.038672
A_33_P3280646	*CASR*	calcium-sensing receptor	-1.36	0.038424
A_33_P3324114	*TUSC7*	tumor suppressor candidate 7 (non-protein coding)	-1.36	0.035256
A_23_P20660	*DIRAS2*	DIRAS family, GTP-binding RAS-like 2	-1.35	0.025878
A_33_P3365072	*LCE6A*	late cornified envelope 6A	-1.35	0.036795
A_33_P3382867	*LOC389834*	ankyrin repeat domain 57 pseudogene	-1.35	0.048472
A_23_P108501	*EPHA4*	EPH receptor A4	-1.35	0.042359
A_23_P11081	*AKAP4*	A kinase (PRKA) anchor protein 4	-1.35	0.028589
A_23_P115011	*ADAMTSL4*	ADAMTS-like 4	-1.35	0.044802
A_23_P128974	*BATF*	basic leucine zipper transcription factor, ATF-like	-1.35	0.047238
A_33_P3539345	*MYO6*	myosin VI	-1.34	0.045607
A_23_P63972	*DOC2GP*	double C2-like domains, gamma, pseudogene	-1.34	0.029908
A_24_P334248	*PLCH1*	phospholipase C, eta 1	-1.34	0.047807
A_33_P3229572	*RASA4B*	RAS p21 protein activator 4B	-1.33	0.046464
A_33_P3411980	*ABI3BP*	ABI family, member 3 (NESH) binding protein	-1.33	0.028699

A_32_P42018	*CCDC179*	coiled-coil domain containing 179	-1.33	0.049584
A_23_P254081	*LIAS*	lipoic acid synthetase	-1.33	0.038506
A_33_P3392447	*BCL10*	B-cell CLL/lymphoma 10	-1.33	0.048686
A_32_P208350	*TDRD9*	tudor domain containing 9	-1.33	0.042753
A_23_P32308	*ATP4B*	ATPase, H+/K+ exchanging, beta polypeptide	-1.33	0.039197
A_33_P3261914	*LOC102724738*	tricarboxylate transport protein, mitochondrial-like	-1.33	0.040443
A_32_P764462	*FLJ40536*	FLJ40536 protein	-1.33	0.034996
A_32_P27479	*NLRP11*	NLR family, pyrin domain containing 11	-1.33	0.047706
A_23_P152107	*UBE2I*	ubiquitin-conjugating enzyme E2I	-1.33	0.036186
A_24_P36299	*ARHGAP35*	Rho GTPase activating protein 35	-1.32	0.043935
A_23_P86411	*MYO3A*	myosin IIIA	-1.32	0.039431
A_33_P3415062	*KIAA1671*	KIAA1671	-1.32	0.041962
A_33_P3262133	*GFM1*	G elongation factor, mitochondrial 1	-1.32	0.042899
A_32_P125832	*GATA6-AS1*	GATA6 antisense RNA 1 (head to head)	-1.31	0.048950
A_24_P760960	*SKINTL*	Skint-like, pseudogene	-1.30	0.046016
A_32_P14582	*C3P1*	complement component 3 precursor pseudogene	-1.30	0.048493
A_33_P3362321	*FHL2*	four and a half LIM domains 2	-1.30	0.040817
A_23_P27128	*KLHL10*	kelch-like family member 10	-1.30	0.046530
A_19_P00809563	*METTL15*	methyltransferase like 15	-1.30	0.044053
A_33_P3254590	*HNRNPKP3*	heterogeneous nuclear ribonucleoprotein K pseudogene 3	-1.29	0.049926
A_33_P3789894	*SNORA75*	small nucleolar RNA, H/ACA box 75	-1.29	0.048256
A_23_P4074	*WDR16*	WD repeat domain 16	-1.28	0.049083
A_23_P29655	*C3orf14*	chromosome 3 open reading frame 14	1.29	0.047133
A_24_P648176	*LYRM5*	LYR motif containing 5	1.29	0.042628
A_33_P3399433	*C20orf27*	chromosome 20 open reading frame 27	1.31	0.046378
A_24_P15621	*SLC6A10P*	solute carrier family 6 (neurotransmitter transporter), member 10, pseudogene	1.31	0.040727
A_24_P250499	*RPRD1B*	regulation of nuclear pre-mRNA domain containing 1B	1.31	0.042453
A_23_P78849	*SYT5*	synaptotagmin V	1.31	0.048870
A_32_P49616	*EEF1B2*	eukaryotic translation elongation factor 1 beta 2	1.31	0.047337
A_23_P36464	*ASUN*	asunder spermatogenesis regulator	1.32	0.038428
A_33_P3589722	*ATM*	ataxia telangiectasia mutated	1.32	0.037776
A_23_P206724	*MT1E*	metallothionein 1E	1.32	0.042064
A_23_P63908	*TRUB1*	TruB pseudouridine (psi) synthase family member 1	1.33	0.033250
A_33_P3367994	*TAF8*	TAF8 RNA polymerase II, TATA box binding protein (TBP)-associated factor, 43kDa	1.33	0.041022
A_33_P3258141	*CHUK*	conserved helix-loop-helix ubiquitous kinase	1.33	0.043656
A_23_P421221	*R3HCC1*	R3H domain and coiled-coil containing 1	1.33	0.048567
A_32_P216548	*LDLRAP1*	low density lipoprotein receptor adaptor protein 1	1.34	0.040252
A_23_P109345	*PTTG1IP*	pituitary tumor-transforming 1 interacting protein	1.34	0.035374
A_23_P323751	*FAM83D*	family with sequence similarity 83, member D	1.34	0.035297
A_24_P116871	*TXNL4A*	thioredoxin-like 4A	1.34	0.047448
A_33_P3220390	*WTAP*	Wilms tumor 1 associated protein	1.34	0.048569
A_23_P81717	*FRMD1*	FERM domain containing 1	1.34	0.032671
A_33_P3407675	*TIPRL*	TOR signaling pathway regulator	1.34	0.026895
A_33_P3261418	*CACNA1G*	calcium channel, voltage-dependent, T type, alpha 1G subunit	1.34	0.047314

A_23_P4353	*WSB1*	WD repeat and SOCS box containing 1	1.34	0.038095
A_23_P359647	*NFAT5*	nuclear factor of activated T-cells 5, tonicity-responsive	1.35	0.040090
A_24_P196117	*GTF2H5*	general transcription factor IIH, polypeptide 5	1.35	0.044561
A_23_P349127	*CDAN1*	codanin 1	1.35	0.040678
A_23_P79661	*CCDC93*	coiled-coil domain containing 93	1.35	0.040443
A_23_P50000	*FAM57A*	family with sequence similarity 57, member A	1.36	0.047950
A_23_P24515	*ACAT1*	acetyl-CoA acetyltransferase 1	1.36	0.045724
A_23_P391164	*ZNF512*	zinc finger protein 512	1.36	0.035267
A_33_P3320082	*NFIB*	nuclear factor I/B	1.37	0.045427
A_23_P215875	*DCAF13*	DDB1 and CUL4 associated factor 13	1.37	0.042540
A_23_P43726	*NUP160*	nucleoporin 160kDa	1.37	0.023762
A_23_P24709	*OSBP*	oxysterol binding protein	1.37	0.029708
A_23_P306105	*GALNT1*	polypeptide N-acetylgalactosaminyltransferase 1	1.37	0.049163
A_23_P11507	*ZZZ3*	zinc finger, ZZ-type containing 3	1.38	0.033241
A_23_P253421	*AUP1*	ancient ubiquitous protein 1	1.38	0.032740
A_23_P114414	*LONRF3*	LON peptidase N-terminal domain and ring finger 3	1.38	0.027589
A_33_P3359413	*SGTA*	small glutamine-rich tetratricopeptide repeat (TPR)-containing, alpha	1.38	0.035410
A_23_P151267	*LIMA1*	LIM domain and actin binding 1	1.38	0.029977
A_23_P120744	*MCM3AP*	minichromosome maintenance complex component 3 associated protein	1.39	0.024999
A_23_P37914	*SLC5A11*	solute carrier family 5 (sodium/inositol cotransporter), member 11	1.39	0.031895
A_24_P419120	*BICD2*	bicaudal D homolog 2 (Drosophila)	1.39	0.030189
A_23_P161004	*NR5A2*	nuclear receptor subfamily 5, group A, member 2	1.39	0.020016
A_23_P143016	*ARID5A*	AT rich interactive domain 5A (MRF1-like)	1.39	0.049395
A_24_P151	*KCNAB2*	potassium voltage-gated channel, shaker-related subfamily, beta member 2	1.39	0.032905
A_23_P96087	*H1FX*	H1 histone family, member X	1.40	0.030524
A_33_P3369311	*BBX*	bobby sox homolog (Drosophila)	1.40	0.048857
A_32_P209989	*MRPL46*	mitochondrial ribosomal protein L46	1.40	0.017950
A_23_P160503	*GLRX2*	glutaredoxin 2	1.40	0.036084
A_23_P123539	*PPP2R2A*	protein phosphatase 2, regulatory subunit B, alpha	1.40	0.031739
A_23_P54900	*UBN1*	ubinuclein 1	1.40	0.049363
A_23_P332042	*RECQL5*	RecQ protein-like 5	1.41	0.043232
A_23_P89589	*PER1*	period circadian clock 1	1.41	0.027599
A_24_P639505	*STX6*	syntaxin 6	1.41	0.048226
A_23_P399078	*TIMP3*	TIMP metallopeptidase inhibitor 3	1.41	0.042372
A_24_P42501	*ACOT9*	acyl-CoA thioesterase 9	1.41	0.039376
A_24_P342829	*SLC16A14*	solute carrier family 16, member 14	1.41	0.027049
A_33_P3264577	*DCTN1*	dynactin 1	1.42	0.046513
A_23_P256716	*SMARCA5*	SWI/SNF related, matrix associated, actin dependent regulator of chromatin, subfamily a, member 5	1.42	0.024692
A_32_P62571	*RBM8A*	RNA binding motif protein 8A	1.42	0.047773
A_23_P3681	*NETO2*	neuropilin (NRP) and tolloid (TLL)-like 2	1.42	0.021770
A_33_P3238250	*F11R*	F11 receptor	1.42	0.018728
A_23_P423695	*MXD4*	MAX dimerization protein 4	1.42	0.030375
A_23_P121250	*EIF4A2*	eukaryotic translation initiation factor 4A2	1.42	0.027347

A_23_P153050	*ALKBH5*	alkB, alkylation repair homolog 5 (E. coli)	1.43	0.028278
A_23_P118888	*PAFAH1B1*	platelet-activating factor acetylhydrolase 1b, regulatory subunit 1 (45kDa)	1.43	0.024830
A_23_P204016	*CACNB3*	calcium channel, voltage-dependent, beta 3 subunit	1.43	0.041617
A_23_P258689	*HEATR2*	HEAT repeat containing 2	1.43	0.015407
A_23_P213883	*NIPBL*	Nipped-B homolog (Drosophila)	1.43	0.036997
A_23_P105664	*CCDC59*	coiled-coil domain containing 59	1.43	0.021938
A_33_P3344673	*PPP1R37*	protein phosphatase 1, regulatory subunit 37	1.43	0.026604
A_23_P163235	*CKMT1A*	creatine kinase, mitochondrial 1A	1.44	0.047485
A_33_P3386726	*PI4KB*	phosphatidylinositol 4-kinase, catalytic, beta	1.44	0.027400
A_23_P376557	*MMP25*	matrix metallopeptidase 25	1.44	0.035839
A_23_P71867	*IL11RA*	interleukin 11 receptor, alpha	1.44	0.026815
A_23_P258418	*TNIP2*	TNFAIP3 interacting protein 2	1.44	0.034680
A_33_P3651948	*NEO1*	neogenin 1	1.44	0.042268
A_23_P203137	*UBE4A*	ubiquitination factor E4A	1.44	0.016774
A_23_P255785	*NCK1*	NCK adaptor protein 1	1.45	0.022336
A_33_P3372466	*TECR*	trans-2,3-enoyl-CoA reductase	1.45	0.022709
A_24_P227585	*GPALPP1*	GPALPP motifs containing 1	1.45	0.049859
A_23_P170587	*SMYD2*	SET and MYND domain containing 2	1.45	0.034270
A_23_P92860	*CCNO*	cyclin O	1.45	0.021917
A_33_P3229196	*CD151*	CD151 molecule (Raph blood group)	1.45	0.046401
A_23_P160177	*ATP1A4*	ATPase, Na+/K+ transporting, alpha 4 polypeptide	1.45	0.037294
A_23_P148372	*CSTF2*	cleavage stimulation factor, 3' pre-RNA, subunit 2, 64kDa	1.45	0.041530
A_23_P136635	*AP3B1*	adaptor-related protein complex 3, beta 1 subunit	1.45	0.041922
A_23_P78037	*CCL7*	chemokine (C-C motif) ligand 7	1.46	0.020324
A_23_P164011	*SOX15*	SRY (sex determining region Y)-box 15	1.46	0.030844
A_23_P501996	*UBE2V1*	ubiquitin-conjugating enzyme E2 variant 1	1.46	0.044866
A_23_P205830	*BTBD1*	BTB (POZ) domain containing 1	1.46	0.034324
A_33_P3228564	*DOK3*	docking protein 3	1.46	0.034747
A_33_P3219260	*PDZD11*	PDZ domain containing 11	1.46	0.012889
A_33_P3372840	*CXCL12*	chemokine (C-X-C motif) ligand 12	1.47	0.025765
A_23_P29057	*KCNJ6*	potassium inwardly-rectifying channel, subfamily J, member 6	1.47	0.032815
A_24_P168416	*PRDX2*	peroxiredoxin 2	1.47	0.041036
A_23_P211797	*OPA1*	optic atrophy 1 (autosomal dominant)	1.47	0.034792
A_23_P34788	*KIF2C*	kinesin family member 2C	1.47	0.023913
A_23_P74526	*PPIE*	peptidylprolyl isomerase E (cyclophilin E)	1.47	0.047971
A_24_P108451	*GPI*	glucose-6-phosphate isomerase	1.47	0.020353
A_23_P206920	*MYH11*	myosin, heavy chain 11, smooth muscle	1.47	0.031791
A_32_P41604	*F5*	coagulation factor V (proaccelerin, labile factor)	1.47	0.046101
A_33_P3251148	*TSPO*	translocator protein (18kDa)	1.47	0.022814
A_23_P100203	*HSBP1*	heat shock factor binding protein 1	1.48	0.030861
A_32_P18250	*FUNDC2P2*	FUN14 domain containing 2 pseudogene 2	1.48	0.049079
A_33_P3249696	*DLGAP1-AS5*	DLGAP1 antisense RNA 5	1.48	0.044653
A_23_P16157	*KHSRP*	KH-type splicing regulatory protein	1.48	0.028626
A_23_P111311	*AKAP12*	A kinase (PRKA) anchor protein 12	1.49	0.033939

A_23_P91468	*PSMA7*	proteasome (prosome, macropain) subunit, alpha type, 7	1.49	0.025944
A_24_P111134	*POMT2*	protein-O-mannosyltransferase 2	1.49	0.047732
A_23_P111041	*HIST1H2BI*	histone cluster 1, H2bi	1.49	0.020867
A_23_P205007	*IPO5*	importin 5	1.49	0.029289
A_23_P368101	*SAP30L*	SAP30-like	1.49	0.027556
A_23_P215419	*ICA1*	islet cell autoantigen 1, 69kDa	1.49	0.046691
A_23_P362183	*ANKS6*	ankyrin repeat and sterile alpha motif domain containing 6	1.49	0.035232
A_23_P78771	*NUCB1*	nucleobindin 1	1.49	0.039221
A_23_P118690	**ZNF207*	zinc finger protein 207	1.49	0.021259
A_33_P3414157	*MLPH*	melanophilin	1.50	0.048818
A_23_P42144	*PEX6*	peroxisomal biogenesis factor 6	1.50	0.048547
A_23_P216468	*SLC1A1*	solute carrier family 1 (neuronal/epithelial high affinity glutamate transporter, system Xag), member 1	1.50	0.049732
A_23_P99027	*PTPN11*	protein tyrosine phosphatase, non-receptor type 11	1.50	0.031799
A_33_P3247489	*C11orf85*	chromosome 11 open reading frame 85	1.50	0.025231
A_33_P3343145	*MAP1B*	microtubule-associated protein 1B	1.50	0.008345
A_24_P185314	*NAPG*	N-ethylmaleimide-sensitive factor attachment protein, gamma	1.50	0.029533
A_23_P34915	*ATF3*	activating transcription factor 3	1.51	0.010710
A_24_P79617	*KIAA0040*	KIAA0040	1.51	0.012346
A_23_P100912	*GIT1*	G protein-coupled receptor kinase interacting ArfGAP 1	1.51	0.025546
A_23_P41066	*RASSF1*	Ras association (RalGDS/AF-6) domain family member 1	1.51	0.034220
A_23_P129695	*VASN*	vasorin	1.51	0.048817
A_24_P342511	*STMN1*	stathmin 1	1.51	0.048144
A_24_P365025	*SPAG9*	sperm associated antigen 9	1.51	0.035403
A_23_P384517	*GYG1*	glycogenin 1	1.51	0.029074
A_33_P3389942	*NHP2*	NHP2 ribonucleoprotein	1.51	0.034888
A_33_P3257910	*C3orf80*	chromosome 3 open reading frame 80	1.52	0.036124
A_23_P56529	*AAMP*	angio-associated, migratory cell protein	1.52	0.015317
A_23_P98282	*SPTBN2*	spectrin, beta, non-erythrocytic 2	1.52	0.036355
A_24_P385134	*SCD5*	stearoyl-CoA desaturase 5	1.52	0.049226
A_33_P3380807	*TLR9*	toll-like receptor 9	1.52	0.026378
A_24_P299996	*SHQ1*	SHQ1, H/ACA ribonucleoprotein assembly factor	1.52	0.048473
A_23_P120933	*ATF4*	activating transcription factor 4	1.53	0.041223
A_24_P288424	*SLMO2*	slowmo homolog 2 (Drosophila)	1.53	0.048023
A_23_P18196	*RFC4*	replication factor C (activator 1) 4, 37kDa	1.53	0.025546
A_23_P213562	*F2R*	coagulation factor II (thrombin) receptor	1.53	0.045097
A_23_P214026	*FBN2*	fibrillin 2	1.53	0.044612
A_23_P76731	*MOK*	MOK protein kinase	1.53	0.048499
A_23_P126593	*S100A11*	S100 calcium binding protein A11	1.53	0.045823
A_23_P356070	*FLT4*	fms-related tyrosine kinase 4	1.53	0.028363
A_24_P307580	*HTATIP2*	HIV-1 Tat interactive protein 2, 30kDa	1.53	0.033976
A_23_P129209	*IDH2*	isocitrate dehydrogenase 2 (NADP+), mitochondrial	1.53	0.030217
A_33_P3345210	*TLCD2*	TLC domain containing 2	1.53	0.024773
A_24_P294719	*FAF2*	Fas associated factor family member 2	1.53	0.037976
A_23_P165891	*TAF1B*	TATA box binding protein (TBP)-associated factor, RNA polymerase I, B, 63kDa	1.53	0.023680

A_24_P57700	*ZHX3*	zinc fingers and homeoboxes 3	1.54	0.042100
A_23_P5339	*TMEM177*	transmembrane protein 177	1.54	0.022778
A_23_P324718	*SYNJ1*	synaptojanin 1	1.54	0.016761
A_23_P58521	*ERCC8*	excision repair cross-complementation group 8	1.54	0.044009
A_23_P11214	*NKRF*	NFKB repressing factor	1.54	0.027128
A_23_P37441	*B2M*	beta-2-microglobulin	1.54	0.014298
A_32_P25273	*HSPD1*	heat shock 60kDa protein 1 (chaperonin)	1.54	0.046551
A_33_P3416037	*FAM96A*	family with sequence similarity 96, member A	1.54	0.048355
A_23_P136671	*UGT2B7*	UDP glucuronosyltransferase 2 family, polypeptide B7	1.54	0.010086
A_24_P101921	*EXOC5*	exocyst complex component 5	1.54	0.017079
A_24_P419309	*SNRNP40*	small nuclear ribonucleoprotein 40kDa (U5)	1.55	0.049689
A_23_P155907	*METAP1*	methionyl aminopeptidase 1	1.55	0.033111
A_23_P36266	*PDHX*	pyruvate dehydrogenase complex, component X	1.55	0.025426
A_23_P61551	*CD2BP2*	CD2 (cytoplasmic tail) binding protein 2	1.55	0.049337
A_23_P118306	*DNAJA3*	DnaJ (Hsp40) homolog, subfamily A, member 3	1.55	0.039260
A_33_P3236703	*ANKRD18B*	ankyrin repeat domain 18B	1.55	0.023244
A_23_P76109	*RILPL2*	Rab interacting lysosomal protein-like 2	1.55	0.038434
A_24_P417596	*FLYWCH2*	FLYWCH family member 2	1.55	0.030468
A_23_P23575	*SLC39A1*	solute carrier family 39 (zinc transporter), member 1	1.55	0.032864
A_33_P3364379	*YIF1A*	Yip1 interacting factor homolog A (S. cerevisiae)	1.55	0.019786
A_33_P3239587	*MXRA7*	matrix-remodelling associated 7	1.56	0.044817
A_23_P143414	*ROMO1*	reactive oxygen species modulator 1	1.56	0.039088
A_23_P152284	*SNRNP25*	small nuclear ribonucleoprotein 25kDa (U11/U12)	1.56	0.042591
A_33_P3349469	*ATAD3A*	ATPase family, AAA domain containing 3A	1.56	0.012497
A_24_P226116	*NAA15*	N(alpha)-acetyltransferase 15, NatA auxiliary subunit	1.56	0.036246
A_24_P398940	*CASC4*	cancer susceptibility candidate 4	1.56	0.047757
A_33_P3310189	*ADRB1*	adrenoceptor beta 1	1.57	0.037550
A_23_P80040	*PROCR*	protein C receptor, endothelial	1.57	0.021163
A_23_P53788	*MTIF3*	mitochondrial translational initiation factor 3	1.57	0.049587
A_24_P25080	*CBWD5*	COBW domain containing 5	1.57	0.029717
A_24_P239731	*B4GALT5*	UDP-Gal:betaGlcNAc beta 1,4- galactosyltransferase, polypeptide 5	1.57	0.020344
A_24_P287826	*RFWD3*	ring finger and WD repeat domain 3	1.58	0.039480
A_23_P53267	*RSRC2*	arginine/serine-rich coiled-coil 2	1.58	0.018598
A_24_P400473	*SLC25A44*	solute carrier family 25, member 44	1.58	0.038904
A_23_P78438	*ELP2*	elongator acetyltransferase complex subunit 2	1.58	0.029683
A_23_P155332	*PCNP*	PEST proteolytic signal containing nuclear protein	1.58	0.018794
A_23_P254271	*TUBB6*	tubulin, beta 6 class V	1.58	0.030736
A_24_P414719	*NFYA*	nuclear transcription factor Y, alpha	1.58	0.044136
A_23_P32938	*DDX10*	DEAD (Asp-Glu-Ala-Asp) box polypeptide 10	1.59	0.008955
A_23_P411723	*PLAG1*	pleiomorphic adenoma gene 1	1.59	0.037400
A_19_P00317789	*LOC93622*	Morf4 family associated protein 1-like 1 pseudogene	1.59	0.045638
A_23_P72961	*PRPS1*	phosphoribosyl pyrophosphate synthetase 1	1.59	0.007430
A_23_P68211	*SPR*	sepiapterin reductase (7,8-dihydrobiopterin:NADP+ oxidoreductase)	1.59	0.020090
A_23_P111005	*YIPF3*	Yip1 domain family, member 3	1.59	0.021608

A_24_P36847	*DHX9*	DEAH (Asp-Glu-Ala-His) box helicase 9	1.59	0.041482
A_33_P3246007	*APOA1BP*	apolipoprotein A-I binding protein	1.59	0.047596
A_23_P216489	*GNE*	glucosamine (UDP-N-acetyl)-2-epimerase/N-acetylmannosamine kinase	1.59	0.031227
A_23_P359616	*RPF1*	ribosome production factor 1 homolog (S. cerevisiae)	1.60	0.039092
A_23_P65427	*PSME2*	proteasome (prosome, macropain) activator subunit 2 (PA28 beta)	1.60	0.038197
A_23_P380181	*LMO4*	LIM domain only 4	1.60	0.022169
A_23_P64770	*DDX23*	DEAD (Asp-Glu-Ala-Asp) box polypeptide 23	1.60	0.004596
A_23_P114405	*MORF4L2*	mortality factor 4 like 2	1.60	0.007146
A_32_P168247	*COX6A1*	cytochrome c oxidase subunit VIa polypeptide 1	1.60	0.035764
A_23_P44643	*ANAPC7*	anaphase promoting complex subunit 7	1.60	0.017498
A_23_P48596	*RNASE1*	ribonuclease, RNase A family, 1 (pancreatic)	1.60	0.015082
A_23_P171143	*TSPAN6*	tetraspanin 6	1.61	0.034186
A_23_P134125	*MAP3K5*	mitogen-activated protein kinase kinase kinase 5	1.61	0.030899
A_24_P152635	*TMX2*	thioredoxin-related transmembrane protein 2	1.61	0.041807
A_23_P162589	*VDR*	vitamin D (1,25- dihydroxyvitamin D3) receptor	1.61	0.006337
A_24_P188071	*TUBA1C*	tubulin, alpha 1c	1.61	0.012864
A_24_P143189	*TMSB4X*	thymosin beta 4, X-linked	1.61	0.010947
A_33_P3378047	*SESTD1*	SEC14 and spectrin domains 1	1.61	0.032117
A_23_P77776	*SRSF2*	serine/arginine-rich splicing factor 2	1.62	0.049890
A_33_P3292540	*CDKN2C*	cyclin-dependent kinase inhibitor 2C (p18, inhibits CDK4)	1.62	0.045524
A_23_P152655	*ICAM2*	intercellular adhesion molecule 2	1.62	0.046634
A_33_P3268313	*PGAM2*	phosphoglycerate mutase 2 (muscle)	1.62	0.031809
A_24_P294124	*SERTAD2*	SERTA domain containing 2	1.62	0.043988
A_23_P819	*ISG15*	ISG15 ubiquitin-like modifier	1.63	0.030831
A_33_P3313401	*CYCS*	cytochrome c, somatic	1.63	0.039341
A_23_P59294	*RREB1*	ras responsive element binding protein 1	1.63	0.038040
A_23_P169112	*CPSF1*	cleavage and polyadenylation specific factor 1, 160kDa	1.63	0.010473
A_24_P898945	*FAM210A*	family with sequence similarity 210, member A	1.64	0.028714
A_33_P3318465	*SUMO1*	small ubiquitin-like modifier 1	1.64	0.018298
A_33_P3335124	*RPS2*	ribosomal protein S2	1.64	0.031171
A_33_P3341499	*WNT5A*	wingless-type MMTV integration site family, member 5A	1.64	0.017232
A_32_P229746	*DNAJB6*	DnaJ (Hsp40) homolog, subfamily B, member 6	1.64	0.011217
A_24_P221366	*RPS15A*	ribosomal protein S15a	1.64	0.020231
A_23_P32115	*LCN12*	lipocalin 12	1.64	0.039788
A_23_P60324	*UBAC1*	UBA domain containing 1	1.64	0.018601
A_33_P3315021	*RPL23AP7*	ribosomal protein L23a pseudogene 7	1.64	0.035021
A_24_P360269	*RNASET2*	ribonuclease T2	1.64	0.034320
A_23_P138835	*CAPN1*	calpain 1, (mu/I) large subunit	1.65	0.021178
A_23_P145089	*HSP90AB1*	heat shock protein 90kDa alpha (cytosolic), class B member 1	1.65	0.018859
A_23_P145817	*MALSU1*	mitochondrial assembly of ribosomal large subunit 1	1.65	0.022588
A_33_P3371493	*TOP1*	topoisomerase (DNA) I	1.65	0.044826
A_23_P148015	*AXIN2*	axin 2	1.65	0.025296
A_23_P164047	*MMD*	monocyte to macrophage differentiation-associated	1.65	0.014904
A_23_P259344	*CECR6*	cat eye syndrome chromosome region, candidate 6	1.65	0.035769

A_33_P3283320	*OR6F1*	olfactory receptor, family 6, subfamily F, member 1	1.65	0.033952
A_23_P218997	*PDCD6*	programmed cell death 6	1.66	0.026849
A_23_P3532	*LITAF*	lipopolysaccharide-induced TNF factor	1.66	0.022318
A_23_P103628	*HEATR1*	HEAT repeat containing 1	1.66	0.028199
A_33_P3240722	*CNKSR2*	connector enhancer of kinase suppressor of Ras 2	1.66	0.009383
A_23_P209394	*CFLAR*	CASP8 and FADD-like apoptosis regulator	1.67	0.011177
A_23_P81399	*SQSTM1*	sequestosome 1	1.67	0.033481
A_24_P283928	*NABP2*	nucleic acid binding protein 2	1.67	0.030695
A_23_P40453	*CBR3*	carbonyl reductase 3	1.67	0.004362
A_24_P304051	*GSTO1*	glutathione S-transferase omega 1	1.67	0.007109
A_24_P237661	*NCOR1*	nuclear receptor corepressor 1	1.68	0.026656
A_24_P942211	*SLC35E2*	solute carrier family 35, member E2	1.68	0.019780
A_23_P152181	*POLR3E*	polymerase (RNA) III (DNA directed) polypeptide E (80kD)	1.68	0.037740
A_23_P43613	*NDUFB6*	NADH dehydrogenase (ubiquinone) 1 beta subcomplex, 6, 17kDa	1.68	0.022423
A_33_P3348313	*ELP6*	elongator acetyltransferase complex subunit 6	1.69	0.033899
A_33_P3224803	*NCF1*	neutrophil cytosolic factor 1	1.69	0.036415
A_23_P60016	*PTTG3P*	pituitary tumor-transforming 3, pseudogene	1.69	0.028950
A_33_P3289371	*PHACTR3*	phosphatase and actin regulator 3	1.69	0.013419
A_24_P98109	*SNX10*	sorting nexin 10	1.69	0.033142
A_24_P91310	*PSMC4*	proteasome (prosome, macropain) 26S subunit, ATPase, 4	1.69	0.026190
A_23_P30495	*HMGCR*	3-hydroxy-3-methylglutaryl-CoA reductase	1.70	0.039416
A_23_P25835	*FNTB*	farnesyltransferase, CAAX box, beta	1.70	0.018979
A_19_P00800467	*CLEC2D*	C-type lectin domain family 2, member D	1.70	0.030994
A_23_P82929	*NOV*	nephroblastoma overexpressed	1.70	0.017299
A_23_P97632	*EPRS*	glutamyl-prolyl-tRNA synthetase	1.71	0.005043
A_33_P3287815	*DDX21*	DEAD (Asp-Glu-Ala-Asp) box helicase 21	1.71	0.012486
A_23_P103661	*YY1AP1*	YY1 associated protein 1	1.71	0.006619
A_23_P39110	*RUVBL2*	RuvB-like AAA ATPase 2	1.71	0.011557
A_23_P432360	*PMM2*	phosphomannomutase 2	1.71	0.033739
A_33_P3220352	*CNPY1*	canopy FGF signaling regulator 1	1.71	0.029979
A_23_P71752	*ZFAND5*	zinc finger, AN1-type domain 5	1.72	0.034885
A_23_P370434	*C1QBP*	complement component 1, q subcomponent binding protein	1.72	0.017953
A_23_P501887	*DHPS*	deoxyhypusine synthase	1.72	0.049785
A_32_P57702	*LOC641746*	glycine cleavage system protein H (aminomethyl carrier) pseudogene	1.73	0.006938
A_23_P5325	*ERCC3*	excision repair cross-complementation group 3	1.73	0.031944
A_23_P154771	*DUSP15*	dual specificity phosphatase 15	1.73	0.012641
A_23_P207399	*NBR1*	neighbor of BRCA1 gene 1	1.74	0.040125
A_23_P103905	*UFC1*	ubiquitin-fold modifier conjugating enzyme 1	1.74	0.029481
A_23_P413634	*ZNF329*	zinc finger protein 329	1.74	0.023769
A_23_P209904	*GPC1*	glypican 1	1.74	0.048500
A_24_P9671	*DNAJA1*	DnaJ (Hsp40) homolog, subfamily A, member 1	1.74	0.048688
A_23_P52101	*CYB5R1*	cytochrome b5 reductase 1	1.74	0.042355
A_23_P161615	*POLA2*	polymerase (DNA directed), alpha 2, accessory subunit	1.74	0.014046
A_24_P67308	*RPL19P12*	ribosomal protein L19 pseudogene 12	1.75	0.042859

A_32_P81357	*FAHD2A*	fumarylacetoacetate hydrolase domain containing 2A	1.75	0.044345
A_23_P122624	*LTV1*	LTV1 ribosome biogenesis factor	1.75	0.032000
A_23_P87742	*IFFO1*	intermediate filament family orphan 1	1.75	0.047666
A_33_P3276323	*RAD21-AS1*	RAD21 antisense RNA 1	1.76	0.015123
A_32_P171328	*UBE2S*	ubiquitin-conjugating enzyme E2S	1.76	0.036697
A_23_P79999	*ENTPD6*	ectonucleoside triphosphate diphosphohydrolase 6 (putative)	1.76	0.017872
A_23_P212475	*SHISA5*	shisa family member 5	1.76	0.024536
A_23_P53541	*CHD4*	chromodomain helicase DNA binding protein 4	1.76	0.042721
A_33_P3316410	*FAM66A*	family with sequence similarity 66, member A	1.77	0.015008
A_33_P3248519	*SMC4*	structural maintenance of chromosomes 4	1.77	0.026767
A_33_P3286278	*GRN*	granulin	1.77	0.010646
A_23_P401472	*CHRM3*	cholinergic receptor, muscarinic 3	1.77	0.038527
A_23_P90062	*DNAJB1*	DnaJ (Hsp40) homolog, subfamily B, member 1	1.77	0.031504
A_24_P366526	*SYNGR2*	synaptogyrin 2	1.77	0.007467
A_23_P162874	*HSP90AA1*	heat shock protein 90kDa alpha (cytosolic), class A member 1	1.78	0.023912
A_33_P3401301	*RPL39*	ribosomal protein L39	1.78	0.005087
A_23_P344421	*ROBO4*	roundabout, axon guidance receptor, homolog 4 (Drosophila)	1.78	0.029225
A_23_P80773	*SRPRB*	signal recognition particle receptor, B subunit	1.78	0.002646
A_33_P3321657	*HSPG2*	heparan sulfate proteoglycan 2	1.78	0.012265
A_24_P406714	*MLLT10*	myeloid/lymphoid or mixed-lineage leukemia (trithorax homolog, Drosophila); translocated to, 10	1.78	0.007633
A_24_P72479	*ARPC1A*	actin related protein 2/3 complex, subunit 1A, 41kDa	1.78	0.029081
A_33_P3383226	*GP9*	glycoprotein IX (platelet)	1.79	0.039962
A_33_P3263625	*DUSP8*	dual specificity phosphatase 8	1.79	0.035803
A_24_P336957	*HTRA2*	HtrA serine peptidase 2	1.79	0.026789
A_24_P176714	*B9D1*	B9 protein domain 1	1.79	0.035645
A_23_P160631	*CCT3*	chaperonin containing TCP1, subunit 3 (gamma)	1.79	0.030371
A_23_P130182	*AURKB*	aurora kinase B	1.79	0.043362
A_33_P3226357	*FOXE1*	forkhead box E1 (thyroid transcription factor 2)	1.80	0.033893
A_23_P133755	*PREP*	prolyl endopeptidase	1.80	0.034765
A_23_P78134	*EMC6*	ER membrane protein complex subunit 6	1.80	0.025858
A_32_P109572	*HNRNPL*	heterogeneous nuclear ribonucleoprotein L	1.80	0.005242
A_24_P221575	*RUFY3*	RUN and FYVE domain containing 3	1.80	0.018550
A_33_P3354137	*MAP4*	microtubule-associated protein 4	1.80	0.049861
A_23_P212511	*TTC14*	tetratricopeptide repeat domain 14	1.81	0.041542
A_24_P942517	*TMX4*	thioredoxin-related transmembrane protein 4	1.81	0.035140
A_32_P114896	*PTGES3*	prostaglandin E synthase 3 (cytosolic)	1.81	0.023729
A_23_P38482	*PHF23*	PHD finger protein 23	1.81	0.008160
A_32_P114574	*CACYBP*	calcyclin binding protein	1.81	0.045089
A_23_P159671	*PHKA2*	phosphorylase kinase, alpha 2 (liver)	1.82	0.003563
A_23_P500998	*HOXA9*	homeobox A9	1.82	0.025068
A_24_P253755	*PIGL*	phosphatidylinositol glycan anchor biosynthesis, class L	1.82	0.028760
A_23_P137391	*ENO1*	enolase 1, (alpha)	1.82	0.033440
A_33_P3288754	*C19orf48*	chromosome 19 open reading frame 48	1.83	0.039196
A_19_P00805840	*ZNF37BP*	zinc finger protein 37B, pseudogene	1.83	0.033086

A_33_P3291097	*BCAM*	basal cell adhesion molecule (Lutheran blood group)	1.83	0.028133
A_24_P26554	*LYRM4*	LYR motif containing 4	1.83	0.032385
A_33_P3239347	*NKX3-1*	NK3 homeobox 1	1.83	0.006638
A_32_P216566	*RIPPLY2*	ripply transcriptional repressor 2	1.83	0.030611
A_23_P320739	*MEF2C*	myocyte enhancer factor 2C	1.83	0.016582
A_23_P82296	*GNB2*	guanine nucleotide binding protein (G protein), beta polypeptide 2	1.83	0.023551
A_24_P338145	*STOML2*	stomatin (EPB72)-like 2	1.83	0.049261
A_23_P201386	*DDAH1*	dimethylarginine dimethylaminohydrolase 1	1.84	0.010142
A_23_P303548	*NOL4L*	nucleolar protein 4-like	1.84	0.022687
A_23_P154840	*SOD1*	superoxide dismutase 1, soluble	1.84	0.030142
A_33_P3268763	*TMUB1*	transmembrane and ubiquitin-like domain containing 1	1.85	0.013544
A_23_P19084	*HNRNPAB*	heterogeneous nuclear ribonucleoprotein A/B	1.86	0.042431
A_23_P56380	*ZC3H15*	zinc finger CCCH-type containing 15	1.86	0.022760
A_23_P84836	*NPEPPS*	aminopeptidase puromycin sensitive	1.86	0.015385
A_33_P3230876	*ALG11*	ALG11, alpha-1,2-mannosyltransferase	1.86	0.045458
A_33_P3319900	*PRG1*	p53-responsive gene 1	1.87	0.032023
A_23_P5089	*ATP5D*	ATP synthase, H+ transporting, mitochondrial F1 complex, delta subunit	1.87	0.012655
A_23_P359738	*EPC2*	enhancer of polycomb homolog 2 (Drosophila)	1.87	0.044443
A_33_P3400429	*UBE3A*	ubiquitin protein ligase E3A	1.87	0.006140
A_33_P3336878	*TMEM180*	transmembrane protein 180	1.87	0.030416
A_33_P3254801	*OGFR*	opioid growth factor receptor	1.87	0.047855
A_24_P23995	*RNF187*	ring finger protein 187	1.87	0.035944
A_23_P100676	*SMG6*	SMG6 nonsense mediated mRNA decay factor	1.87	0.038954
A_23_P318296	*PLEKHA8*	pleckstrin homology domain containing, family A (phosphoinositide binding specific) member 8	1.87	0.014668
A_23_P105900	*BTF3P11*	basic transcription factor 3 pseudogene 11	1.87	0.030418
A_33_P3405349	*C7orf43*	chromosome 7 open reading frame 43	1.88	0.047980
A_24_P30314	*SCYL1*	SCY1-like 1 (S. cerevisiae)	1.88	0.021691
A_23_P84872	*SECISBP2*	SECIS binding protein 2	1.88	0.025226
A_33_P3390758	*HSPA8*	heat shock 70kDa protein 8	1.88	0.013808
A_23_P63655	*ATP5C1*	ATP synthase, H+ transporting, mitochondrial F1 complex, gamma polypeptide 1	1.89	0.028075
A_32_P190416	*MAP7*	microtubule-associated protein 7	1.89	0.018042
A_24_P318593	*SCRN2*	secernin 2	1.90	0.024645
A_24_P324563	*ABHD17A*	abhydrolase domain containing 17A	1.90	0.047717
A_23_P93823	*RFC2*	replication factor C (activator 1) 2, 40kDa	1.90	0.020289
A_23_P64799	*AAAS*	achalasia, adrenocortical insufficiency, alacrimia	1.90	0.018536
A_23_P151075	*ARHGDIB*	Rho GDP dissociation inhibitor (GDI) beta	1.91	0.014430
A_23_P35989	*ALKBH3*	alkB, alkylation repair homolog 3 (E. coli)	1.91	0.036848
A_23_P12503	*NUP133*	nucleoporin 133kDa	1.91	0.026049
A_23_P202720	*SLC35C1*	solute carrier family 35 (GDP-fucose transporter), member C1	1.92	0.044928
A_33_P3264188	*OR2D3*	olfactory receptor, family 2, subfamily D, member 3	1.92	0.047448
A_23_P340263	*RNF175*	ring finger protein 175	1.92	0.024111
A_23_P321223	*PMCH*	pro-melanin-concentrating hormone	1.92	0.020525
A_23_P327069	*KIAA0232*	KIAA0232	1.92	0.041871

A_23_P31489	*URGCP*	upregulator of cell proliferation	1.93	0.036141
A_24_P56317	*MBNL2*	muscleblind-like splicing regulator 2	1.93	0.026090
A_23_P110811	*COX7C*	cytochrome c oxidase subunit VIIc	1.93	0.040292
A_23_P37654	*MGA*	MGA, MAX dimerization protein	1.94	0.047036
A_33_P3255706	*OR6K6*	olfactory receptor, family 6, subfamily K, member 6	1.94	0.011485
A_23_P57059	*STAU1*	staufen double-stranded RNA binding protein 1	1.95	0.013820
A_32_P80255	*DDX6*	DEAD (Asp-Glu-Ala-Asp) box helicase 6	1.95	0.020484
A_33_P3385477	*ATAD3B*	ATPase family, AAA domain containing 3B	1.96	0.046290
A_24_P380919	*HNRNPK*	heterogeneous nuclear ribonucleoprotein K	1.97	0.049367
A_33_P3258191	*C16orf91*	chromosome 16 open reading frame 91	1.97	0.019649
A_24_P134834	*RHBDD1*	rhomboid domain containing 1	1.98	0.007839
A_24_P270144	*CD63*	CD63 molecule	1.98	0.009551
A_33_P3328026	*CDK19*	cyclin-dependent kinase 19	1.99	0.020428
A_23_P304287	*PSMC2*	proteasome (prosome, macropain) 26S subunit, ATPase, 2	2.00	0.001092
A_33_P3421827	*HBZ*	hemoglobin, zeta	2.00	0.040419
A_24_P216087	*PAF1*	Paf1, RNA polymerase II associated factor, homolog (S. cerevisiae)	2.00	0.036841
A_24_P795371	*NR2F2-AS1*	NR2F2 antisense RNA 1	2.00	0.001085
A_33_P3405285	*GSG1*	germ cell associated 1	2.01	0.025828
A_23_P98310	*CSTF3*	cleavage stimulation factor, 3' pre-RNA, subunit 3, 77kDa	2.01	0.038654
A_32_P140898	*FOXN2*	forkhead box N2	2.01	0.049965
A_23_P163458	*EHD4*	EH-domain containing 4	2.02	0.030776
A_33_P3240543	*AGAP3*	ArfGAP with GTPase domain, ankyrin repeat and PH domain 3	2.02	0.015985
A_32_P220715	*MAP1LC3B*	microtubule-associated protein 1 light chain 3 beta	2.03	0.007657
A_23_P121282	*TMEM89*	transmembrane protein 89	2.03	0.049641
A_23_P256694	*MCM3AP-AS1*	MCM3AP antisense RNA 1	2.04	0.032341
A_23_P412932	*SPP2*	secreted phosphoprotein 2, 24kDa	2.04	0.048884
A_23_P75149	*SFXN4*	sideroflexin 4	2.04	0.023446
A_24_P369694	*PPP2R5C*	protein phosphatase 2, regulatory subunit B', gamma	2.04	0.029487
A_24_P212152	*MZT2B*	mitotic spindle organizing protein 2B	2.04	0.036853
A_24_P364296	*STX2*	syntaxin 2	2.05	0.045382
A_33_P3290677	*HELT*	helt bHLH transcription factor	2.05	0.032158
A_33_P3279640	**HCN2*	hyperpolarization activated cyclic nucleotide-gated potassium channel 2	2.05	0.014237
A_32_P101689	*FAM3C*	family with sequence similarity 3, member C	2.05	0.031975
A_23_P57521	*EIF3L*	eukaryotic translation initiation factor 3, subunit L	2.07	0.034268
A_24_P391368	*ATXN10*	ataxin 10	2.07	0.023807
A_23_P106844	*MT2A*	metallothionein 2A	2.07	0.026556
A_33_P3344951	*JMJD7*	jumonji domain containing 7	2.08	0.045497
A_33_P3299599	*NKX2-5*	NK2 homeobox 5	2.09	0.029636
A_33_P3336632	*RNA28S5*	RNA, 28S ribosomal 5	2.09	0.023986
A_33_P3330125	*DIABLO*	diablo, IAP-binding mitochondrial protein	2.10	0.017621
A_33_P3329419	*DNM1*	dynamin 1	2.10	0.007567
A_33_P3342628	*HES4*	hes family bHLH transcription factor 4	2.11	0.041278
A_23_P47208	*BANF1*	barrier to autointegration factor 1	2.12	0.029568
A_23_P200598	*MUL1*	mitochondrial E3 ubiquitin protein ligase 1	2.12	0.031255

A_33_P3223713	*UBE2E3*	ubiquitin-conjugating enzyme E2E 3	2.13	0.047590
A_23_P75453	*MEN1*	multiple endocrine neoplasia I	2.14	0.029203
A_23_P74843	*IARS2*	isoleucyl-tRNA synthetase 2, mitochondrial	2.15	0.009192
A_24_P16124	*IFITM4P*	interferon induced transmembrane protein 4 pseudogene	2.15	0.026436
A_33_P3373375	*CD81*	CD81 molecule	2.15	0.048712
A_19_P00319282	**CAV2*	caveolin 2	2.16	0.031280
A_24_P299318	*FAM101B*	family with sequence similarity 101, member B	2.17	0.024554
A_19_P00322249	**BCYRN1*	brain cytoplasmic RNA 1	2.17	0.026269
A_33_P3319760	*TAGLN2*	transgelin 2	2.18	0.035028
A_23_P252322	*ATP5E*	ATP synthase, H+ transporting, mitochondrial F1 complex, epsilon subunit	2.18	0.042453
A_32_P9382	*MZT1*	mitotic spindle organizing protein 1	2.18	0.026282
A_23_P255104	*LHFPL2*	lipoma HMGIC fusion partner-like 2	2.19	0.046781
A_23_P87257	*MRPL17*	mitochondrial ribosomal protein L17	2.20	0.038629
A_23_P13183	*EXT2*	exostosin glycosyltransferase 2	2.20	0.034539
A_23_P15305	*PRPSAP1*	phosphoribosyl pyrophosphate synthetase-associated protein 1	2.20	0.028571
A_23_P348298	*SAC3D1*	SAC3 domain containing 1	2.21	0.020527
A_23_P36408	*VPS33A*	vacuolar protein sorting 33 homolog A (S. cerevisiae)	2.21	0.026924
A_23_P94932	*MMADHC*	methylmalonic aciduria (cobalamin deficiency) cblD type, with homocystinuria	2.21	0.018938
A_23_P9293	*TJP2*	tight junction protein 2	2.21	0.007337
A_23_P256172	*NDUFAF3*	NADH dehydrogenase (ubiquinone) complex I, assembly factor 3	2.22	0.048901
A_23_P425587	*MED11*	mediator complex subunit 11	2.23	0.027862
A_33_P3380932	*ZNF800*	zinc finger protein 800	2.23	0.024129
A_19_P00319331	*HUWE1*	HECT, UBA and WWE domain containing 1, E3 ubiquitin protein ligase	2.24	0.007731
A_33_P3252333	*SNORD3B-1*	small nucleolar RNA, C/D box 3B-1	2.24	0.013697
A_23_P28466	*DAW1*	dynein assembly factor with WDR repeat domains 1	2.25	0.022689
A_32_P122402	*LOC441455*	makorin ring finger protein 1 pseudogene	2.26	0.046377
A_19_P00800006	*Q59GX9*	Q59GX9_HUMAN (Q59GX9) Ribosomal protein L5 variant (Fragment), partial (51%) [THC2523617]	2.27	0.002716
A_19_P00807336	*PPA1*	pyrophosphatase (inorganic) 1	2.28	0.039495
A_23_P82748	*ENY2*	enhancer of yellow 2 homolog (Drosophila)	2.28	0.011520
A_23_P144816	*VDAC1*	voltage-dependent anion channel 1	2.29	0.014015
A_33_P3371055	*RAB1A*	RAB1A, member RAS oncogene family	2.29	0.040549
A_23_P1056	*GPR89B*	G protein-coupled receptor 89B	2.30	0.048802
A_23_P339480	*HAT1*	histone acetyltransferase 1	2.30	0.001917
A_24_P110284	*FLJ25758*	MAP/microtubule affinity-regulating kinase 1 pseudogene	2.30	0.038405
A_23_P88249	*RBM23*	RNA binding motif protein 23	2.31	0.020061
A_23_P105044	*MRPL23*	mitochondrial ribosomal protein L23	2.32	0.031227
A_33_P3265479	*ZFAT*	zinc finger and AT hook domain containing	2.33	0.011977
A_23_P126752	*CAPZB*	capping protein (actin filament) muscle Z-line, beta	2.34	0.027548
A_33_P3531857	*DARS*	aspartyl-tRNA synthetase	2.34	0.014808
A_33_P3416797	*OVOS2*	ovostatin 2	2.35	0.005614
A_33_P3378772	*FAM98B*	family with sequence similarity 98, member B	2.35	0.002117
A_33_P3250963	**TP53TG1*	TP53 target 1 (non-protein coding)	2.35	0.039735
A_23_P61633	*TNK2*	tyrosine kinase, non-receptor, 2	2.37	0.003331

A_24_P217848	*HIST1H2AK*	histone cluster 1, H2ak	2.37	0.022764
A_23_P3355	*POLG*	polymerase (DNA directed), gamma	2.38	0.001517
A_33_P3360684	*CORO1B*	coronin, actin binding protein, 1B	2.39	0.035358
A_23_P86493	*LBX1*	ladybird homeobox 1	2.41	0.006617
A_23_P145197	*BYSL*	bystin-like	2.42	0.004079
A_24_P772436	*DCTN1-AS1*	DCTN1 antisense RNA 1	2.42	0.023062
A_23_P104892	*EIF4G2*	eukaryotic translation initiation factor 4 gamma, 2	2.42	0.018922
A_33_P3295148	*GLUD1P3*	glutamate dehydrogenase 1 pseudogene 3	2.44	0.037792
A_33_P3299279	*ANXA2R*	annexin A2 receptor	2.46	0.041355
A_23_P94683	*NFX1*	nuclear transcription factor, X-box binding 1	2.50	0.038030
A_23_P3514	*C16orf80*	chromosome 16 open reading frame 80	2.51	0.007529
A_33_P3392842	*LOC730668*	dynein heavy chain -like pseudogene	2.51	0.008708
A_23_P170574	*SNAI3*	snail family zinc finger 3	2.53	0.038385
A_19_P00331623	**XIST*	X inactive specific transcript (non-protein coding)	2.53	0.045035
A_23_P132226	*TPST2*	tyrosylprotein sulfotransferase 2	2.53	0.037364
A_23_P58763	*PELO*	pelota homolog (Drosophila)	2.54	0.000312
A_23_P165840	*ODC1*	ornithine decarboxylase 1	2.54	0.001219
A_24_P330971	*EIF3K*	eukaryotic translation initiation factor 3, subunit K	2.55	0.037454
A_33_P3406861	*SORBS2*	sorbin and SH3 domain containing 2	2.56	0.039506
A_24_P303874	*C9orf62*	chromosome 9 open reading frame 62	2.56	0.043912
A_32_P207180	*ARL16*	ADP-ribosylation factor-like 16	2.56	0.030342
A_33_P3369039	*PRPF31*	pre-mRNA processing factor 31	2.59	0.002005
A_33_P3257523	*ZNF451*	zinc finger protein 451	2.60	0.030210
A_33_P3332150	*APOBEC4*	apolipoprotein B mRNA editing enzyme, catalytic polypeptide-like 4 (putative)	2.62	0.032001
A_33_P3303212	*CCDC74A*	coiled-coil domain containing 74A	2.62	0.013728
A_23_P414519	*NRN1*	neuritin 1	2.66	0.032449
A_32_P76811	*HLA-F-AS1*	HLA-F antisense RNA 1	2.68	0.032836
A_19_P00812587	**RPL5*	ribosomal protein L5	2.73	0.000253
A_33_P3405995	*YBEY*	ybeY metallopeptidase (putative)	2.73	0.033320
A_23_P307430	*LSM12*	LSM12 homolog (S. cerevisiae)	2.74	0.042377
A_24_P109554	*CNTRL*	centriolin	2.74	0.005104
A_23_P43141	*EIF3E*	eukaryotic translation initiation factor 3, subunit E	2.75	0.006347
A_23_P96325	*ERCC6L*	excision repair cross-complementation group 6-like	2.77	0.028465
A_33_P3311210	*BSX*	brain-specific homeobox	2.79	0.049200
A_33_P3291816	*CLDN22*	claudin 22	2.83	0.005764
A_23_P134517	*PURB*	purine-rich element binding protein B	2.83	0.042457
A_33_P3372688	*BHLHA9*	basic helix-loop-helix family, member a9	2.83	0.005786
A_23_P20777	*RBM18*	RNA binding motif protein 18	2.83	0.020464
A_23_P123256	*PDAP1*	PDGFA associated protein 1	2.86	0.048787
A_23_P138514	*COMMD3*	COMM domain containing 3	2.87	0.034481
A_23_P20248	*MAP2K1*	mitogen-activated protein kinase kinase 1	2.90	0.047555
A_33_P3240972	*LOC102723925*	golgin subfamily A member 6-like protein 2-like	2.93	0.033642
A_23_P250607	*PLS3*	plastin 3	2.93	0.016905
A_24_P405621	*NISCH*	nischarin	3.05	0.035284

A_33_P3313595	*CDK11B*	cyclin-dependent kinase 11B	3.07	0.010304
A_23_P381261	*ADCY4*	adenylate cyclase 4	3.11	0.000567
A_23_P217054	*DCAF10*	DDB1 and CUL4 associated factor 10	3.13	0.044545
A_19_P00803960	*THAP9-AS1*	THAP9 antisense RNA 1	3.14	0.006249
A_23_P16573	*DDX49*	DEAD (Asp-Glu-Ala-Asp) box polypeptide 49	3.19	0.041513
A_23_P316239	*CXorf40B*	chromosome X open reading frame 40B	3.26	0.019863
A_23_P149664	*TMEM183B*	transmembrane protein 183B	3.29	0.000576
A_33_P3368203	*ASIC2*	acid-sensing (proton-gated) ion channel 2	3.31	0.005025
A_23_P168368	*C7orf69*	chromosome 7 open reading frame 69	3.31	0.015381
A_24_P271049	*C18orf32*	chromosome 18 open reading frame 32	3.36	0.019415
A_33_P3332492	*FANK1*	fibronectin type III and ankyrin repeat domains 1	3.38	0.041060
A_23_P132341	*C22orf46*	chromosome 22 open reading frame 46	3.38	0.047627
A_33_P3306894	*KIAA1549L*	KIAA1549-like	3.49	0.001798
A_24_P340679	**PPIA*	peptidylprolyl isomerase A (cyclophilin A)	3.50	0.032789
A_23_P141847	*UBE2M*	ubiquitin-conjugating enzyme E2M	3.53	0.018313
A_24_P41570	*H2AFZ*	H2A histone family, member Z	3.53	0.046962
A_23_P110571	*MAST4*	microtubule associated serine/threonine kinase family member 4	3.55	0.010230
A_23_P164100	*C17orf64*	chromosome 17 open reading frame 64	3.69	0.031713
A_33_P3359219	*PPAPDC1B*	phosphatidic acid phosphatase type 2 domain containing 1B	3.77	0.000450
A_32_P23010	*SDHAF1*	succinate dehydrogenase complex assembly factor 1	3.77	0.026671
A_24_P289648	*HFE*	hemochromatosis	3.95	0.000556
A_23_P97309	*CASP9*	caspase 9, apoptosis-related cysteine peptidase	4.20	0.018859
A_32_P3556	*VPS35*	vacuolar protein sorting 35 homolog (S. cerevisiae)	4.32	0.032045
A_33_P3208975	*OR51S1*	olfactory receptor, family 51, subfamily S, member 1	4.93	0.000122
A_33_P3322499	*ZNF833P*	zinc finger protein 833, pseudogene	5.24	0.028962
A_23_P150609	*IGF2*	insulin-like growth factor 2 (somatomedin A)	7.22	0.013084

Microarray analyses of hiPSC-derived motor neurons from sporadic ALS and non-ALS subjects. Motor neurons were differentiated of hiPSC reprogrammed from fibroblasts obtained from ALS and non-ALS subjects as described in the text. Positive and negative values correspond to up and down regulation changes, respectively. * two probes for the same gene.

Table S2. Molecular Function GO terms from differentially expressed gene list from hiPSC-derived motor neurons from sporadic ALS patients compared to non-ALS subjects obtained from microarray analysis and genes included in these GO terms.

Term number	GO number	GO Term	Number of genes
1	GO:0000166	nucleotide binding	215
2	GO:0003677	DNA binding	209
3	GO:0017076	purine nucleotide binding	184
4	GO:0032555	purine ribonucleotide binding	181
5	GO:0032553	ribonucleotide binding	181
6	GO:0001883	purine nucleoside binding	153
7	GO:0001882	nucleoside binding	153
8	GO:0030554	adenyl nucleotide binding	152
9	GO:0032559	adenyl ribonucleotide binding	149
10	GO:0005524	ATP binding	148
11	GO:0003723	RNA binding	79
12	GO:0042802	identical protein binding	63
13	GO:0008092	cytoskeletal protein binding	53
14	GO:0016887	ATPase activity	50
15	GO:0042623	ATPase activity, coupled	42
16	GO:0019001	guanyl nucleotide binding	41
17	GO:0032561	guanyl ribonucleotide binding	41
18	GO:0005525	GTP binding	40
19	GO:0016564	transcription repressor activity	34
20	GO:0004386	helicase activity	26
21	GO:0070035	purine NTP-dependent helicase activity	20
22	GO:0008026	ATP-dependent helicase activity	20
23	GO:0003714	transcription corepressor activity	19
24	GO:0051082	unfolded protein binding	18
25	GO:0008022	protein C-terminus binding	18
26	GO:0008135	translation factor activity, nucleic acid binding	14
27	GO:0008094	DNA-dependent ATPase activity	13
28	GO:0003729	mRNA binding	12
29	GO:0016811	hydrolase activity, acting on carbon-nitrogen (but not peptide) bonds, in linear amides	11
30	GO:0016655	oxidoreductase activity, acting on NADH or NADPH, quinone or similar compound as acceptor	10
31	GO:0003678	DNA helicase activity	9
32	GO:0050136	NADH dehydrogenase (quinone) activity	8
33	GO:0003954	NADH dehydrogenase activity	8
34	GO:0008137	NADH dehydrogenase (ubiquinone) activity	8
35	GO:0051087	chaperone binding	7
36	GO:0003724	RNA helicase activity	7
37	GO:0003725	double-stranded RNA binding	7
38	GO:0003746	translation elongation factor activity	6
39	GO:0004003	ATP-dependent DNA helicase activity	6
40	GO:0005520	insulin-like growth factor binding	6
41	GO:0005487	nucleocytoplasmic transporter activity	4
42	GO:0050750	low-density lipoprotein receptor binding	4

Genes in Molecular Function GO terms			
ABCA6 (1,3,4,5,6,7,8,9,10,14)	*ERBB2* (1,3,4,5,6,7,8,9,10,12,25)	*MUL1* (12)	*RRAS2* (1,3,4,5,16,17,18)
ABCA9 (1,3,4,5,6,7,8,9,10,14)	*ERCC3* (1,2,3,4,5,6,7,8,9,10,14,15,16,17,18,20,21,22,25,28,31,39)	*MXD4* (2,19,23)	*RREB1* (2)
ABCC5 (1,3,4,5,6,7,8,9,10,14,15)	*ERCC6L* (1,2,3,4,5,6,7,8,9,10,20)	*MYBBP1A* (2)	*RUNX3* (1,2,3,4,5,6,7,8,9,10)
ABCG4 (1,3,4,5,6,7,8,9,10,12,14)	*ERCC8* (14,15,20,27,31)	*MYH11* (1,3,4,5,6,7,8,9,10,13)	*RUVBL2* (1,2,3,4,5,6,7,8,9,10,12,14,15,20,21,22,24,27,31,39)
ABLIM1 (13)	*ESRP2* (1,11,28)	*MYO19* (1,3,4,5,6,7,8,9,10,13)	*RYR2* (12)
ABLIM3 (13)	*ESRRB* (2)	*MYO1B* (1,3,4,5,6,7,8,9,10,13)	*S100A11* (12)
ABTB1 (26,38)	*ETV7* (2)	*MYO3A* (1,3,4,5,6,7,8,9,10,13,14,15)	*SALL2* (2)
ACACB (1,3,4,5,6,7,8,9,10)	*EVL* (13)	*MYO6* (1,3,4,5,6,7,8,9,10,13)	*SATB1* (2)
ACAD9 (1,3,6,7,8)	*EXT2* (12)	*NAB2* (19,23)	*SCYL1* (1,2,3,4,5,6,7,8,9,10)
ACD (2)	*FANCG* (2)	*NAV2* (1,3,4,5,6,7,8,9,10,20)	*SECISBP2* (11,28)
ACSBG1 (1,3,4,5,6,7,8,9,10)	*FARP2* (13)	*NCBP1* (11)	*SERPINH1* (24)
ACSM2B (1,3,4,5,6,7,8,9,10)	*FASTKD1* (1,3,4,5,6,7,8,9,10)	*NCF1* (1,3,4,5,10,17,18)	*SGK223* (1,3,4,5,6,7,8,9,10)
ACTG2 (1,3,4,5,6,7,8,9,10)	*FHL2* (12)	*NCK1* (13)	*SHARPIN* (12)
ADA (6,7)	*FIGNL2* (1,3,4,5,6,7,8,9,10)	*NCK2* (13)	*SIRT3* (1,29)
ADCY4 (1,3,4,5,6,7,8,9,10)	*FKBP4* (1,3,4,5,6,7,8,9,10,16,17,18)	*NCOR1* (2,19,23)	*SIRT5* (1,29)
ADD2 (12,13)	*FLT4* (1,3,4,5,6,7,8,9,10)	*ND2* (30,32,33,34)	*SIX1* (2)
ADRBK1 (1,3,4,5,6,7,8,9,10)	*FNTA* (13)	*NDUFA12* (30,32,33,34)	*SLC9A3R1* (25)
ADRBK2 (1,3,4,5,6,7,8,9,10)	*FOXE1* (2,19)	*NDUFA2* (30,32,33,34)	*SMAD4* (2,12)
AGAP3 (1,3,4,5,16,17,18)	*FOXJ2* (2)	*NDUFA4* (30,32,33,34)	*SMAD7* (2)
AK3 (1,3,4,5,6,7,8,9,10,12,16,7,18)	*FOXN2* (2)	*NDUFB5* (30,32,33,34)	*SMARCA5* (1,2,3,4,5,6,7,9,10,14,20)
ALKBH3 (2)	*FXR1* (11)	*NDUFB6* (30,32,33,34)	*SMARCAL1* (1,2,3,4,5,6,7,8,9,10,14,15,20,27,31)
AMOTL2 (12)	*GABARAP* (13)	*NDUFC2* (30,32,33,34)	*SMC2* (1,3,4,5,6,7,8,9,10)
ANXA2 (13)	*GABPB1* (2)	*NDUFV3* (30,32,33,34)	*SMC4* (1,3,4,5,6,7,8,9,10)
APOB (42)	*GALC* (29)	*NFAT5* (2)	*SMG6* (2)
APOE (12,13,42)	*GBP7* (1,3,4,5,16,17,18)	*NFE2L1* (2)	*SMN1* (11)
APTX (2,11,37)	*GFM1* (1,3,4,5,16,17,18,26,38)	*NFIB* (2)	*SNAI3* (2)

ARFRP1 [1,3,4,5,16,17,18]
ARID5A [2,19]
ARL16 [1,3,4,5,16,17,18]
ARL8A [1,3,4,5,13,16,17,18]
ARL9 [1,3,4,5,16,17,18]
ARPC1A [13]
ASAH1 [29]
ASAH2 [29]
ASCL3 [2]
ASH1L [2]
ATAD3A [1,3,4,5,6,7,8,9,10]
ATAD3B [1,3,4,5,6,7,8,9,10]
ATF3 [2,12,19,23]
ATF4 [2,25]
ATF7IP [14,19,23]
ATL2 [1,3,4,5,12,16,17,18]
ATM [1,2,3,4,5,6,7,8,9,10]
ATP1A4 [1,3,4,5,6,7,8,9,10,14,15]
ATP1B1 [14,15]
ATP4B [14,15]
ATP5B [1,3,4,5,6,7,8,9,10,14,15]
ATP5C1 [14,15]
ATP5D [1,3,4,5,6,7,8,9,10,14,15]
ATP5E [14,15]
ATP6V1D [14,15]
ATP8B3 [1,3,4,5,6,7,8,9,10,14,15]
ATP9A [1,3,4,5,6,7,8,9,10,14,15]
AURKB [1,3,4,5,6,7,8,9,10]
BAIAP2L1 [13]
BANF1 [2]
BARHL2 [2]
BATF [2]
BBX [2]
BCL10 [25]
BCR [1,3,4,5,6,7,8,9,10]
BHLHA9 [2]
BLOC1S2 [12,13]
BLOC1S2 [25]
BMPR1A [1,3,4,5,6,7,8,9,10,12]
BSX [2]
CADM1 [12,25]
CALM2 [13]
CAPN1 [13]
CAPZB [13]
CAV2 [12]
CBFA2T2 [2,19,23]
CBR3 [1]
CBWD5 [1,3,4,5,6,7,8,9,10]
CBX5 [19]
CCT3 [1,3,4,5,6,7,8,9,10,24]
CDK10 [1,3,4,5,6,7,8,9,10]
CDK11B [1,3,4,5,6,7,8,9,10]
CDK19 [1,3,4,5,6,7,8,9,10]
CDT1 [2]
CFL1 [13]
CHD3 [1,2,3,4,5,6,7,8,9,10,14,15,20,21,22,27,31,39]
CHD4 [1,2,3,4,5,6,7,8,9,10,14,15,20,21,22,27,31,39]
CHD5 [1,2,3,4,5,6,7,8,9,10,14,15,20,21,22]
CHUK [1,2,3,4,5,6,7,8,9,10,12]
CKMT1A [1,3,4,5,6,7,8,9,10]
CLDN12 [12]
CLDN22 [12]
COIL [25]
CORO1B [13]
CPOX [12]
CPSF1 [11,28]
CROCC [13]
CSNK2B [12]
CSTF2 [1,11]
CSTF3 [11]
CTPS2 [1,3,4,5,6,7,8,9,10]
CXXC5 [2]
DAK [1,3,4,5,6,7,8,9,10]
DARS [1,3,4,5,6,7,8,9,10,29]
DBN1 [13]
GFM2 [1,3,4,5,16,17,18,26,38]
GIMAP6 [1,3,4,5,16,17,18]
GLI4 [2]
GLS [29]
GNE [1,3,4,5,,6,7,8,9,10]
GNL3 [1,3,4,5,16,17,18]
GPN2 [1,3,4,5,16,17,18]
GRPEL1 [1,3,6,7,8,12,24,35]
GSS [1,3,4,5,6,7,8,9,10,12]
GSTO1 [30]
GTF2F2 [1,2,3,4,5,6,7,8,9,10,14,15,20,21,22]
GTF2H5 [2]
GTF2IRD1 [2]
GUCY2F [1,3,4,5,6,7,8,9,10,16,17,18]
GUF1 [1,3,4,5,16,17,18]
H1FX [2]
H2AFZ [2]
HACL1 [12]
HCN2 [1,3,4,5,6,7,8,9]
HDAC3 [2,19,21,29]
HELT [2,12,19]
HES4 [2]
HES5 [2]
HIP1R [13]
HIST1H2AD [2]
HIST1H2AI [2]
HIST1H2AK [2]
HIST1H2BA [2]
HIST1H2BF [2]
HIST1H2BI [2]
HIST1H4D [2]
HIST1H4K [20]
HIST3H3 [2]
HMG20B [2]
HMGB3 [2]
HMGCR [1,12]
HMGXB3 [2]
HNRNPA2B1 [1,2,11]
HNRNPAB [1,2,11,28]
HNRNPC [1,11,12,28]
HNRNPK [2,11]
HNRNPL [1,11]
HOXA9 [2]
HSBP1 [19,23]
HSP90AA1 [1,3,4,5,6,7,8,9,10,12,24]
HSP90AB1 [1,3,4,5,6,7,9,10,24]
HSPA5 [1,3,4,5,6,7,8,9,10,24]
HSPA8 [1,3,4,5,6,7,8,9,10,14,15,24]
HSPD1 [1,2,3,4,5,6,7,8,9,10,14,24,35]
HTRA1 [40]
HTRA2 [24]
HUWE1 [2]
IARS2 [1,3,4,5,6,7,8,9,10]
ICK [1,3,4,5,6,7,8,9,10]
ID3 [19,23]
IDH2 [1]
IGF2 [40]
IGF2BP3 [1,11,28]
IGFBP2 [40]
IGFBP6 [40]
INSRR [1,3,4,5,6,7,8,9,10]
IP6K2 [1,3,4,5,6,7,8,9,10]
IP6K3 [1,3,4,5,6,7,8,9,10]
IQGAP2 [13]
IRF7 [2]
ITM2B [1,3,4,5,6,7,8,9,10]
JARID2 [2,19]
JDP2 [2,19]
JRK [2,11,28]
KCTD13 [12]
KDM4A [19]
KHSRP [2,11]
KIAA0232 [1,3,4,5,6,7,8,9,10]
KIF1A [1,3,4,5,6,7,9,10]
KIF24 [1,3,4,5,6,7,9,10]
NFX1 [2]
NFYA [2]
NFYC [2]
NHP2 [11]
NIPBL [25]
NKRF [2,11,19,37]
NKX2 [5 (2,12,19]
NKX3 [1 (2]
NLRP11 [1,3,4,5,6,7,8,9,10]
NLRX1 [1,3,4,5,6,7,8,9,10]
NOL8 [1,11]
NOP14 [11]
NOSTRIN [2]
NOSTRIN [19]
NOV [40]
NR5A2 [2]
NR6A1 [2,12]
NRBP2 [1]
NUBP1 [1,3,4,5,6,7,8,9,10]
NUCB1 [2]
NUP107 [41]
NUP133 [41]
NUP160 [41]
NUP54 [41]
NUSAP1 [2,13]
NVL [1,3,4,5,6,7,9,10]
OLA1 [1,3,4,5,6,7,8,9,10,16,17,18]
OOEP [110]
OPA1 [1,3,4,5,16,17,18]
P2RY1 [1,3,4,5,6,7,9,10]
PABPC5 [1,11]
PAFAH1B1 [12,13]
PAIP1 [11]
PCSK1 [35]
PDCD6 [2]
PDZD11 [25]
PEX14 [19,23]
PEX6 [1,3,4,5,6,7,8,9,10,14,15,25]
PFKM [1,3,4,5,6,7,8,9,10,12,25]
PHACTR3 [13]
PHC1 [2]
PI4KB [1,3,4,5,6,7,8,9,10]
PIAS4 [2,19,23]
PIF1 [1,2,3,4,5,6,7,8,9,10,14,15,20,21,22,27,31,39]
PIGL [29]
PIM2 [1,3,4,5,6,7,8,9,10]
PIP4K2B [1,3,4,5,6,7,8,9,10]
PLAG1 [2]
PLS1 [13]
PLS3 [13]
POF1B [13]
POGZ [2]
POLA2 [2]
POLG [2]
POLR1C [2]
POLR2J2 [2]
POU1F1 [2,19,23]
POU3F2 [2]
POU3F2 [12]
PPIA [24]
PPIC [24]
PPIE [1,11]
PPIP5K1 [1,3,4,5,6,7,9,10]
PRKAA1 [1,3,4,5,6,7,8,9,10]
PRKRIP1 [11,37]
PRPF31 [11]
PRPS1 [1,3,4,5,6,7,8,9,10,12,16,17]
PRPSAP1 [12]
PRR12 [2]
PSMA7 [12]
PSMC2 [1,3,4,5,6,7,8,9,10,14]
PSMC4 [1,3,4,5,6,7,8,9,10,14]
PTGES3 [24]
PURB [2,11,19,28]
PXN [13]
SNAPC2 [2]
SNRPE [11]
SNX6 [12]
SOD1 [12,35]
SON [2,11,37]
SORBS2 [13]
SOX15 [2]
SP140 [2]
SPAG9 [13]
SPHK2 [1,3,4,5,6,7,8,9,10]
SPIRE2 [13]
SPR [1]
SPTBN2 [13]
SREBF1 [2]
SRP14 [11]
SRPRB [1,3,4,5,16,17,18]
STAT2 [2]
STAU1 [11,37]
STMN1 [13]
STX4 [13]
SYNJ1 [11]
SYT1 [12,42]
TADA3 [2]
TAF1B [2]
TAF1C [2]
TAF8 [2]
TAGLN [13]
TARDBP [1,2,11,13]
TBCA [24,35]
TBX21 [2]
TCF3 [2,12]
TCP1 [1,3,4,5,6,7,9,10,24]
TDRD9 [1,3,4,5,6,7,8,9,10,14,15,20,21,22]
TERF1 [2,12,13]
TESK1 [1,3,4,5,6,7,8,9,10]
TET1 [2]
TGIF1 [2,19,23]
THOC3 [11]
THRAP3 [1,3,4,5,6,7,9,10]
TIMELESS [12]
TJP2 [25]
TLK2 [1,3,4,5,6,7,8,9,10]
TLR3 [11,37]
TLR9 [11]
TMSB4X [13]
TNK2 [1,3,4,5,6,7,8,9,10]
TOP1 [1,2,3,4,5,6,7,8,9,10]
TOR2A [1,3,4,5,6,7,8,9,10]
TP53I3 [1,12,30]
TPM2 [13]
TRNAU1AP [1,11]
TRUB1 [11]
TSNAX [2]
TTC14 [11]
TUBA1C [1,3,4,5,16,17,18]
TUBB2A [1,3,4,5,16,17,18]
TUBB6 [1,3,4,5,16,17,18]
UBE2E3 [1,3,4,5,6,7,9,10]
UBE2G2 [1,3,4,5,6,7,9,10]
UBE2I [1,3,4,5,6,7,8,9,10,19]
UBE2M [1,3,4,5,6,7,9,10]
UBE2S [1,3,4,5,6,7,9,10]
UBN1 [2]
UCK2 [1,3,4,5,6,7,9,10]
UPF1 [1,2,3,4,5,6,7,8,9,10,11,14,15,20,21,36]
URGCP [1,3,4,5,16,17,18]
USF2 [2,12]
VAT1 [2]
VDR [2]
VNN3 [29]
VPS18 [13]
VWF [12,35]
WISP1 [40]
WNK2 [1,3,4,5,6,7,8,9,10]
XRCC3 [1,2,3,4,5,6,7,8,9,10,14,15,27]

DDX10 (1,3,4,5,6,7,8,9,10,11,4,15,20,21,22,36)	*KIF2C* (1,2,3,4,5,6,7,8,9,10)	*QPRT* (12)	*YWHAB* (19,23,25)
DDX21 (1,3,4,5,6,7,8,9,10,11,14,15,20,21,22,36)	*KIFC1* (1,3,4,5,6,7,8,9,10)	*RAB17* (1,3,4,5,16,17,18)	*YY1AP1* (2)
DDX23 (1,3,4,5,6,7,8,9,10,14,15,20,21,22,36)	*KIFC2* (1,3,4,5,6,7,9,10)	*RAB1A* (1,3,4,5,6,7,8,9,10,16,17,18)	*ZAK* (1,3,4,5,6,7,8,9,10,12)
DDX27 (1,3,4,5,6,7,8,9,10,15,20,21,22)	*KLHL17* (13)	*RAB1B* (1,3,4,5,16,17,18)	*ZBTB20* (2)
DDX49 (1,3,4,5,6,7,8,9,10,11,14,15,20,21,22)	*KSR2* (1,3,4,5,6,7,8,9,10)	*RAB37* (1,3,4,5,16,17,18)	*ZBTB43* (2)
DDX54 (1,3,4,5,6,7,8,9,10,11,14,15,19,20,21,22,23,36)	*LBR* (2)	*RAB3A* (1,3,4,5,16,17,18)	*ZBTB45* (2)
DDX6 (1,3,4,5,6,7,8,9,10,11,14,15,20,21,22,36)	*LBX1* (2)	*RAB8A* (1,3,4,5,16,17,18)	*ZC3HAV1* (11)
DFFA (12)	*LDLRAP1* (25)	*RABGEF1* (2)	*ZFAND5* (2)
DHFR (1)	*LHX2* (2)	*RAD54L* (1,2,3,4,5,6,7,8,9,10,20)	*ZFAT* (2)
DHX58 (1,3,4,5,6,7,8,9,10,11,14,15,20,21,22)	*LIMA1* (13)	*RALB* (1,3,4,5,16,17,18)	*ZFP36L1* (2,11,18)
DHX9 (1,2,3,4,5,6,7,8,9,10,11,14,15,20,21,22,27,31,36,37,39)	*LMO4* (2)	*RAP1B* (1,3,4,5,16,17,18)	*ZFP36L2* (2,11)
DIRAS2 (1,3,4,5,16,17,18)	*LONRF3* (14,15)	*RASSF1* (12)	*ZFP41* (2)
DIS3L2 (11)	*LRRK2* (1,3,4,5,6,7,8,9,10,12,16,17,18)	*RBBP4* (14,15,27)	*ZHX3* (2)
DLG1 (13,25)	*LTK* (1,3,4,5,6,7,8,9,10)	*RBM10* (1,11)	*ZMYM6* (2)
DMPK (1,3,4,5,6,7,8,9,10)	*MAF* (2)	*RBM14* (1,11)	*ZNF169* (2)
DMRTA2 (2)	*MAFB* (2)	*RBM18* (1,11)	*ZNF20* (2)
DNAJA1 (24,42)	*MAP1B* (13)	*RBM20* (11)	*ZNF207* (2)
DNAJA3 (24)	*MAP2K1* (1,3,4,5,6,7,8,9,10)	*RBM23* (1,11)	*ZNF229* (2)
DNAJB1 (24)	*MAP3K5* (1,3,4,5,6,7,8,9,10,12)	*RBM8A* (1,11,28)	*ZNF233* (2)
DNAJB6 (2,19,35)	*MAPK15* (1,3,4,5,6,7,8,9,10)	*RBMS1* (1,2,11)	*ZNF248* (2)
DNM1 (1,3,4,5,16,17,18)	*MARK2* (1,3,4,5,6,7,8,9,10)	*RECQL5* (1,3,4,5,6,7,8,9,10,14,15,20,21,22,31)	*ZNF260* (2)
DNMT1 (2)	*MARS* (1,3,4,5,6,7,9,10,11)	*RENBP* (1,3,4,5,6,7,8,9,10,12)	*ZNF268* (2)
DRAP1 (2,19,23)	*MAST4* (1,3,4,5,6,7,8,9,10)	*RFC2* (1,2,3,4,5,6,7,8,9,10,14,15,27)	*ZNF3* (2,12)
DTD1 (1,3,4,5,6,7,8,9,10)	*MBNL2* (11)	*RFC4* (1,2,3,4,5,6,7,8,9,10,14,15,27)	*ZNF302* (2)
DYNLL1 (25)	*MCM3AP* (2)	*RFC5* (1,2,3,4,5,6,7,8,9,10,14,15,27)	*ZNF324B* (2)
E2F1 (2,19,23)	*MEF2C* (2)	*RFX1* (2)	*ZNF326* (2)
E2F3 (2)	*MEF2D* (1,2)	*RFX7* (2)	*ZNF329* (2)
EED (12,19)	*MEF2D* (2)	*RILPL2* (12)	*ZNF41* (2)
EEF1A1 (1,3,4,5,16,17,18,26,38)	*MEN1* (2)	*RIOK3* (1,3,4,5,6,7,8,9,10)	*ZNF451* (2)
EEF1B2 (26,38)	*MESP1* (2)	*RNASET2* (11)	*ZNF462* (2)
EFTUD1 (1,3,4,5,16,17,18,26,38)	*MEX3D* (11)	*ROBO2* (12)	*ZNF467* (2)
EHD4 (1,3,4,5,6,7,8,9,10,16,17,18)	*MGA* (2)	*ROR1* (1,3,4,5,6,7,8,9,10)	*ZNF497* (2)
EIF2B3 (26)	*MIER2* (2)	*RPA3* (2)	*ZNF512* (2)
EIF3E (26)	*MIF4GD* (25)	*RPF1* (11)	*ZNF561* (2)
EIF3G (1,11,26)	*MKNK2* (1,3,4,5,6,7,9,10)	*RPL19P12* (11)	*ZNF658* (2)
EIF3K (26)	*MLLT10* (2)	*RPL21* (11)	*ZNF662* (2)
EIF3L (26)	*MLPH* (13)	*RPL23A* (1,11)	*ZNF681* (2)
EIF4A2 (1,3,4,5,6,7,8,9,10,11,14,15,20,21,22,26)	*MNT* (2,19,23)	*RPL24* (11)	*ZNF69* (2)
EIF4G2 (26)	*MNX1* (2)	*RPL39* (11)	*ZNF71* (2)
ELP2 (2)	*MRPL23* (1,11)	*RPL5* (11)	*ZNF738* (2)
ENO1 (2,19,23)	*MRPS6* (11)	*RPL7* (2,11,12,28)	*ZNF765* (2)
ENO3 (12)	*MTA2* (2,19,29)	*RPS14* (11,28)	*ZNF775* (2)
EPHA4 (1,3,4,5,6,7,8,9,10)	*MTIF3* (26)	*RPS15A* (11)	*ZNF800* (2)
EPRS (1,3,4,5,6,7,8,9,10,11)	*MTRR* (1,3,6,7,8)	*RPS2* (11)	*ZZZ3* (2)

Microarray analyses of hiPSC-derived motor neurons from sporadic ALS and non-ALS subjects. Motor neurons were differentiated of hiPSC reprogrammed from fibroblasts obtained from ALS and non-ALS subjects as described in the text. The numbers between parentheses indicate Molecular Function GO term(s) in which the genes are included.

Table S3. Biological Process GO terms from differentially expressed gene list from hiPSC-derived motor neurons from sporadic ALS patients compared to non-ALS subjects obtained from microarray analysis and genes included in these GO terms.

Term number	GO number	GO Term	Number of genes
1	GO:0010604	positive regulation of macromolecule metabolic process	86
2	GO:0043933	macromolecular complex subunit organization	84
3	GO:0010605	negative regulation of macromolecule metabolic process	81
4	GO:0006357	regulation of transcription from RNA polymerase II promoter	77
5	GO:0009057	macromolecule catabolic process	76
6	GO:0044265	cellular macromolecule catabolic process	75
7	GO:0065003	macromolecular complex assembly	77
8	GO:0046907	intracellular transport	73
9	GO:0008219	cell death	71
10	GO:0016265	death	71
11	GO:0009890	negative regulation of biosynthetic process	69
12	GO:0009891	positive regulation of biosynthetic process	67
13	GO:0031327	negative regulation of cellular biosynthetic process	66
14	GO:0010558	negative regulation of macromolecule biosynthetic process	65
15	GO:0051276	chromosome organization	67
16	GO:0051173	positive regulation of nitrogen compound metabolic process	64
17	GO:0045935	positive regulation of nucleobase, nucleoside, nucleotide and nucleic acid metabolic process	61
18	GO:0045934	negative regulation of nucleobase, nucleoside, nucleotide and nucleic acid metabolic process	59
19	GO:0051172	negative regulation of nitrogen compound metabolic process	59
20	GO:0010629	negative regulation of gene expression	57
21	GO:0043632	modification-dependent macromolecule catabolic process	57
22	GO:0019941	modification-dependent protein catabolic process	57
23	GO:0032268	regulation of cellular protein metabolic process	56
24	GO:0006259	DNA metabolic process	56
25	GO:0016481	negative regulation of transcription	54
26	GO:0051254	positive regulation of RNA metabolic process	53
27	GO:0006325	chromatin organization	54
28	GO:0045893	positive regulation of transcription, DNA-dependent	51
29	GO:0034621	cellular macromolecular complex subunit organization	53
30	GO:0006412	translation	46
31	GO:0008283	cell proliferation	46
32	GO:0045892	negative regulation of transcription, DNA-dependent	44
33	GO:0051253	negative regulation of RNA metabolic process	44
34	GO:0070727	cellular macromolecule localization	46
35	GO:0034622	cellular macromolecular complex assembly	43
36	GO:0034613	cellular protein localization	43
37	GO:0022403	cell cycle phase	41
38	GO:0016071	mRNA metabolic process	40
39	GO:0000278	mitotic cell cycle	40
40	GO:0006886	intracellular protein transport	39
41	GO:0000279	M phase	36
42	GO:0016568	chromatin modification	36
43	GO:0008380	RNA splicing	33
44	GO:0010035	response to inorganic substance	31
45	GO:0010608	posttranscriptional regulation of gene expression	31
46	GO:0048285	organelle fission	30
47	GO:0000122	negative regulation of transcription from RNA polymerase II promoter	30
48	GO:0000087	M phase of mitotic cell cycle	29
49	GO:0006457	protein folding	28
50	GO:0007067	mitosis	28
51	GO:0000280	nuclear division	28
52	GO:0010627	regulation of protein kinase cascade	28
53	GO:0060284	regulation of cell development	27
54	GO:0032270	positive regulation of cellular protein metabolic process	27
55	GO:0006605	protein targeting	26
56	GO:0033043	regulation of organelle organization	26
57	GO:0001558	regulation of cell growth	25
58	GO:0022613	ribonucleoprotein complex biogenesis	24
59	GO:0006260	DNA replication	24
60	GO:0050767	regulation of neurogenesis	23
61	GO:0031401	positive regulation of protein modification process	23
62	GO:0051960	regulation of nervous system development	23
63	GO:0006417	regulation of translation	22
64	GO:0032269	negative regulation of cellular protein metabolic process	22
65	GO:0007005	mitochondrion organization	21
66	GO:0000377	RNA splicing, via transesterification reactions with bulged adenosine as nucleophile	21
67	GO:0000398	nuclear mRNA splicing, via spliceosome	21
68	GO:0000375	RNA splicing, via transesterification reactions	21
69	GO:0043623	cellular protein complex assembly	21

70	GO:0045664	regulation of neuron differentiation	19
71	GO:0043122	regulation of I-kappaB kinase/NF-kappaB cascade	18
72	GO:0032583	regulation of gene-specific transcription	18
73	GO:0043405	regulation of MAP kinase activity	18
74	GO:0006414	translational elongation	17
75	GO:0006333	chromatin assembly or disassembly	20
76	GO:0006323	DNA packaging	19
77	GO:0043123	positive regulation of I-kappaB kinase/NF-kappaB cascade	15
78	GO:0006403	RNA localization	15
79	GO:0007568	aging	15
80	GO:0000302	response to reactive oxygen species	14
81	GO:0031398	positive regulation of protein ubiquitination	14
82	GO:0051236	establishment of RNA localization	14
83	GO:0050657	nucleic acid transport	14
84	GO:0050658	RNA transport	14
85	GO:0031396	regulation of protein ubiquitination	14
86	GO:0016125	sterol metabolic process	14
87	GO:0052548	regulation of endopeptidase activity	13
88	GO:0052547	regulation of peptidase activity	13
89	GO:0051028	mRNA transport	13
90	GO:0043254	regulation of protein complex assembly	13
91	GO:0022618	ribonucleoprotein complex assembly	12
92	GO:0010769	regulation of cell morphogenesis involved in differentiation	12
93	GO:0043281	regulation of caspase activity	12
94	GO:0010975	regulation of neuron projection development	11
95	GO:0050770	regulation of axonogenesis	10
96	GO:0050808	synapse organization	10
97	GO:0008064	regulation of actin polymerization or depolymerization	10
98	GO:0007163	establishment or maintenance of cell polarity	9
99	GO:0043244	regulation of protein complex disassembly	9
100	GO:0043392	negative regulation of DNA binding	9
101	GO:0000956	nuclear-transcribed mRNA catabolic process	8
102	GO:0034599	cellular response to oxidative stress	8
103	GO:0006402	mRNA catabolic process	8
104	GO:0050810	regulation of steroid biosynthetic process	7
105	GO:0034614	cellular response to reactive oxygen species	7
106	GO:0006406	mRNA export from nucleus	7
107	GO:0045540	regulation of cholesterol biosynthetic process	6
108	GO:0009146	purine nucleoside triphosphate catabolic process	6
109	GO:0006942	regulation of striated muscle contraction	6
110	GO:0060113	inner ear receptor cell differentiation	6
111	GO:0009143	nucleoside triphosphate catabolic process	6
112	GO:0006200	ATP catabolic process	5
113	GO:0042491	auditory receptor cell differentiation	5
114	GO:0055117	regulation of cardiac muscle contraction	4
115	GO:0051972	regulation of telomerase activity	4
116	GO:0070303	negative regulation of stress-activated protein kinase signaling pathway	4
117	GO:0046329	negative regulation of JNK cascade	4
118	GO:0006379	mRNA cleavage	4

Genes in Biological Process GO terms

AAAS [8]
ABCG4 [12,104,107]
ABLIM3 [1,4,12,16,17,26,28]
ABTB1 [30]
ACAA2 [86]
ACD [3,8,11,13,14,15,18,19,24,34,36,40,56,115]
ADA [44,79,80,108,111]
ADAMTSL4 [9,10]
ADD2 [56,90,97,90]
ADRBK1 [44,79,80,109]
AGAP3 [8,34,36,40,79]
AGAP3 [34,36,40,79]
AIDA [52,116,117]
AKAP12 [834,36,40,55]
ALG11 [37,41,58]
ALKBH3 [24]
ALMS1 [9,10,31]
ANAPC1 [1,3,5,6,21,22,23,37,39,41,46,48,50,51,54,61,64,81,85]
ANAPC7 [85]
AP1S3 [8,34,36,40]
AP3B1 [8,34,36,40,55]
AP3D1 [8,34,36,40]
APOB [2,5,6,7,44,86,104,107]
APOBEC4 [38]
APOE [1,2,5,6,7,8,11,12,1 16,17,23,44,53,54,57,60,62,70,73,80,86,92,94,95,104,107]
MRPL37 [30]
MRPL9 [30]
MRPS14 [30]
MRPS18C [30]
MRPS6 [30]
MT1H [44]
MT3 [31,44,53,60,62,80,102,105]
MTA2 [1,3,4,11,12,13,14,15,16,17,18,19,20,25,26,27,28,32,33,47,75]
MTIF3 [2,23,29,30,45,63]
MUL1 [5,6,9,10,21,22,23,46,52,57,65,71,73,77,87,88,93]
MXD4 [3,4,11,13,14,18,19,20,25,32,33,47]
MYBBP1A [8]
MYH11 [2,7,29,35,69]
MYO6 [1,4,8,12,16,17,26,28,34,36,40,55,96,110,113]
N6AMT1 [57]
NAA15 [1,12,16,17,26,28]
NAB2 [3,11,13,14,18,19,20,25,31]
NAIF1 [9,10]
NAP1L1 [2,7,15,24,27,29,35,59,75,76]
NAPG [2,7,8,34,36,40,45]
NASP [2,15,24,27,29,31,42,59]
NCAPG2 [15,37,3941,46,48,50,51,76]
NCBP1 [1,2,5,6,7,8,16,17,23,26,29,35,38,43,45,58,63,66,67,68,78,82,83,84,89,91,101,103,106,118]
NCK1 [2,7,23,29,35,45,56,63,69,90,97]
NCK2 [2,7,23,29,35,45,56,63,69,90,97]

APOL2 [86]
APTX [9,10,24,44,45,80]
AREG [1,12,16,17,31]
ARHGEF10L [1,4,12,16,17,26,28,56,72]
ARHGEF4 [9,10]
ARID5A [3,11,13,14,18,19,20,25,32,33]
ARPC1A [56,90,97]
AS3MT [44]
ASAH2 [9,10]
ASCL3 [49]
ASH1L [15,27,42,76]
ATF4 [1,4,12,16,17,26,28]
ATF7IP [1,3,4,11,12,13,14,16,17,18,19,20,24,25,26,28,32,33,47,90]
ATL2 [2,7,8]
ATM [5,6,9,10,24,37,41,56]
ATP5B [108,111,112]
ATP5D [8,44,108,111,112]
ATP5E [108,111,112]
ATPAF1 [2,7]
ATXN10 [9,10]
AUP1 [5,6,21,22]
AURKB [34,36,37,39,41,46,48,50,51]
AXIN2 [9,10,24]
BARHL2 [1,4,12,23,45,53,54,57,60,62,63,70,92,94,95]
BCL10 [1,2,7,9,10,12,16,17,23,26,28,52,54,61,71,77,81,85]
BLOC1S2 [1,12,16,17]
BMPR1A [1,23,53,54,60,61,62]
BNIP2 [9,10]
BRD1 [15,27,42]
BSCL2 [9,10]
BST2 [31,52,71,77]
BSX [1,4,12,16,17,26,28]
BTBD1 [5,6,21,22]
BYSL [58]
C14ORF169 [15,17,42]
C18ORF32 [52,71,77]
C9ORF89 [52,71]
CACNA1G [44,109,114]
CACYBP [5,6,21,22]
CADM1 [9,10,96]
CALM2 [44]
CAPZB [56,90,97,99]
CASP9 [9,10,87,88,93]
CASR [44]
CAV2 [2,7,29,35,56,65,69,90]
CBFA2T2 [3,11,13,14,18,19,20,25,32,33]
CBX1 [15,27,75]
CBX5 [3,11,13,14,15,18,19,20,25,27,32,33,75]
CCNO [5,6,24]
CCT3 [49]
CD2BP2 [38,43,66,67,68]
CD79A [31]
CD81 [1,23,31.54,61.73]
CDC16 [1,3,5,6,21,22,23,31,37,39,41,46,48,50,51,54,56,61,64,81,85]
CDCA2 [37,39,41,46,48,50,51]
CDH2 [2,7,53,60,62,70,92,94,95,96]
CDK10 [37,39]
CDK11B [9,10,31,37,39,41,46,48,50,51,57]
CDK5RAP1 [53,60,62,70]
CDKN2C [37,39,57]
CDT1 [3,11,13,14,18,19,24,59]
CDYL2 [15,27,75]
CELA3A [86]
CFL1 [56,97,98,99]
CFLAR [9,10,52,71,77]
CHD3 [4,15,27,42,75]
CHD4 [4,15,27,42,75]
CHD5 [15,27,42,75]
CHRM3 [31]
COG1 [8]
CPOX [44]
CPSF1 [38,43,66,67,68,118]
CRIP1 [31]
CSRP2BP [15,27,42]
CSTF2 [38,43,66,67,68,118]
NCOA4 [1,12,16,17,26,28]
NCOR1 [3,4,11,13,14,15,18,19,20,25,27,32,33,37,41,42,47,52,72,116,117]
NDUFAF3 [2,7,29,35,65,69,]
NENF [52]
NEURL3 [5,6,21,22]
NFAT5 [1,4,12,16,17,26,28]
NFIB [1,4,12,16,17,24,26,28,59]
NFX1 [3,4,5,6,11,13,14,18,19,20,21,22,25,32,33,47]
NFYA [1,4,12,16,17,26,28]
NFYC [1,4,12,16,17,26,28,49]
NHP2 [58]
NIPBL [15,37,39,41,46,48,50,51]
NISCH [9,10]
NKRF [3,11,13,14,18,19,20,25]
NKX2-5 [1,3,4,11,12,13,14,16,17,18,19,20,25,26,28,32,33,47,53,60,62,70,72,90,109,114]
NLGN1 [8,34,36,40,53,55,60,62,70,96]
NLGN2 [96]
NOL8 [24,57,59]
NOP14 [58]
NOSTRIN [3,11,13,14,18,19,20,25,32,33]
NOTCH3 [53,60,62,70]
NOV [57]
NPC2 [8]
NPEPL1 [8,34,36,40]
NR5A2 [1,4,12,16,17,26,28,72]
NR6A1 [3,4,11,13,14,18,19,20,25,31,32,33,47]
NRD1 [1,23,31,54,87,88,96]
NSA2 [58]
NSDHL [86]
NTN1 [9,10,53,57,60,62,70,92,94,95]
NUP107 [8,78,82,83,84,89,106]
NUP133 [2,8,29,78,82,83,84,89,106]
NUP160 [8,78,82,83,84,89,106]
NUP35 [78,82,83,84,89]
NUP54 [8,34,36,40,55,78,82,83,84,89]
NUSAP1 [15,37,39,41,46,48,50,51,56,76]
OCA2 [31]
OGFR [57]
OLA1 [108,111,112]
OOEP [2,7,29,35,69,98]
OPA1 [8,9,10,46,65]
OSGIN2 [37,41]
PAF1 [15,27,42]
PAFAH1B1 [8,31,37,39,41,46,48,50,51,98]
PAIP1 [23,30,38,45,63]
PCNP [5,6,21,22]
PCSK1 [44]
PDAP1 [31]
PDCD6 [9,10,44]
PELO [15,30,31]
PER1 [3,11,13,14,18,19,20,25]
PEX14 [2,3,7,8,11,13,14,18,19,20,25,32,33,34,36,40,55,72,100]
PEX6 [8,34,36,40,45,55]
PFKM [2,7]
PGAM2 [44]
PIAS4 [1,3,4,5,6,11,13,14,18,19,20,21,22,23,25,32,33,47,54,61]
PIF1 [56,115]
PIM2 [9,10,31,37,41,52,65,71,77]
PJA1 [5,6,21,22]
POLA2 [8,24,34,36,40,55,59]
POLG [24,59,65,79]
POU1F1 [1,4,8,12,16,17,26,28,72]
POU3F2 [1,4,12,16,17,26,28,31,53,60,62,70,92,94,95]
PPIA [24,49]
PPIC [49]
PPIE [38,43,49]
PPP2R1A [2,3,7,23,43,52,57,64,73]
PPP2R5C [5,6,21,22,]
PRDX2 [31,44,73,80,100,102,105]
PRKAA1 [11,12,13,73,86,104,107]
PRPF31 [2,7,29,35,38,43,58,66,67,68,91]
PSMA7 [1,3,5,6,21,22,23,39,54,61,64,81,85]
PSMB3 [1,3,5,6,21,22,23,39,54,61,64,81,85]
PSMC2 [1,3,5,6,21,22,23,39,54,61,64,81,85]
PSMC4 [1,3,5,6,21,22,23,39,54,61,64,81,85]

CSTF3 [38,43,66,67,68,118]
CWC27 [49]
CXCL1 [31]
CXCL12 [31,56,97]
CYB5R1 [86]
CYCS [5,6,9,10,24,87,88,93]
DARS [2,7,30]
DBN1 [53,60,62,70,94]
DCAF13 [58]
DCP1A [5,6,38,101,103]
DCTN1 [9,10,37,39,41,46,48,50,51]
DCUN1D3 [57]
DDAH1 [12,16]
DDX23 [2,7,29,35,38,43,58,66,67,68,91]
DFFA [5,6,9,10,24]
DFNB31 [110]
DGAT1 [2,7]
DHCR7 [86,104,107]
DHPS [30]
DHX9 [38,43,45,66,67,68]
DIABLO [9,10,87,88,93]
DLG1 [31,98]
DMPK [109]
DNAJA1 [49]
DNAJA3 [1,9, 10,23,24,45,49,52,54,59,61,65,71,79,81,85,87,88,93,96,100]
DNAJB1 [49]
DNAJB6 [3,11,13,14,18,19,20,25,32,33,49,87,88,93]
DNMT1 [1,3,4,11,13,14,15,18,19,20,23,24,25,27,32,33,42,47,54,56,61,64]
DRAP1 [3,4,11,13,14,18,19,20,25,32,33,47]
DTD1 [30]
DTL [5,6,21,22,24,59]
DUSP6 [73]
DUSP8 [73]
DUT [24,59]
DYNLL1 [9,10]
E2F1 [1,3,4,9,10,11,12,13,14,16,17,18,19,20,25,26,28,31,32,33,37,39,47]
E2F3 [1,2,7,12,16,17,26,28]
EED [3,11,13.14,15,18,19,20,25,27,42]
EEF1A1 [30,74]
EEF1B2 [30,74]
EFTUD1 [30]
EIF2B3 [3,11,13,14,23,30,45,63,64]
EIF3E [3,5,6,11,13,14,23,30,38,45,63,64,101,103]
EIF3G [30]
EIF3K [23,30,45,63]
EIF3L [30]
EIF4A2 [23,30,45,63]
EIF4G2 [9,10,23,30,45,63]
ELP2 [4]
ENO1 [3,4,11,13,14,18,19,20,25,32,33,47,57]
ENO3 [79]
ENY2 [1,12,15,16,17,26,27,28,42,78,82,83,84,89]
EPC2 [15,24,27,42]
EPRS [2,7,30]
ERBB2 [31,52,73,96]
ERCC3 [1,2,4,5,6,7,12,16,17,24,26,28,108,111,112]
ERCC6L [37,39,41,46,48,50,51]
ERCC8 [1,16,17,24]
ESRP2 [38,43]
ESRRB [31]
EVL [2,29]
EXOC5 [8]
EXTL3 [57]
F2R [1,8,9,10,12,16,17,26,28,34,36,40,52,55,71,77,87,88,93,96]
FAM83D [37,39,41,46,48,50,51]
FANCG [24,65]
FASTKD1 [9,10]
FBXL20 [5,6,21,22]
FBXL6 [56,21,22]
FBXO10 [5,6,21,22]
FBXO3 [5,6,21,22]
FHL2 [1,4,12,16,17,26,28]
FKBP2 [49]
FKBP4 [2,7,29,35,49,69]
FKBP9 [49]
PSMD1 [1]
PSME2 [1,3,5,6,21,22,23,39,54,61,64,81,85]
PTGES3 [15,24]
PTPN11 [73]
PTPRK [3,11,13,14,18,19,20,25,34,36,44,80,102,105]
PTPRU [3,11,13,14,18,19,20,25,34,36]
PTRH1 [30]
PTTG1IP [8,34,36,40]
PTTG1IP [55]
PTTG3P [15,24]
PURB [3,11,13,14,18,19,20,25,32,33]
PXDN [44,80,102,105]
PXN [2,7,29,35,44,69,80,102,105]
QPRT [2,7]
QSOX1 [49]
RAB3A [56,65]
RAB8A [8]
RABGEF1 [5,6,21,22]
RAD54L [15,24,37,41]
RANBP3 [8]
RAP1B [31]
RBBP4 [15,24,27,42,59]
RBM10 [38,43]
RBM14 [1,4,12,15,16,17,24,26,27,28,38,42,43,59]
RBM23 [38]
RBM8A [5,6,23,38,43,45,63,66,67,68,78,82,83,84,89,101,103]
RBMS1 [24,59]
REC8 [15,24,37,41]
RECQL5 [24]
RFC2 [24,59]
RFC4 [24,59]
RFC5 [24,59]
RFC5 [59]
RMI1 [24,59]
RNASET2 [5,6]
RNF10 [1,12,16,17]
RNF130 [9,10]
RNF149 [5,6,21,22]
RNF216 [5,6,8,10,21,22]
ROBO2 [53,60,62,70,92,94,95]
ROMO1 [44,79,80,102,105]
RPA3 [5,6,24,59]
RPF1 [58]
RPL19P12 [30,74]
RPL21 [30,74]
RPL23A [30,74]
RPL24 [2,7,29,30,35,37,39,41,48,58,74,91]
RPL39 [30,74]
RPL5 [30,58,74]
RPL7 [30,58,74]
RPS14 [2,3,4,7,11,13,14,18,19,20,25,29,30,32,33,35,47,58,74,91]
RPS15A [30,74]
RPS2 [30,74]
RPS23 [30,74]
RPSA [30,74]
RRP8 [58]
RTP3 [8,34,36,40,55]
RUFY3 [53,60,62,70,92,94,95]
RUNX3 [31]
RUVBL2 [15,24,27,42,49]
RYR2 [8,44,109,11]
S100A11 [3,11,13,14,18,19]
SAC3D1 [37,39,41,46,48,50,51]
SATB1 [3,4,11,13,14,15,18,19,20,25,27,31,33,47]
SCYL1 [8]
SDHAF1 [2,7,29,35,65,69]
SECISBP2 [30]
SELT [23,30,45,63,74]
SERPINF1 [31,53,60,62]
SERTAD2 [1,12,16,17,26,28,57]
SFRP5 [9,10,98]
SHARPIN [9,10,65]
SHB [9,10,31]
SHISA5 [9,10,25,71,77]
SIRT3 [3,11,13,14,18,19,20,25,32,33]

FNTA [1,3,11,12,14,23,44,54,61]
FOXE1 [1,3,4,11,12,13,14,16,17,18,19,20,25,26,28,32,33,47]
FOXJ2 [1,12,16,17,26,28]
FRG1 [38,43,58]
FXR1 [9,10]
GABARAP [8,34,36,40,55]
GABPB1 [4]
GBF1 [8]
GCG [31]
GEMIN7 [2,7,29,35,38,43,58,66,67,68,91]
GFM1 [30,65,74]
GFM2 [2,29,30,65]
GLRX2 [9,10,24,44,49,80]
GPR89B [52,71,77]
GRPEL1 [8,34,36,40,49,55,65]
GSS [44]
GTF2F2 [1,2,4,7,12,16,17,26,28,38,43,66,67,68]
GTF2H5 [24]
H1FX [2,7,15,27,29,35,75,76]
H2AFZ [2,7,15,27,29,35,75,76]
HAT1 [3,11,13,14,15,18,19,20,25,27,32,33,42,76]
HBZ [3,4,11,13,14,18,19,20,25,32,33,47]
HDAC3 [3,4,11,13,14,15,18,19,20,25,27,32,33,37,41,42,47,52,53,72,116,117]
HEATR1 [58]
HELT [3,4,11,13,14,18,19,20,25,32,33,47,79]
HERC3 [5,6,21,22]
HES5 [1,3,4,11,12,13,14,16,17,18,19,20,25,26,28,32,33,47,53,60,62,70,72,110,113]
HFE [2,7,]
HIST1H2AD [2,7,15,27,29,35,75,76]
HIST1H2AI [2,7,15,27,29,35,75,76]
HIST1H2AK [2,7,15,27,29,35,75,76]
HIST1H2BA [2,7,15,27,29,35,75,76]
HIST1H2BF [2,7,15,27,29,35,75,76]
HIST1H2BI [2,7,15,27,29,35,75,76]
HIST1H4D [2,7,15,27,29,35,75,76]
HIST1H4K [2,7,15,27,29,35,75,76]
HIST3H3 [2,7,15,27,29,35,75,76]
HMG20B [15,27,42]
HMGCR [73,86]
HMOX1 [1,4,9,10,12,31,44,52,71,77,80,100,102]
HNRNPA2B1 [38,43,66,67,68,78,82,83,84]
HNRNPAB [1,3,11,12,13,14,16,17,18,19,20,25,26,28,32,33,72]
HNRNPC [38,43,66,67,68]
HNRNPL [38,43,66,67,68]
HRK [3,9,10,20]
HSBP1 [3,4,11,13,14,18,19,20,25,32,33,47]
HSP90AA1 [2,7,8,12,16,29,35,49,65,69]
HSP90AB1 [3,12,16,23,49,64]
HSPA5 [87,88,93]
HSPA8 [8,49]
HSPBP1 [49]
HSPD1 [2,7,9,10,24,29,31,35,45,49,69,87,88,93]
HTATIP2 [4,8,9,10]
HTRA1 [57]
HTRA2 [9,10,65]
HUWE1 [5,6,15,21,22,27,42]
IARS2 [30]
ID3 [3,4,11,13,14,18,19,20,25,32,33,47,100]
IFI6 [9,10,65,87,88,93]
IGF2 [1,3,9,10,11,12,13,16,17,23,52,53,54,56,57,61,64,72]
IGF2BP3 [3,11,13,14,23,30,45,63,64]
IGFBP2 [44,57,79]
IGFBP6 [57]
IL15RA [31]
INCENP [37,39,41,46,48,50,51]
INSIG1 [31,86]
IP6K2 [57]
IPO5 [2,7,8,29,34,35,36,40,55,69]
IRF7 [2,3,4,7,11,13,14,18,19,20 ,25,32,33,47]
ISG15 [5,6,21,22]
ITM2B [9,10]
JARID2 [3,4,11,13,14,18,19,20,25,32,33,47]
JDP2 [3,4,11,13,14,18,19,20,25,32,33,47]
KCTD13 [24,59]
KDELR2 [8,34,36,40]
SIRT5 [3,11,13,14,18,19,20,25,32,33]
SIX1 [1,4,12,16,17,26,28,43,60,62,70]
SLC1A1 [2,7]
SLC25A15 [8]
SLC5A11 [9,10]
SLC9A3R1 [2,7]
SMAD4 [1,2,3,4,7,11,12,13,14,16,17,18,19,20,23,25,26,28,29,32,33,35,53,54,57,61,69,92]
SMAD7 [1,3,4,11,13,14,17,18,20,23,25,32,33,45,47,53,54,61,64,72,81,85,92,100,109,114]
SMARCA5 [2,4,7,15,27,29,35,42,75,76]
SMARCAL1 [4,15,24,27,42]
SMARCB1 [1,4,12,15,16,17,25,26,27,28,42,72]
SMARCD1 [1,4,12,15,16,17,26,27,28,42]
SMC2 [15,37,39,41,46,48,50,51,76]
SMC4 [15,37,39,41,46,48,50,51,76]
SMG6 [5,6,8,15,24,38,78,82,83,84,89,101,103,106]
SMN1 [2,7,9,10,29,35,38,43,58,66,67,68,91]
SND1 [3,20,45]
SNRNP25 [38,43]
SNRNP40 [38,43,66,67,68]
SNRPD2 [2,7,29,35,38,43,58,66,67,68,91]
SNRPE [2,7,29,35,38,43,58,66,67,68,91]
SNX6 [3,8,11,13,14,18,19,20,25,34,36,40]
SOCS4 [5,6,21,22]
SOD1 [5,6,7,10,11,24,44,73,79,80,102,104,105,107,110,113]
SOX15 [1,3,4,11,12,13,14,15,16,17,18,19,20,25,26,27,28,32,33,47]
SPAG9 [2,7,73]
SPHAR [24,59]
SPHK2 [31]
SPP2 [2,7]
SPTBN2 [9,10,56,90,97,99]
SQSTM1 [1,4,5,6,8,9,10,12,16,17,21,22,26,28,52,71]
SREBF1 [1,4,12,16,17,26,28,86]
SRP14 [3,8,11,13,14,23,34,36,40,45,55,63,64]
SSR1 [8,34,36,40,55]
STAP1 [8,34,36,40]
STAR [44,53,60,62,80,104]
STARD3 [8,86]
STAT2 [4]
STAU1 [34,78]
STMN1 [2,29,37,39,41]
STX10 [8,34,36,40]
STX2 [8,34,36,40]
STX4 [2,7,8,34,36,40]
STX6 [8,34,36,40]
SUMO1 [3,5,6,11,13,14,18,19,20,21,22,24,25,90,100]
SURF1 [2,7,29,35,69]
SYT1 [2,7,44]
SYTL1 [8,34,36,40]
TADA3 [4,15,27,42]
TAF8 [1,12,16,17,26,28,31]
TARDBP [9,10,37,38,39,41,43,46,48,50,51]
TBCA [2,7,29,35,49,69]
TBX21 [1,16,17]
TCF3 [1,4,12,16,17,26,28,37,39,72]
TCP1 [2,7,29,35,49,69]
TERF1 [2,3,7,9,10,11,13,14,15,18,19,24,28,38,39,41,42,46,48,50,51,56,59,79,87,8890,93,115]
TESC [1,12,16,17,26,28,72]
TFIP11 [38,43]
TGFA [31,56,73]
TGIF1 [3,4,11,13,14,18,19,20,25,32,33,47,53,60,62,70]
THOC3 [8,38,43,78,82,83,84,89,106]
THPO [31]
THRAP3 [1,2,4,7,12,16,17,26,28]
TIMELESS [3,11,13,14,18,19,20,25,37,39,41,46,48,50,51]
TIMP3 [3,23,64,79]
TLK2 [15,27,42,56]
TLR3 [1,12,52,71,77]
TLR6 [1,12,23,52,71,73,77]
TLR9 [1,12]
TMSB4X [56,90,97]
TNFAIP8 [9,10]
TNK2 [1,23,54,61]
TOMM20L [8,34,36,40,55]
TOMM40 [8,34,36,40,55,65]
TOP1 [9,10,24,59]

KDM4A (15,27,42)
KDM4B (15,27)
KDM4C (15,27,42)
KHSRP (38,43,78,82,83,84,89)
KIF1A (8)
KIF2C (2,29,31,37,39,41,46,48,50,51)
KIFC1 (15,37,39,41,46,48,50,51)
KPNB1 (2,7,8,29,34,35,36,40,55,69)
LAMA1 (98)
LBX1 (4,53,60,62,70,72)
LDLRAP1 (86)
LETM1 (65)
LFNG (37,41)
LHFPL5 (110,113)
LIMA1 (56,97,99)
LINGO1 (53,60,62,70,92,94,95)
LITAF (4,9,10,52,71,77)
LOC643733 (9,10)
LRRC29 (5,6,21,22)
LRRC4C (53,60,62,70,92,94,95)
LRRK2 (1,23,54,61,81,85)
LTBP4 (49,57)
LUZP6 (23,45,63)
M6PR (8)
MAF (1,4,12,16,17,26,28)
MAFB (1,4,12,16,17,26,28)
MAP1B (44,53,56,57,60,62,70,90,92,94,95,96,99)
MAP1LC3B (5,6,21,22)
MAP2K1 (1,2,4,7,8,16,17,26,31,37,39,41,46,48,50,51,73)
MAP3K5 (9,10,23,52,73)
MAP4 (56,99)
MAP7 (98)
MAPK15 (1,3,11,13,14,18,19,23,54,61,81,85)
MARK2 (98)
MARS (30)
MCF2L (9,10)
MCM3AP (8,24,34,36,40,55,59)
MCOLN3 (110,113)
MED11 (4)
MEF2C (1,3,4,9,10,11,12,13,14,16,17,18,19,20,25,26,28,32,33,47,72)
MEF2D (9,10)
MEN1 (1,3,4,11,12,13,14,15,16,17,18,19,20,23,24,25,26,27,28,32,33,42,47,52,54,56,61,64,87,88,93,100,115,116,117)
METAP1 (23,25,63)
MGEA5 (1,3,5,8,9,10,11,13,14,16,17,23,34,36,40,54,55,64,79,99)
MGRN1 (5,6,21,22)
MIF4GD (23,45,63)
MKNK2 (23,45,63)
MLPH (8,34,36,40,55)
MMD (9,10)
MNT (79)
MNX1 (4)
MORF4L2 (1,4,12,15,16,17,24,26,27,28,42,57)
MORF4L2 (4,12,15,16,17,24,26,27,28,42,57)
MPDU1 (49)
MRPL17 (30)
MRPL23 (30)
TOR2A (49)
TPRA1 (79)
TRIP12 (5,6,21,22)
TRNAU1AP (23,30,45,63,74)
TSPAN6 (52,71,77)
TSPO (8,9,10,31,34,36,40,55,65)
TSR1 (2,7,29,35,58,91)
TTC9C (49)
TUBA1C (2,7,29,35,69)
TUBB2A (2,7,29,35,37,39,41,46,48,50,51,69)
TUBB6 (2,7,29,35,69)
TXLNA (31)
TXNL4A (2,7,29,35,37,38,39,41,43,46,48,50,51,58,66,67,68,91)
UBAC1 (5,6,21,22)
UBE2E3 (5,6,21,22)
UBE2G2 (5,6,21,22)
UBE2I (3,5,6,11,13,14,18,19,20,21,22,25,31,33,37,39,41,46,48,50,51)
UBE2M (5,6,21,22)
UBE2S (5,6,21,22)
UBE2V1 (5,6,21,22,53,71,77)
UBE3A (5,6,21,22)
UBE4A (5,6,21,22)
UBE4B (5,6,9,10,21,22)
UBN1 (4,15,27,42)
UFC1 (5,6,21,22)
UNC5B (9,10)
UPF1 (5,6,8,23,24,38,45,59,63,78,82,83,84,89,99,101,103,106)
USF2 (1,4,12,16,17,26,28,72)
USP16 (1,2,5,6,7,12,15,16,17,24,22,26,27,28,37,39,41,42,46,48,50,51)
USP32 (5,6,21,22)
USP36 (5,6,21,22)
USP39 (2,5,6,7,21,22,29,35,38,43,58,66,67,68,91)
USP42 (5,6,21,22)
USP48 (5,6,21,22)
USP54 (5,6,21,22)
USP9X (5,6,21,22,37,39,41,46,48,50,51)
UTP3 (3,15,20,28,42)
VDAC1 (9,10)
VDR (3,4,11,13,14,18,19,20,25,32,33,47,72)
VPS35 (8)
VWF (2,7)
WISP1 (57)
WNT1 (1,12,16,17,26,28,98)
WSB1 (5)
WSB1 (6,21,22)
WTAP (38,43)
XPO7 (2,7,8,29,34,35,36,40,55,69,78,82,83,84,89)
XRCC3 (24)
YWHAB (2,3,7,8,9,10,11,13,14,18,19,20,23,25,34,36,40,55,64)
YWHAZ (8,34,36,40,55,65)
ZAK (9,10,23,31,52,73)
ZFP36L1 (5,6,23,38,45,63,401,103)
ZFP36L2 (5,6,31,38,45,101,103)
ZHX3 (3,11,13,14,18,19,20,25,32,33)
ZNF462 (1,4,12,16,17,26,28,100)

Microarray analyses of hiPSC-derived motor neurons from sporadic ALS and non-ALS subjects. Motor neurons were differentiated of hiPSC reprogrammed from fibroblasts obtained from ALS and non-ALS subjects as described in the text. The numbers between parentheses indicate the Biological Process GO term(s) in which the genes are included.
